# Evaluation of strategies for the assembly of diverse bacterial genomes using MinION long-read sequencing

**DOI:** 10.1186/s12864-018-5381-7

**Published:** 2019-01-09

**Authors:** Sarah Goldstein, Lidia Beka, Joerg Graf, Jonathan L. Klassen

**Affiliations:** Department of Molecular and Cell Biology, University of Connecticut, Storrs, CT USA

**Keywords:** Oxford Nanopore MinION, Genome sequencing, Genome assembly, Secondary metabolites, Insertion sequences

## Abstract

**Background:**

Short-read sequencing technologies have made microbial genome sequencing cheap and accessible. However, closing genomes is often costly and assembling short reads from genomes that are repetitive and/or have extreme %GC content remains challenging. Long-read, single-molecule sequencing technologies such as the Oxford Nanopore MinION have the potential to overcome these difficulties, although the best approach for harnessing their potential remains poorly evaluated.

**Results:**

We sequenced nine bacterial genomes spanning a wide range of GC contents using Illumina MiSeq and Oxford Nanopore MinION sequencing technologies to determine the advantages of each approach, both individually and combined. Assemblies using only MiSeq reads were highly accurate but lacked contiguity, a deficiency that was partially overcome by adding MinION reads to these assemblies. Even more contiguous genome assemblies were generated by using MinION reads for initial assembly, but these assemblies were more error-prone and required further polishing. This was especially pronounced when Illumina libraries were biased, as was the case for our strains with both high and low GC content. Increased genome contiguity dramatically improved the annotation of insertion sequences and secondary metabolite biosynthetic gene clusters, likely because long-reads can disambiguate these highly repetitive but biologically important genomic regions.

**Conclusions:**

Genome assembly using short-reads is challenged by repetitive sequences and extreme GC contents. Our results indicate that these difficulties can be largely overcome by using single-molecule, long-read sequencing technologies such as the Oxford Nanopore MinION. Using MinION reads for assembly followed by polishing with Illumina reads generated the most contiguous genomes with sufficient accuracy to enable the accurate annotation of important but difficult to sequence genomic features such as insertion sequences and secondary metabolite biosynthetic gene clusters. The combination of Oxford Nanopore and Illumina sequencing can therefore cost-effectively advance studies of microbial evolution and genome-driven drug discovery.

**Electronic supplementary material:**

The online version of this article (10.1186/s12864-018-5381-7) contains supplementary material, which is available to authorized users.

## Background

Microbial genome sequencing has revealed how microorganisms adapt, evolve, and contribute to health and disease [1, 2]. Although these were once enterprise-level projects, technological advances have now reached the point where microbial genomes can be sequenced routinely by small teams for a few hundred dollars [1]. These advances have particularly been driven by the maturation of short-read sequencing technologies such as those marketed by Illumina, which generate highly accurate reads (> 99%) with lengths ranging from 75 to 300 bp [1]. Although Illumina technologies currently dominate the sequencing market [1, 2], difficulties remain that require further technological advances to fully realize the potential of microbial genome sequencing.

By their very nature, short reads alone cannot disambiguate repetitive genomic regions that are longer than their read length. Unfortunately, such repetitive regions are common in microbial genomes [3–6], and include ribosomal genes, transposons, insertion sequences, CRISPR arrays, *rhs* toxins, secondary metabolite biosynthetic gene clusters, and many others [5]. Repeats lead to unresolvable loops in the underlying genome assembly graph that are ultimately fragmented into contigs [5, 7]. Because of this, short reads are theoretically incapable of closing most microbial genomes.

Genome assembly using most short-read datasets is also challenged by biases that occur during library preparation and that cause some genomic regions to be excluded from the final sequencing library. Common short-read library preparation methods (e.g., the Illumina Nextera protocol) include PCR amplification steps that are biased against regions of the genome with extreme GC contents [8–12]. Such regions are common In bacteria, whose average GC content ranges widely from 25 to 75% [13]. Library preparation protocols that use transposases to fragment DNA may also non-randomly shear genomes during library preparation [14], causing further biases that limit the utility of short-read sequencing.

De novo genome assembly algorithms struggle to assemble genomes when intergenic repeats are present and GC biases skew sequencing coverage [15, 16]. Fragmentation of such genomes prevents the accurate identification of mobile elements, the detection of horizontal gene transfers, the determination of gene copy number, and the discovery of biotechnologically important gene clusters such as those that encode for the production of secondary metabolites [16, 17]. These deficiencies significantly lower the informational value of draft-quality genomes [18, 19].

Recently, long-read, single-molecule sequencing has overcome some of the deficiencies of short-read sequencing. Library preparation protocols for single-molecule sequencing typically avoid bias-prone PCR steps, and long read lengths span genomic repeats to unambiguously resolve complex genomic regions. Some Illumina-based technologies such as mate pair libraries and linked reads (e.g., as commercialized by 10X Genomics) can also generate positionally linked sequences that span complex genomic repeats [1], but these technologies still require library preparation protocols that are subject to the biases discussed above. Pacific Biosciences (PacBio) currently markets the most widely used single-molecule sequencing technology, which can produce > 7 Gb per run with read lengths averaging > 12 kbp [1]. Although the error rate for PacBio sequencing is high (~ 13%), these errors are near-randomly distributed and can largely be corrected during assembly with adequate sequencing coverage [7]. Unlike some Illumina sequencers (e.g., the MiSeq and MiniSeq), all PacBio sequencers require considerable capital investment, limiting general access to these technologies in individual laboratories. Nevertheless, PacBio sequencing has shown the enormous potential for long-read, single-molecule sequencing to routinely produce high-quality microbial genome assemblies that overcome many of the deficiencies of short-read sequencing.

The Oxford Nanopore Technologies (ONT) MinION is a more recently developed long-read, single-molecule sequencing instrument. The MinION is a small benchtop device that can plug directly into a laptop via a USB3 port [20] and requires a relatively small upfront financial investment relative to PacBio instruments [1]. This affordability and simplicity has enabled the rapid uptake of MinION sequencing by individual labs worldwide and facilitated new applications such as tracking disease outbreaks in low-resource environments [21]. MinION read lengths have no theoretical limit and reads > 2 million bp long have been reported [22]. As with PacBio, MinION read quality is low compared to short-read sequencing technologies [23, 24]. These errors are less randomly distributed than for PacBio sequencing [25], meaning that increased read depth alone cannot completely overcome this high error rate, at least currently. However, error rates and bias profiles are expected to improve as the MinION and its associated base-calling software continues to develop, e.g., as demonstrated by the increased accuracy of new ONT base callers [26].

Two main strategies have been used to assemble bacterial genomes using MinION sequencing [27, 28]. In the first, MinION reads are used to enhance genome assemblies that are generated from short-read Illumina data. Here, MinION reads can scaffold contigs generated by Illumina sequencing [29–31] or be directly used in the assembly process to disambiguate regions of the assembly graph that cannot be resolved by Illumina sequencing alone (e.g., as implemented in the popular SPAdes and Unicycler software [32, 33]). Alternatively, MinION reads alone are used to generate an initial genome assembly [34, 35] that can then be further polished using either MinION or Illumina reads [34, 36]. Such polishing is highly recommended for MinION-based genome assemblies due to their higher error rates relative to assemblies based on Illumina data [17, 26, 27, 37, 38]. The increasing maturity and throughput of MinION sequencing is leading to its adoption for routine microbial genome sequencing [39–41].

Both MinION-only [34, 35] and Illumina-hybrid methods [32, 33] have been validated extensively for bacteria with low and average GC contents. However, whether these approaches offer advantages when assembling bacterial genomes with high GC content remains unclear [42] (but see [43]). We therefore compared the ability of Illumina and MinION sequencing technologies to produce high-quality assemblies of genomes from three bacterial genera (*Flavobacterium*, *Aeromonas*, and *Pseudonocardia*) that range in GC content from 31 to 73% (Table 1). *Flavobacterium* spp*.* are gliding bacteria that can be found in diverse environments and that include important fish pathogens. *Aeromonas* spp. are ubiquitous in aquatic environments and can cause diseases in humans and fish or form beneficial symbioses, e.g., with fish and leeches [44]. *Pseudonocardia* sp. are members of the Actinobacteria and important producers of antibiotics such as those involved in defensive symbioses with ants (e.g., [45]). Our results validate MinION sequencing’s ability to generate high-quality assemblies for all of these genomes, and especially emphasize the advantages of MinION sequencing when unbiased Illumina sequencing libraries are difficult to generate, e.g., for Actinobacteria with high GC content. These improved genome assemblies dramatically improve the annotation of repetitive genomic regions such as insertion sequences and secondary metabolite biosynthetic gene clusters (BGCs). MinION sequencing therefore has strong potential to overcome current limitations of short-read sequencing technologies and catalyze improved understanding of genome evolution and exploitation of genomic data for drug discovery.

**Table 1:** Bacteria used in this study

Strain ID	Phylum	Genus	Species	% GC Content	Expected Genome Size (Mbps)
*Ps* JKS002128	Actinobacteria	*Pseudonocardia*	*sp*	73.12	6.60
*Ps* JKS002072	Actinobacteria	*Pseudonocardia*	*sp*	73.69	6.21
*Ps* JKS002056	Actinobacteria	*Pseudonocardia*	*sp*	73.31	6.54
*Av* JG3	Proteobacteria	*Aeromonas*	*veronii*	58.64	4.49
*Av* CIP107763^T^	Proteobacteria	*Aeromonas*	*culicicola* ^*a*^	58.80	4.34
*Ah* CA-13-1	Proteobacteria	*Aeromonas*	*hydrophila*	61.29	4.76
*Fs* ARS-166-14	Bacteroidetes	*Flavobacterium*	*sp*	31.61	3.31
*Fc* FC-100715-19	Bacteroidetes	*Flavobacterium*	*columnare*	31.59	3.32
*Fc* FC-08-1215-1	Bacteroidetes	*Flavobacterium*	*columnare*	31.56	3.31

^a^CIP107763^T^ is the type strain for *Aeromonas culicicola,* which is a later subjective synonym of *A. veronii*

## Methods

### Description of strains

Three *Aeromonas* strains were used in this study. *Aeromonas hydrophila* str. CA-13-1 (hereafter *Ah* CA-13-1) was isolated from the wound of a patient undergoing post-operative leech therapy in 2013 [46]. *Aeromonas veronii* str. CIP107763^T^ (hereafter *Av* CIP107763^T^) was isolated from a mosquito midgut in France in 2015 and sequenced previously [47]. *A. veronii* str. JG3 (hereafter *Av* JG3) is a derivative of a medicinal leech isolate Hm21 [48]. All *Aeromonas* strains were grown either in LB broth or on LB agar plates for 16 h at 30 °C [49].

The *Flavobacterium* strains used in this study were all isolated from necrotic gill tissues of farmed rainbow trout, *Onchorhyncus mykiss,* during diagnosis of diseased fish. *Flavobacterium* sp. str. ARS-166-14 (hereafter *Fs* ARS-166-14) was isolated in October 2014, *Flavobacterium columnare* str. FC-081215-1 (hereafter *Fc* FC-081215-1) was isolated in August 2015, and *F. columnare* str. FC-100715-19 (hereafter *Fc* FC-100715-19) was isolated in October 2015, all on TYES agar. Frozen cells were grown on TYES agar, incubated for three days at 20 °C, and then grown in liquid TYES broth for another 3 days at 15 °C (for *Fs* ARS-166-14) and 25 °C (for *Fc* FC-100715-19 and *Fc* FC-08-1215-1) [50].

The *Pseudonocardia* bacteria sequenced during this study were isolated from individual *Trachymyrmex septentrionalis* ants collected from three locations within the United States: Paynes Creek Historic State Park, FL (*Pseudonocardia* sp. str. JKS002056, hereafter *Ps* JKS002056), Magnolia Springs State Park, GA (*Pseudonocardia* sp. str. JKS002072, hereafter *Ps* JKS002072), and Jones Lake State Park, NC (*Pseudonocardia* sp. str. *Ps* JKS002128). *Pseudonocardia* were visible as white patches on the ants’ propleural plates, which were scraped using a sterile needle under a dissecting microscope to isolate *Pseudonocardia* on Chitin and YMEA agar media at 20 °C following Marsh et al. [51].

### DNA isolation

DNA was extracted from *Aeromonas* and *Flavobacterium* isolates following a modified version of a previously published protocol for large scale genomic DNA isolation [52, 53]. DNA in solution was not micropipetted during these extractions to minimize DNA fragmentation. DNA was extracted from single *Pseudonocardia* colonies using the Epicentre MasterPure Complete DNA and RNA kit following the manufacture’s protocol. Each *Pseudonocardia* extraction was performed in triplicate using wide bore tips and taking care to pipette slowly to prevent DNA shearing.

### Library preparation and sequencing

The quality of all extracted DNA was assessed using an Agilent TapeStation 2200 protocol for genomic DNA, an Agilent 2100 Bioanalyzer (High sensitivity DNA chip), and/or a Nanodrop spectrophotometer. All libraries were quantified using a Qubit® 2.0 fluorometer. For the *Aeromonas* and *Flavobacterium* strains, NexteraXT Illumina sequencing libraries were constructed by following the manufacturer’s instructions for genomic tagmentation, PCR of tagged DNA, and PCR product cleanup. Libraries were diluted to 4 nM for loading onto an Illumina MiSeq. TruSeq DNA PCR-Free libraries were created for each *Pseudonocardia* strain following the manufacturer’s protocol, shearing the DNA to 550 bp fragments using a Covaris M22 Focused-ultrasonicator. All Illumina libraries were sequenced on an Illumina MiSeq using the 2x250bp protocol at the University of Connecticut Microbial Analysis Research and Services (MARS) facility. Demultiplexing was performed using Illumina Basespace (https://basespace.illumina.com/home/index).

All genomes were sequenced on a MK1B MinION device using R9.4 flow cells. Four genomes (*Ah* CA-13-1, *Av* CIP 107763 T, *Fs* ARS-166-14, and *Fc* FC-100715-19) were also sequenced a second time on an R9.5 flow cell to increase coverage. *Aeromonas* and *Flavobacterium* libraries were prepared using the SQK-LSK108 Ligation Sequencing kit in conjunction with the PCR-Free ONT EXP-NBD103 Native Barcode Expansion kit following the ONT “Native Barcoding Genomic DNA Sequencing for the MinION Device” protocol (downloaded from https://nanoporetech.com/resource-centre/protocols on Oct 20, 2017) and performed without optional shearing steps to select for long reads. These genomes were sequenced on separate MinION flowcells loaded with either: (i) *Av* JG3 by itself (not barcoded); (ii) all three *Aeromonas* genomes, barcoded and sequenced together with one other *Aeromonas* genome (not discussed here); (iii) all three *Flavobacterium* genomes, barcoded and sequenced together on one flow cell; and (iv) *Ah* CA-13-1, *Av* CIP107763T, *Fs* ARS-166-14 and *Fc* FC-100715-19, barcoded and sequenced together. *Pseudonocardia* libraries were prepared using the ONT “1D gDNA Selecting for Long Reads Using SQK-LSK108” protocol (downloaded from https://nanoporetech.com/resource-centre/protocols on Dec 20, 2016). Each *Pseudonocardia* strain was sequenced on an individual flow cell. All strains were sequenced using the ONT MinKNOW NC_48h_Sequencing_Run_FLO-MIN106_SQK-LSK108 protocol. The run duration ranged from 12 to 48 h. Strains *Av* JG3, *Fc* FC-100715-19, and *Ps* JKS002072 were sequenced using two separate MinION runs that were combined for all analyses, except for the *Av* JG3 Canu+Nanopolish assembly where the few MinION reads (< 3000) from the first run were excluded because of their being processed using base calling software that was incompatible with Nanopolish.

### Base calling and read preparation

MinION reads for *Ps* JKS002056 and the first *Av* JG3 run were base-called using the ONT Metrichor 1D protocol and locally using MinKNOW (ONT; Oct 20, 2017 release), respectively. All other MinION reads were based-called using Albacore (v.1.2.4). These software choices were determined by changes made by ONT to their cloud-based base calling system. All raw data was deposited in the NCBI database under the BioProject number PRJNA477342.

We assessed Illumina read quality using FastQC (v.0.11.5, available from http://www.bioinformatics.babraham.ac.uk/projects/fastqc/). Trimmomatic (v.0.36) [54] was used remove Illumina adapters, bases at 3′ end of each read with an average Phred score < 15 over a 4 bp window, and reads ≤36 basepairs long. Poretools (v.0.6.0) [55] was used to assess the quality of each MinION dataset and to generate fastq files from basecalled fast5 files. Barcodes and reads that contained an internal barcode adapter sequence were removed using Porechop (v.0.2.3, available from https://github.com/rrwick/Porechop). Nanofilt (v.1.0.5, available from*,* https://github.com/wdecoster/nanofilt) was used to remove reads shorter than 500 basepairs or having an average quality score < 9.

### Genome assembly

We used several approaches to construct de novo assemblies of each genome. First, we constructed MiSeq-only short-read assemblies using SPAdes (v.3.11.1) [33] or Unicycler (v.0.4.3) [32], representing the current state of the art. Second, we added MinION reads to these MiSeq-based assemblies to disambiguate ambiguous regions in the MiSeq sequencing graph, creating SPAdes-hybrid and Unicycler-hybrid assemblies. Third, we constructed MinION-only long-read assemblies using Canu (v.1.5) [35]. These MinION-only Canu assemblies were polished using the same MinION reads to create Canu+Nanopolish assemblies by aligning MinION reads to the Canu assembly using BWA (v.0.7.15) [56] and Samtools (v.1.3.1) [57], and then using Nanopolish (v.3.2.5) [34] for assembly polishing. A second iteration of Nanopolish was completed for strain *Ps* JKS002128 but did not significantly improve its accuracy (data not shown) and so this strategy was not pursued further. The Canu assemblies were alternatively polished using MiSeq reads to create Canu+Pilon assemblies. MiSeq reads were aligned to the Canu genome using BWA (v.0.7.15) and then Pilon (v.1.22) [36] was used for assembly polishing. In total, we created seven assemblies for each genome: four based primarily on MiSeq data (SPAdes, Unicycler, SPAdes-hybrid, and Unicycler-hybrid) and three based primarily on MinION data (Canu, Canu+Nanopolish, Canu+Pilon). All commands used for the computational analyses in this study are included in Additional file 1.

### Depth of coverage

MinION data was subsampled from *Av* JG3, *Fs* ARS-166-14, and *Ps* JKS002128 to determine the minimum read depth required to create contiguous MinION-based assemblies. Fast5-formatted reads for each strain were subsampled in the order that they were acquired from the MinION sequencer to achieve 10X, 20X, 30X, 40X, 50X, (for *Fs* ARS-166-14, Av JG3 and *Ps* JKS002128), 60X (*Fs* ARS-166-14 and *Ps* JKS002128 only), and 70X (*Ps* JKS002128 only) coverage of the Canu assembly for each strain, calculated using the mean MinION read length for that strain (Table 2). This strategy was used to simulate runs stopped after achieving each level of coverage. All data was processed and assembled using Canu as described above.

**Table 2:** Summary of MinION sequencing

Strain ID	Total Raw Reads	Total bases (Mbps)	Mean Length (bps)	Median Length (bps)	Max Length (bps)	N50 (bps)	Coverage (fold)	Total Reads After Filtering
*Ps* JKS002128	119,358	499	9665	2510	244,268	7797	80	87,836
*Ps* JKS002072	135,898	311	2289	729	678,379	7142	50	70,035
*Ps* JKS002056	41,096	397	4184	6207	105,595	16,572	64	21,874
*Av* JG3 (run1)	2718	25	7232	5710	85,387	17,143	5	34,473*
*Av* JG3 (run2)	42,301	306	9176	4807	90,470	11,741	63	
*Av* CIP107763^T^	200,362	645	1629	1299	98,351	7545	135	110,391
*Ah* CA-13-1	136,486	222	1629	808	62,567	2840	46	65,195
*Fs* ARS-166-14	53,171	289	5442	1583	1,149,252	18,107	90	36,648
*Fc* FC-100715-19 (run1)	39,376	146	3709	836	84,881	17,593	45	45,194*
*Fc* FC-100715-19 (run2)	31,121	187	5996	1137	157,214	26,227	58	
*Fc* FC-08-1215-1	39,938	236	5908	1252	106,525	22,063	74	26,486

* indicates the combined total of both runs for that strain

### Quality assessment

The contiguity and quality of each genome assembly was assessed using Quast (v.4.6.3) [58]. Because we lacked reference genomes for comparison, we instead assessed the quality of our genomes using several strategies. First, we compared all *Pseudonocardia* genome assemblies to each other based on their shared k-mer composition using Mash (v2.0) [59]. These Mash distances were used to construct a phylogeny using Mashtree (v.0.33, available at https://github.com/lskatz/mashtree). We also aligned all assemblies to their respective Canu+Pilon assembly using MUMmer (v3.1) [60] to identify SNPs and indels relative to the Canu+Pilon assembly. We selected the Canu+Pilon assemblies as references because of their high contiguity and error profiles that were similar to the MiSeq assemblies. However, we stress that the relative nature of these comparisons does not comprise a perfect “gold standard” reference.

The BLAST Ring Image Generator (BRIG v.0.95) [61] software was used to compare all seven assemblies of strain *Ps* JKS002128 and determine the genomic contexts in which breaks in these assemblies occurred. The Canu+Pilon assembly was used as the reference for all alignments and the BRIG analysis was completed following the developer’s protocol. All assemblies were loaded into BRIG in FASTA format as a single concatenated sequence.

The local sequence context surrounding each SNP and indel detected in the nucmer analyses was assessed to detect systematic biases in our MinION sequencing data. The 5 base k-mer surrounding each SNP and indel position was tabulated, along with the length of any homopolymer in which these SNPs and indels were imbedded. Analyses were calculated separately for both the nucmer query and reference genome to accommodate our lack of a true “gold-standard” reference that would allow sequencing errors to be unequivocally identified. In practice, all results were consistent regardless of the direction in which comparisons were performed. The frequency of k-mers and homopolymers that surrounded SNPs and indels were compared to their corresponding assembly-wide frequencies to identify k-mers and homopolymers that were overrepresented among positions containing SNPs and indels relative to the rest of the genome. The scripts used to conduct these analyses are included in the Supplementary Materials.

SNPs and indels can introduce stop codons that create errors during gene annotation. We therefore compared the effect of genome assembly method on gene annotation using the Anvi’o (v5.2) pangenome pipeline [62, 63] (http://merenlab.org/2016/11/08/pangenomics-v2/). All genome assemblies were annotated using Prokka (v1.11) [64] and imported into separate Anvi’o databases for each strain using the gff_parser.py script (http://merenlab.org/2017/05/18/working-with-prokka/). HMM models were computed using the anvi-run-hmms command as described in the Anvi’o Metagenomics workflow (http://merenlab.org/2016/06/22/anvio-tutorial-v2//#anvi-gen-contigs-database) to calculate gene completion and redundancy, which are measures of gene fragmentation. Similarly, we calculated the average gene length, the number of genes annotated per kb, and the number of singleton gene clusters that contain genes that are unique to a single assembly method. Together these metrics show how errors in genome assembly can affect genome annotation.

### Biosynthetic gene cluster prediction

Secondary metabolite biosynthetic gene clusters (BGCs) were annotated in each *Ps* JKS002128 assembly using antiSMASH (v.4.1.0) [65]. Fragmented BGCs were annotated by their occurring at contig ends. This likely overestimates the number of fragmented BGCs due to antiSMASH’s tendency to conservatively extend BGCs past their true boundaries. Identical BGCs were identified using the ClustCompare pipeline (available from, https://github.com/klassen-lab/ClustCompare). Briefly, PfamScan (v.1.6) [66] was used to annotate protein domains encoded by each BGC and these domains were compared to each other using BLASTp [67]. BGCs were considered to be homologous based on their sharing a minimum ClustCompare similarity score of 0.3, calculated using a 70% similarity threshold between domains in different BGCs, a minimum of two homologous domains shared between BGCs, and a minimum of 50% of the domains in the smaller BGC being homologous to domains in the larger BGC. The resulting homology networks were visualized using Cytoscape (v.3.6.1) [68] to identify clusters of homologous BGCs. Singleton clusters were also aligned to the Canu+Pilon genome and individual Canu+Pilon antiSMASH BGCs using MUMmer v3.1 [60] to identify homologies that occurred at the nucleotide level but not at the protein level (e.g., due to high error rates that might confound gene prediction). Nucleotide-level BGC comparisons were also conducted using Mash (v.2.0) [59].

### Insertion sequence identification

Insertion sequences (ISs) were annotated in the *Fs* ARS-166-14 Canu, Canu+Pilon, SPAdes, and Unicycler assemblies using ISSaga2 [69]. Full and partial IS sequences were identified by comparing each assembly genome sequence to the ISfinder database. The default detection algorithm and parameters were used for all assemblies in this experiment, and both the total number of hits and those with > 70% amino acid similarity to ISs in the ISfinder database were recorded.

## Results

### Sequencing

We sequenced the genomes of nine bacterial strains using both Oxford Nanopore MinION and Illumina MiSeq technologies, together spanning a wide range of GC content (*Flavobacterium*: 31%; *Aeromonas*: 59–61%; *Pseudonocardia*: 74%). MinION sequencing coverage ranged from 40-135X and generated median read lengths of 1629–9665 bps (Table 2). Median MinION read lengths for *Ah* CA-13-1 and *Av* CIP107763^T^ were considerably shorter than for the other MinION libraries due to difficulties in extracting high molecular weight DNA from these strains. Illumina Nextera libraries were sequenced for all *Aeromonas* and *Flavobacterium* strains with coverage ranging from 30-169X (Table 3). Preliminary Nextera libraries were also constructed for the *Pseudonocardia* strains, but these were highly biased and generated extremely fragmented assemblies (1000s of contigs; Additional file 2: Figure S1). We therefore instead generated Illumina TruSeq PCR-free libraries for these strains, with coverage ranging from 71-246X (Table 3).

**Table 3:** Summary of Illumina sequencing

	Total Raw Reads	Total Bases (Mbps)	Coverage (fold)	Total Reads After Filtering
*Ps* JKS002128	6,120,982	1536	246	5,475,000
*Ps* JKS002072	1,766,572	443	71	1,638,060
*Ps* JKS002056	5,038,846	1265	203	4,736,206
*Av* JG3	1,488,761	372	79	942,391
*Av* CIP107763^T^	566,606	142	30	536,504
*Ah* CA-13-1	950,886	238	51	873,417
*Fs* ARS-166-14	2,164,975	541	169	890,703
*Fc* FC-100715-19	2,072,592	518	162	1,130,797
*Fc* FC-08-1215-1	1,145,425	286	89	987,428

### Genome assembly

**Fig. 1:**
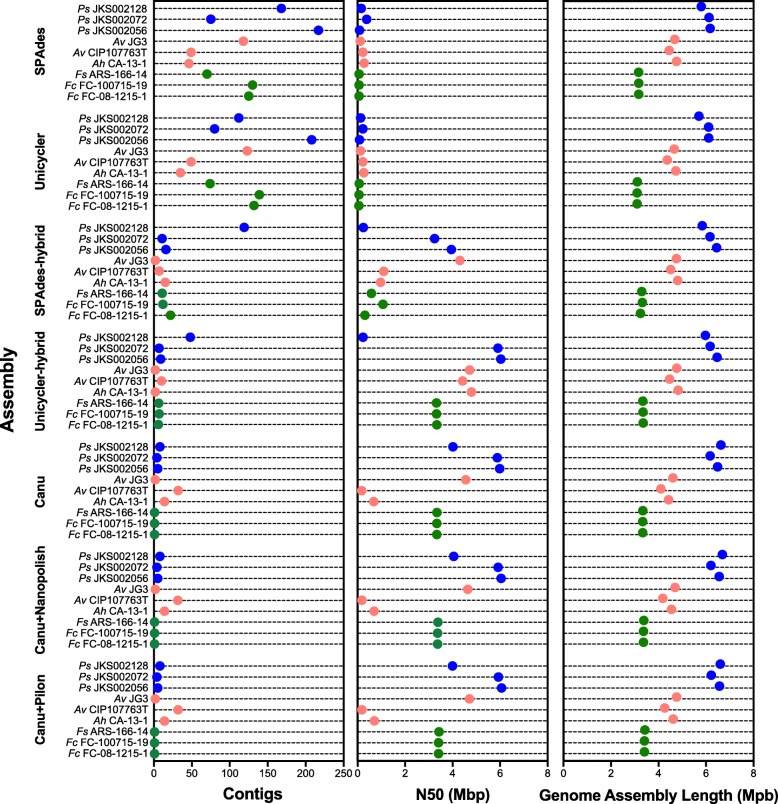
MinION reads improve assembly contiguity. The number of contigs (left), N50 (in Mbp, center), and assembly length (in Mbp, right) are shown for each of the MiSeq-based (SPAdes, Unicycler, SPAdes-hybrid, and Unicycler-hybrid) and MinION-based (Canu, Canu+Nanopolish, Canu+Pilon) genome assemblies. Results for *Pseudonocardia*, *Aeromonas*, and *Flavobacterium* are shown in blue, red, and green, respectively

Seven assemblies were generated for each strain, four based on MiSeq data either alone (SPAdes, Unicycler) or with MinION data to deconvolute the MiSeq assembly graph (SPAdes-hybrid, Unicycler-hybrid), and three based on MinION data either alone (Canu), polished using the same MinION data (Canu+Nanopolish), or polished using MiSeq data (Canu+Pilon). Both the SPAdes and Unicycler assemblies had the largest number of contigs out of all assemblies generated for each strain (Fig. 1). These assemblies also typically had the lowest N50 values compared to the other assemblies. *Ah* CA-13-1 and *Av* CIP107763^T^ were exceptions to this trend, likely due to their lower quality MinION libraries. The addition of MinION reads to deconvolute the SPAdes and Unicycler assembly graphs lowered the number of contigs and increased the N50 for all assemblies (Fig. 1). This highlights the ability of long MinION reads to resolve genomic repeats that otherwise stymied assembly of these genomes from short reads. Unicycler consistently outperformed SPAdes during hybrid assembly (the only exception being *Av* CIP107763^T^) but not when assembling MiSeq reads only.

Canu assemblies were more contiguous and had higher N50 values than all MiSeq-based assemblies, except for *Av* CIP107763^T^ Unicycler-hybrid and SPAdes-hybrid assemblies and the *Ah* CA-13-1 Unicycler-hybrid assembly (Fig. 1a, b). These two strains had lower quality MinION libraries (Table 2) that likely compromised the Canu assemblies, even if they were still more contiguous than the MiSeq-only SPAdes and Unicycler assemblies. Canu assemblies were used as the reference for polishing with either Nanopolish or Pilon, and so the number of contigs was the same for the Canu, Canu+Nanopolish, and Canu+Pilon assemblies (Fig. 1). The Canu assembly sizes were greater than those of any MiSeq-based assembly for all *Flavobacterium* and *Pseudonocardia* strains (up for ~ 14% for *Ps* JKS002128; Fig. 1), likely reflecting the MinION’s ability to overcome biases in the Illumina libraries for these genomes with low (31%) and high (74%) GC content, respectively (Additional file 2: Figure S2). This was not true for the *Aeromonas* assemblies, likely reflecting fewer biases in the Illumina libraries for these strains with more moderate GC content (59–61%). Taken together, these assemblies demonstrate that MinION sequencing improves assembly contiguity, especially where Illumina sequencing libraries are the most biased.

### Assembly accuracy

**Fig. 2:**
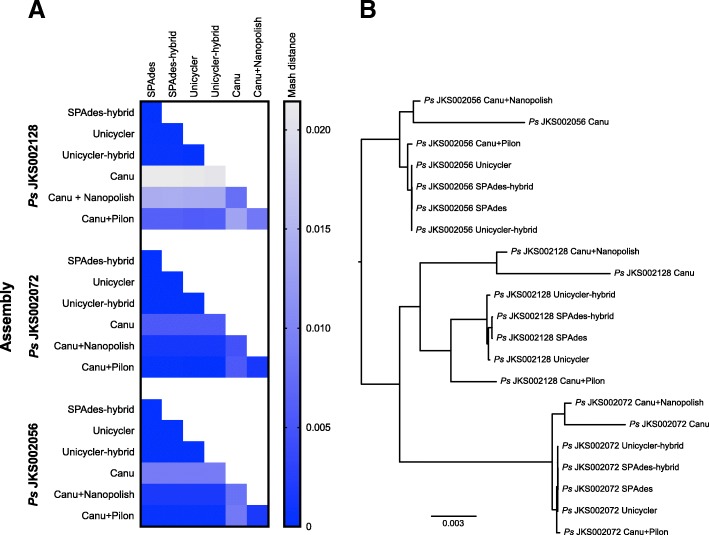
Comparison of *Pseudonocardia* assemblies generated during this study. (A): Heatmaps depicting Mash distances between the assemblies of each *Pseudonocardia* strain based on their shared k-mer content. Whiter colors indicate greater Mash distances between assemblies. (B): Mashtree analysis showing the relationships of all *Pseudonocardia* assemblies to each other, based on Mash distances. The scale bar represents a Mash distance of 0.003

Because we lacked high-quality reference genomes for our strains, we instead used several comparative analyses to assess the accuracy of our assemblies. We used Mash [59] to compare all of our *Pseudonocardia* assemblies to each other according to their shared k-mer content and to construct a distance-based phylogeny (Fig. 2). Canu assemblies were the least similar to the MiSeq-based assemblies, followed by the Canu+Nanopolish assemblies. This suggests that MinION data alone cannot produce accurate *Pseudonocardia* assemblies using current technologies. These data might alternatively be interpreted to mean that the MiSeq-based assemblies have lower accuracy compared to the Canu and Canu+Nanopolish assemblies, but we consider this unlikely based on previous research that argues against this interpretation [17, 27, 37, 38]. Canu+Pilon assemblies were more similar to the MiSeq-based assemblies, suggesting that polishing MinION-based assemblies with MiSeq reads is an effective strategy to generate microbial genome assemblies that are both accurate and contiguous. However, some divergence was observed between the Canu+Pilon and MiSeq-based genome assemblies. This was especially true for *Ps* JKS002128, which appeared to have the most biased MiSeq library in our study based on differences in the sizes of the MiSeq-based and MinION-based assemblies for this strain (Fig. 1). These differences are consistent with the existence of regions in the Canu assembly that lacked mapping MiSeq reads, leaving these regions uncorrected [70]. All genome assemblies for the same strain clustered together in the Mashtree analysis (Fig. 2), indicating that even the high error rates of the Canu and Canu+Nanopolish assemblies did not obscure strain-level phylogenetic differences.

**Fig. 3:**
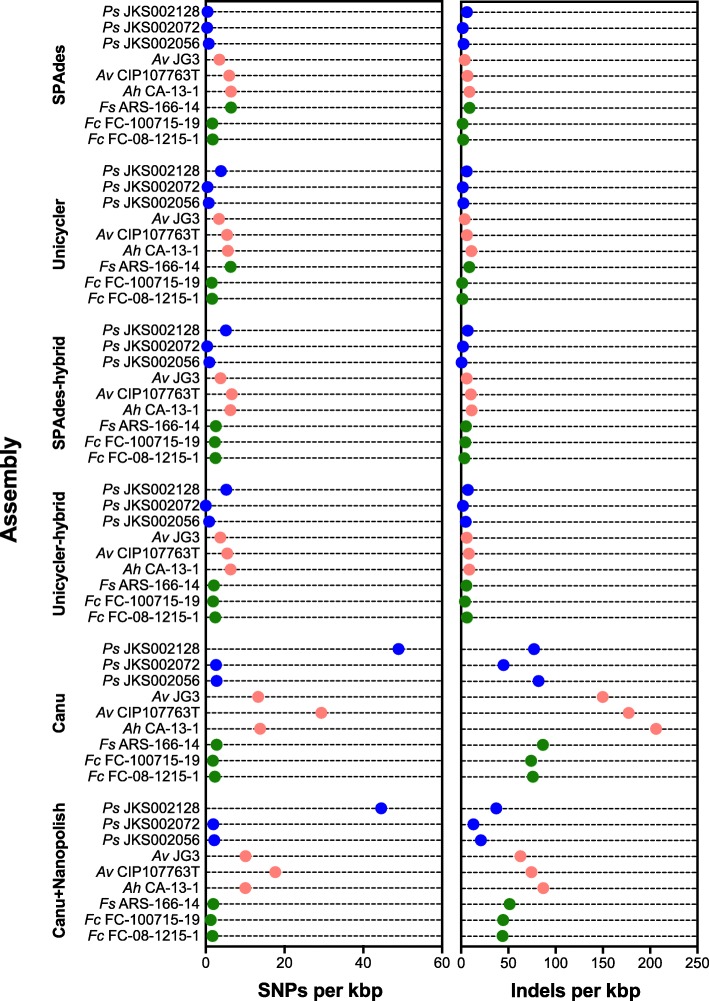
Quantification of insertion/deletions (indels, left) and single nucleotide polymorphisms (SNPs, right) in all strains sequenced during this study, as determined by aligning each assembly to the Canu+Pilon assembly for that strain as a reference

The Canu+Pilon assemblies were used as a reference against which to compare all other assemblies based on their higher contiguity and substantial accuracy. The high accuracy of MiSeq sequencing meant that all MiSeq-based assemblies had few SNPs and Indels relative to the Canu+Pilon assembly (Fig. 3). In contrast, the Canu assemblies had many more SNPs and indels relative to the Canu+Pilon assembly, especially for *Ps* JKS002056 (Fig. 3). Polishing these Canu assemblies using Nanopolish reduced the number of indels, and the number of SNPs to a lesser extent (Fig. 3). However, the numbers of SNPs and indels were still much higher than for the MiSeq-based assemblies. In all assemblies, both SNPs and indels were overrepresented at homopolymers relative to the distributions expected from the composition of each genome (Additional file 2: Figure S3). Similar error profiles have been reported previously [17, 26, 27, 37, 38].

**Fig. 4:**
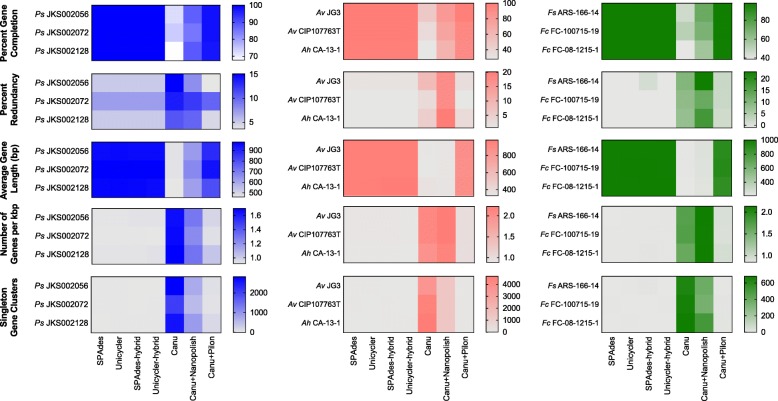
Anvi’o analysis of annotation quality. Strains are grouped by species with *Pseudonocardia* shown in blue, *Aeromonas* shown in red, and *Flavobacterium* shown in green. Each heatmap row corresponds to an individual strain and each column corresponds to a unique assembly method

To determine the effect of assembly method on gene annotation, we used Avni’o to compare annotated assemblies for each strain. Illumina-only and Illumina-hybrid annotations all had similar assembly metrics. Conversely, the number of genes predicted per kb, singleton gene clusters, and the percent of redundant genes (Fig. 4) were higher for the Canu and Canu+Nanopolish assemblies compared to the Illumina-only and Illumina-hybrid assemblies. These high numbers of genes per kb and low average gene lengths suggest that sequencing errors introduced stop codons that truncated genes and artificially increased gene counts in these assemblies. Many singleton gene clusters annotated in the Canu assemblies were not present in the Canu+Nanopolish assemblies and vice versa (Fig. 4, Additional file 2: Figure S4A-C). This suggests that although Nanopolish reduced the number of annotated singleton gene clusters, such polishing was not sufficient to correct all sequencing errors and may have itself introduced other annotation errors. Canu+Pilon annotations were most similar to annotations for Illumina-only and Illumina-hybrid assemblies. However, the average gene length and number of genes per kb for both *Ps* JKS002128 and *Ps* JKS002056 were smaller and larger, respectively, than for the Illumina-only and Illumina-hybrid annotations. This indicates that there is a tradeoff between assembly contiguity and annotation accuracy.

### MinION sequencing depth

**Fig. 5:**
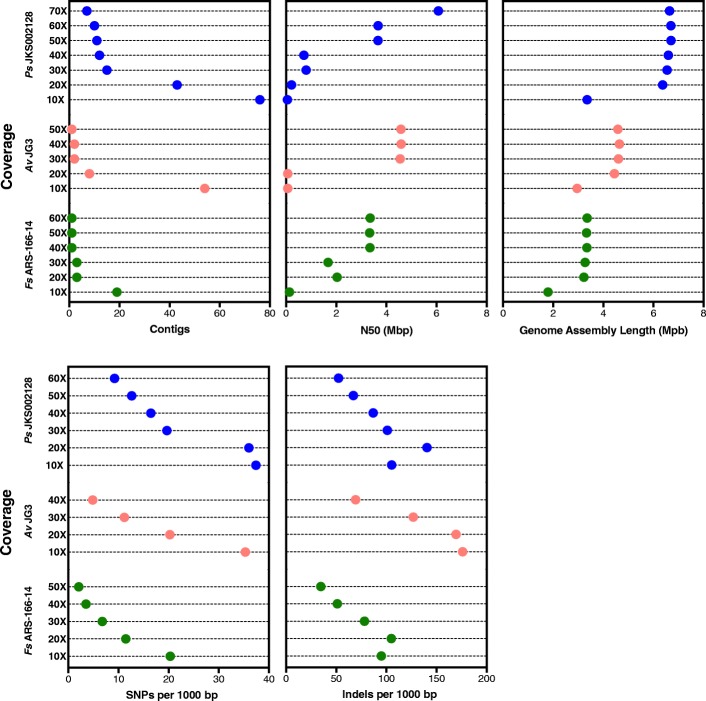
The effect of coverage on Canu genome assembly contiguity. The number of contigs (top left), N50 (in Mbp, top center), assembly length (in Mbp, top right), SNPs per 1000 bp (bottom right), and indels per 1000 bp (bottom left) are shown for subsets of the *Ps* JKS002128 (blue), *Av* JG3 (red), and *Fs* ARS-166-14 (green) MinION reads used in Fig. 1

Canu assemblies were performed using 5–7 different levels of coverage for strains *Av* JG3, *Fs* ARS-166-14, and *Ps* JKS002128. These assemblies suggest that the amount of coverage needed for a high-quality MinION-based genome assembly is relatively low, but also depends somewhat on the complexity of each genome. The contiguity of assemblies for strains *Av* JG3 and *Fs* ARS-166-14 did not improve substantially above 30X coverage, consistent with previous findings [71]. However, assemblies for strain *Ps* JKS002128 improved incrementally up to 70X coverage (Fig. 5), suggesting that higher coverage may be necessary for genomes with high GC content. The single 50X *Av* JG3 assembly also lacked a plasmid that was present in assemblies for the lower coverage datasets (data not known). Even though they were assembled into a few contigs, these assemblies were not error-free based on the different genome sizes and N50 values obtained for assemblies using different high-coverage datasets. The number of SNPs and indels detected also decreased with increased coverage. However, *Ps* JKS002128 required 20X more data to be comparable to *Av* JG3 and *Fs*-ARS-166-14, again indicating that high GC organisms may require additional coverage (Fig. 5). Researchers should therefore assess their goals for MinION sequencing before progressing with a run and consider stopping data collection at a certain threshold to conserve flow cells and to decrease sequencing time and cost.

### Biosynthetic gene cluster prediction

**Fig. 6:**
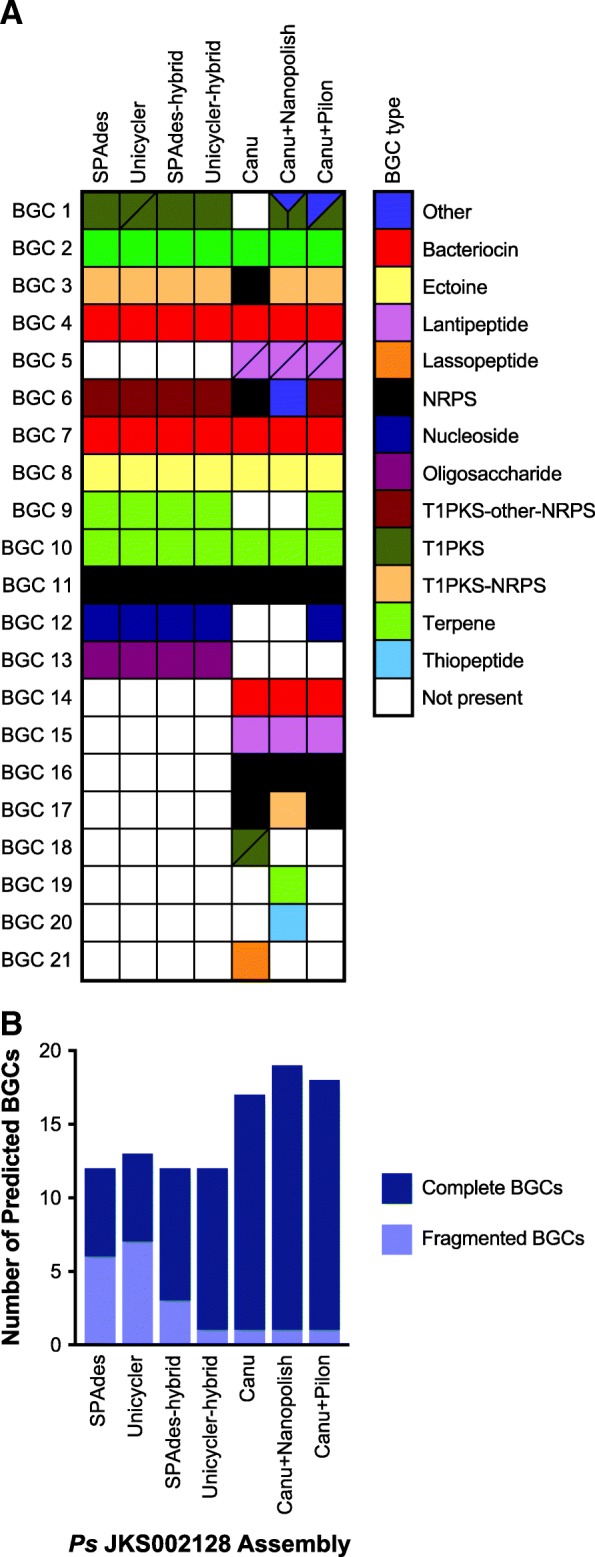
*Ps* JKS002128 genome assembly quality affects secondary metabolite biosynthetic gene cluster annotation. (A) Homologies between BGCs predicted for each *Ps* JKS002128 assembly, with each row representing a unique BGC in the *Ps* JKS002128 genome. Filled boxes indicate the BGCs found in each assembly, colored according to the type of secondary metabolite that it is predicted to encode. White boxes indicate BGCs that were not found in that assembly. Some BGCs occur on multiple contigs or are separated into multiple gene clusters on the same assembly, indicated by either two or three polygons within a single box. BGCs may still be fragmented even if represented by a single box. (B) The total number of complete and fragmented BGCs predicted in each *Ps* JKS002128 genome assembly

One expected benefit of high quality genome assemblies is that they will substantially improve the annotation of repetitive genomic regions relative to lower quality assemblies. To test this, we compared antiSMASH [65] secondary metabolite biosynthetic gene cluster (BGC) annotations for all of our *Ps* JKS002128 assemblies. Actinobacteria such as *Pseudonocardia* typically possess many BGCs, although they are often difficult to assemble correctly [16]. AntiSMASH consistently predicted 12 and 13 BGCs for the SPAdes and Unicycler assembles, respectively, and 12 BGCs for both the SPAdes-hybrid and Unicycler-hybrid assemblies (Fig. 6). The extra BGC in the Unicycler assembly is due to there being two separate fragments of BGC 1 annotated in this assembly. More BGCs were predicted for the Canu (17), Canu+Nanopolish (19), and Canu+Pilon (18) assemblies, including 4 BGCs that were found in at least two of these genomes but not in any of the MiSeq-based genomes (Fig. 6a). These BGCs may lie at particularly repetitive or bias-prone regions of the *Ps* JKS002128 genome such that they are omitted from MiSeq-based assemblies but present in MinION-based assemblies that are much less sensitive to these issues. Despite their greater contiguity, the Canu, Canu+Nanopolish, and Canu+Pilon assemblies lacked some combination of BGCs 1, 9, 12, and 13, all of which were found in all of the MiSeq-based assemblies (Fig. 6a). The Canu assembly lacked all 4 of these BGCs, the Canu+Nanopolish assembly lacked BGCs 9, 12, and 13, and the Canu+Pilon assembly only lacked BGC 13. These omissions are likely due to gene prediction errors that decreased the ability of antiSMASH to detect these BGCs (Additional file 2: Figure S5). Such errors may have also been responsible for the prediction of BGCs 18–21 solely in the Canu or Canu+Nanopolish assemblies (Fig. 6a), which are likely false positive annotations based on these BGCs only appearing in individual error-prone assemblies. MinION-based genome assemblies therefore substantially increase the sensitivity of BGC annotation, but require polishing to limit annotation errors.

Improved genome assembly also reduced the number of BGCs that were fragmented, i.e., that overlapped with a contig end (Fig. 6b). Approximately half of all BGCs in the SPAdes and Unicycler assemblies were fragmented, reflecting the inability of short-read Illumina data to resolve these repetitive genomic regions. The Unicycler hybrid, and to a lesser extent the SPAdes hybrid, assemblies had fewer fragmented BGCs, reflecting the increased contiguity of these assemblies. The Canu, Canu+Nanopolish, and Canu+Pilon assemblies all had very few fragmented BGCs. MinION-based genome assemblies therefore do not only increase the frequency of BGC detection, but also more completely assemble these BGCs and thus increase their value for genome-guided drug discovery. The Canu, Canu+Nanopolish, and Canu+Pilon assemblies did have several annotated gene clusters that were aggregated into a single BGC in other assemblies (Fig. 6a). Whether these represent single BGCs that were fragmented in the MinION-based assemblies or multiple BGCs that were located adjacent to each other on the *Ps* JKS002128 genome is difficult to predict computationally.

### Insertion sequence prediction

**Fig. 7:**
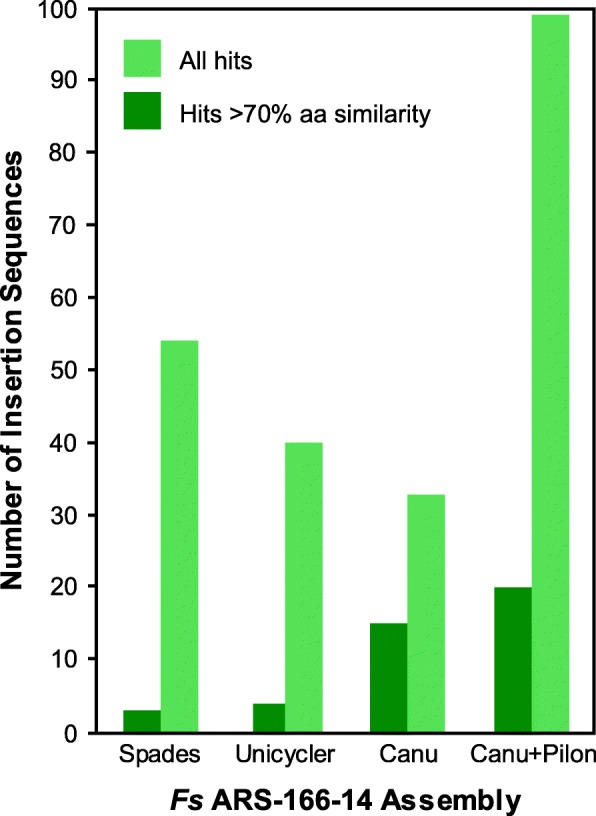
*Fs* ARS-166-14 genome assembly quality affects insertion sequences annotation. Both the total number of hits and hits with > 70% amino acid identity to insertion sequences in the ISfinder database are shown. The former likely includes false-positive annotations while the latter is more conservative

To further investigate the effect of genome assembly on the annotation of repetitive genomic regions, insertion sequences were predicted in the *Fs* ARS-166-14 Canu, Canu+Pilon, SPAdes, and Unicycler assemblies using ISSaga2 and the ISfinder database [69]. The total number of full or partial hits to the ISfinder database and the number of hits with amino acid sequence similarities > 70% are reported in Fig. 7. The Canu+Pilon assembly had 20 unique insertion sequences with 70% or greater sequence similarity to the ISfinder database, followed by the Canu assembly with 15, and then the Unicycler and SPAdes assemblies with 4 and 3, respectively. Interestingly, the Canu+Pilon assembly also had the greatest total number of hits, but these likely contain many false positive results that require further curation.

## Discussion

Single-molecule, long-read sequencing technologies such as the Oxford Nanopore MinION have strong potential to revolutionize the sequencing and de novo assembly of bacterial genomes. Existing short-read sequencing technologies frequently produce genome assemblies that are broken into 10s–100s of contigs, such as in our assemblies generated using only short-read MiSeq data (Fig. 1). Fragmented genome assemblies prevent accurate annotation of important genome features such as insertion sequences and secondary metabolite biosynthetic gene clusters (Figs. 6 and 7). Technological improvements are therefore necessary to fully understand and exploit these genomic features to cure disease and foster biotechnology.

One key reason for poor genome assembly is the inherently limited length of short-reads. By increasing the read length, long-read sequencing technologies such as the MinION disambiguate genomic repeats and generate fewer contig breaks (e.g., [38]). This was clearly evident from our SPAdes- and Unicycler-hybrid assemblies, where the long MinION reads were able to deconvolute the assembly graph produced from the MiSeq data and yielded fewer and longer contigs compared to the MiSeq-only assemblies (Fig. 1). Such improvements are likely to continue as MinION-compatible extraction methods for high-molecular weight DNA are refined.

However, the deconvolution of Illumina-based assemblies using long reads assumes that the entire genome is represented in the Illumina sequencing graph, which may not be true because of biases in short-read sequencing library preparation. As a result, some regions of the genome are sequenced to low coverage or excluded entirely, resulting in assembly fragmentation due to missing data. These problems include PCR biases against extreme %GC sequences [8–12] and biased insertion of transposases during library preparation [14]. Reflecting such biases, our initial *Pseudonocardia* sequencing experiments that used the Illumina Nextera library preparation method (which includes both transposases and PCR) produced genome assemblies with 1000 s of contigs (Additional file 2: Figure S1), compared to the 10 s–100 s of *Pseudonocardia* contigs produced using Illumina TruSeq PCR-free libraries (Fig. 1). Single-molecule sequencing methods such as the MinION avoid many of these biases by sequencing individual template DNA molecules without using PCR. This is reflected by the higher contiguity of our *Pseudonocardia* Canu genome assemblies compared to the SPAdes- and Unicycler-hybrid assemblies that used MinION reads to deconvolute the potentially biased Illumina assembly graphs (Fig. 1). All of our *Flavobacterium* and *Pseudonocardia* Canu assemblies are also larger than those based on Illumina reads, reflecting the inclusion of sequences that were missing from the Illumina sequencing libraries (Additional file 2: Figure S2). For *Pseudonocardia*, these differences were sometimes substantial (up to a ~ 14% increase in genome size). These results point to library preparation bias as a second source of error common to short-read sequencing that can be overcome by long-read, single-molecule sequencing technologies such as the MinION, in addition to the ability of MinION reads to span long genomic repeats.

Our results also highlight the importance of efficient high molecular weight DNA extraction methods for MinION sequencing. Of the 9 genomes that we sequenced during this study, the two with the lowest median read length (*Ah* CA-13-1 and *Av* CIP107763^T^) produced the least contiguous Canu assemblies (14 and 32 contigs, respectively). However, this is still more contiguous than the MiSeq-only SPAdes and Unicycler assemblies for these strains. MinION reads also improved these SPAdes and Unicycler assemblies when run in hybrid mode, demonstrating the utility of long reads even if DNA extraction remains suboptimal. There is a current need for reliable protocols to produce high molecular weight genomic DNA that is compatible with the MinION sequencer, and the Oxford Nanopore Voltrax and Ubik devices (https://nanoporetech.com/about-us/news/clive-g-brown-cto-plenary-london-calling) show strong potential to overcome these issues. The degree to which such devices are compatible with diverse cell wall chemistries remains to be validated.

Although most of our MinION-based assemblies were more contiguous than the MiSeq-based assemblies, they were less accurate. Assemblies generated using Canu contained a large number of SNPs and indels relative to our Illumina-based assemblies (Figs. 2 and 3) and lower quality gene annotations (Fig. 4). These differences were reduced by using Nanopolish to correct the Canu assembly using MinION reads, and even better results were obtained using Pilon to correct the Canu assembly using MiSeq reads (Figs. 2 and 3). However, differences still existed between these polished assemblies and the Illumina assemblies in some cases (most obviously for *Pseudonocardia* sp. JKS002128). Gene annotation in the Canu+Pilon assemblies also had a slightly lower quality relative to the Illumina-only and Illumina hybrid assemblies, likely due to frameshifts introduced during the assembly of error prone MinION reads (Fig. 4). Although it is possible that the MiSeq assemblies contained errors relative to the MinION assemblies, this would be inconsistent with previous work comparing MinION assemblies to high-quality reference genomes [17, 27, 37, 38]. Illumina reads are also unable to correct repetitive genome sequences that cannot be unambiguously mapped using short reads and so these regions will be uncorrected even in Canu+Pilon assemblies [70]. A tradeoff therefore exists between the higher contiguity of MinION-based assemblies relative to their higher number of SNP and indel errors. Minimizing such errors is a current technological focus of ONT (https://nanoporetech.com/about-us/news/clive-g-brown-cto-plenary-london-calling) and so this tradeoff may lessen in the near future.

The importance of these assembly trade-offs is highlighted by our analysis of repetitive genomic regions. For example, antiSMASH annotated ~ 1/3 more secondary metabolite biosynthetic gene clusters (BGC) in the MinION-based assemblies of *Pseudonocardia* sp. JKS002128 compared to the MiSeq-based assemblies (Fig. 6), confirming our previous observations that BGCs are poorly resolved by Illumina sequencing [16]. Similar results were obtained when annotating insertion sequences in *Flavobacterium* sp. *Fs* ARS-166-14, as expected due to the highly repetitive nature of these genomic regions (Fig. 6). The BGCs that were annotated in the Illumina-only assemblies were highly fragmented, highlighting the challenge of sequencing these complex genomic regions (Fig. 7) [16]. Interestingly, the genome assemblies that contained the highest number of SNP and indel errors (Fig. 3) contained several BGCs that were unique to those particular genomes (Fig. 6) and lacked several BGCs that were annotated in the MiSeq-based assemblies. These differences are likely due to the difficulty in accurately predicting gene structures in highly error-prone genomes due to gene truncation and misplaced start sites (Additional file 2: Figure S5). Indeed, our initial ClustCompare analysis to compare BGCs based on their protein sequences did not detect many true homologies between BGCs annotated in the Canu and Canu+Nanopolish assemblies to those annotated in assemblies that were generated or polished using MiSeq data due to the large number of misannotated gene structures in the Canu and Canu+Nanopolish assemblies (Additional file 2: Figure S5). These homologies only became clear using comparisons between nucleotide sequences. High numbers of SNP and indel errors can therefore prevent accurate genome annotation due to errors in gene structure prediction (Fig. 4). Several homologous BGCs were also annotated as belonging to different biosynthetic classes in different genomes (represented by the different colors in Fig. 6). Together, these analyses highlight the importance of contiguous and accurate genome assemblies for the prediction of repetitive elements such as BGCs and highlight the utility of MinION sequencing in this application, especially when polished using accurate Illumina reads.

In summary, our data highlights the ability of long-read, single-molecule MinION sequencing to overcome current limitations of short-read sequencing, particularly its inability to disambiguate repetitive genome regions and avoid biases introduced during library preparation. Overcoming these limitations greatly improves the annotation of many clinically- and biotechnologically-important genomic regions such as insertion sequences and BGCs (Figs. 6 and 7). However, SNP and indel errors remain problematic in de novo assemblies generated from MinION data (Fig. 3). This is likely to improve in the near future given the extensive research underway in this area. Because multiplexed genomes can currently be sequenced to sufficient coverage (40-50X; Fig. 5) on a single MinION or MiSeq flowcell, combining these data currently requires ~$100–$200 for the MinION and ~$150 for Illumina sequencing in reagent and consumable costs per genome. Combining these two data types is therefore an affordable means to dramatically increase the quality of any bacterial de novo genome assembly, regardless of their genome complexity or %GC content, and compares favorably to the cost of PacBio sequencing. Future technical advances will likely decrease these costs further, and we anticipate that highly contiguous and accurate de novo assembly of bacterial genomes will become standard in the field in the very near future.

## Conclusions

Short read genome assemblies struggle to disambiguate genomic repeats and are subject to technical biases. These biases are especially pronounced for genomes with extreme GC content. Our study validates a framework to overcome these biases by combining Oxford Nanopore MinION long reads with high-accuracy Illumina short reads. Genome assembly using long reads followed by polishing using short reads typically generated assemblies that were both contiguous and that facilitated accurate annotation. This includes improved annotation of complex genomic features such as secondary metabolite biosynthetic gene clusters and insertion sequences. An increase in frame shift errors was observed in some assemblies constructed from long reads, but anticipated improvements in base calling are likely to reduce these errors. These advances, coupled with the increasing cost-effectiveness of genome sequencing, will significantly improve studies of microbial evolution and genome-based drug discovery.

## Additional files

Additional file 1: Commands used for the analyses in this study. Note that the original file paths have been retained here, even though analyses were conducted on different servers. (DOCX 95 kb)

Additional file 2: **Figure S1.** Summary of Illumina Nextera-based assemblies for *Pseudonocardia* strains JKS002056, JKS002072, and JKS002128. **Figure S2.** BRIG analysis for *Pseudonocardia* strain JKS002128. The Canu+Pilon assembly was used as the reference strain. Each ring represents a different assembly type, Canu+Nanopolish (dark blue), Canu (pink), Unicycler Hybrid (green), Spades Hybrid (teal), Unicycler (orange), and Spades (purple). The inner rings describe GC content (black) and GC skew (purple/green). **Figure S3.** Analysis showing the ratio of SNPs and Indels present in homopolymeric regions ranging from 1 to 8 basepairs long for each *Ps* JKS002128, *Av* JG3, and *Fs* ARS-166-14 assembly relative to the Canu+Pilon assembly. Results for *Ps* JKS002128 did not detect SNPs present in homopolymeric regions 7 or 8 basepairs long in either the SPAdes, Unicycler, SPAdes-hybrid, or Unicycler-hybrid assemblies; these data points are therefore missing in this panel. **Figure S4.** Anvi’o Pangenome display for all strains. The Anvi’o (v.5.2) pangenome pipeline was used following the developer’s pipelines for importing Prokka gene annotations and for performing HMM analyses. Assembly methods are abbreviated as follows: S (SPAdes), U (Unicycler), SH (SPAdes-hybrid), UH (Unicycler-hybrid), P (Canu+Pilon), N (Canu+Nanopolish), and C (Canu). A. *Pseudonocardia* strains. B. *Aeromonas* strains. C. *Flavobacterium* strains. **Figure S5.** Alignments of Biosynthetic Gene Cluster family 6 (see Fig. 6a). Some Canu-based BGCs were shorter than the less error-prone BGCs annotated on the Illumina-based genomes. (DOCX 2669 kb)
