# Supplementary material for: Evaluation of strategies for the assembly of diverse bacterial genomes using MinION long-read sequencing
Source: BMC Genomics. 2019 Jan 9;20:23. doi: 10.1186/s12864-018-5381-7 (PMC6325685; doi:10.1186/s12864-018-5381-7)
Supplement: Supplementary file 1 — Commands used for the analyses in this study. Note that the original file paths have been retained here, even though analyses were conducted on different servers. (DOCX 95 kb) [file 12864_2018_5381_MOESM1_ESM.docx]

**Supplementary Material**

Commands used for the analyses in this study. Note that the original file paths have been retained here, even though analyses were conducted on different servers.

Commands for data in Figure 1

# MinION data preparation using porechop

porechop -i 1748_moreX_dec1_46x.fastq.gz -o porechopped/1748_moreX_dec1_46x_porechopped.fastq.gz --discard_middle > porechopped/1748_moreX_dec1_46x_porechop.log

porechop -i 2071_dec1_new_135x.fastq.gz -o porechopped/2071_dec1_new_135x_porechopped.fastq.gz --discard_middle > porechopped/2071_porechop.log

porechop -i 2345_dec1_new_90x.fastq.gz -o porechopped/2345_dec1_new_90x_porechopped.fastq.gz --discard_middle > porechopped/2345_porechop.log

porechop -i 2347_nov16_dec1_103x.fastq.gz -o porechopped/2347_nov16_dec1_103x_porechopped.fastq.gz --discard_middle > porechopped/2347_porechop.log

porechop -i 2348_nov16_new_74x.fastq.gz -o porechopped/2348_nov16_new_74x_porechopped.fastq.gz --discard_middle > porechopped/2348_porechop.log

porechop -i jg3_oct_nov_combined_67x.fastq.gz -o porechopped/jg3_oct_nov_combined_67x_porechopped.fastq.gz --discard_middle > porechopped/jg3_porechop.log

porechop -i JKH125_all_minION_reads.fastq.gz -o porechopped/JKH125_all_minION_reads_porechopped.fastq.gz --discard_middle > porechopped/JKH125_porechop.log

porechop -i JKH144_1D.fastq.gz -o porechopped/JKH144_1D_porechopped.fastq.gz --discard_middle > porechopped/JKH1448_porechop.log

porechop -i JKH158_long_reads.fastq.gz -o porechopped/JKH158_long_reads_porechopped.fastq.gz --discard_middle > porechopped/JKH158_porechop.log

# MinION data preparation using nanofilt

zcat porechopped/1748_moreX_dec1_46x_porechopped.fastq.gz | python3 ~/Tools/nanofilt-1.0.5/nanofilt/NanoFilt.py -q 9 -l 500 | gzip > nanofilt_porechopped/1748_moreX_dec1_46x_nanofilt_porechopped.fastq.gz

zcat porechopped/2071_dec1_new_135x_porechopped.fastq.gz | python3 ~/Tools/nanofilt-1.0.5/nanofilt/NanoFilt.py -q 9 -l 500 | gzip > nanofilt_porechopped/2071_dec1_new_135x_nanofilt_porechopped.fastq.gz

zcat porechopped/2345_dec1_new_90x_porechopped.fastq.gz | python3 ~/Tools/nanofilt-1.0.5/nanofilt/NanoFilt.py -q 9 -l 500 | gzip > nanofilt_porechopped/2345_dec1_new_90x_nanofilt_porechopped.fastq.gz

zcat porechopped/2347_nov16_dec1_103x_porechopped.fastq.gz | python3 ~/Tools/nanofilt-1.0.5/nanofilt/NanoFilt.py -q 9 -l 500 | gzip > nanofilt_porechopped/2347_nov16_dec1_103x_nanofilt_porechopped.fastq.gz

zcat porechopped/2348_nov16_new_74x_porechopped.fastq.gz | python3 ~/Tools/nanofilt-1.0.5/nanofilt/NanoFilt.py -q 9 -l 500 | gzip > nanofilt_porechopped/2348_nov16_new_74x_nanofilt_porechopped.fastq.gz

zcat porechopped/jg3_oct_nov_combined_67x_porechopped.fastq.gz | python3 ~/Tools/nanofilt-1.0.5/nanofilt/NanoFilt.py -q 9 -l 500 | gzip > nanofilt_porechopped/jg3_oct_nov_combined_67x_nanofilt_porechopped.fastq.gz

zcat porechopped/JKH125_all_minION_reads_porechopped.fastq.gz | python3 ~/Tools/nanofilt-1.0.5/nanofilt/NanoFilt.py -q 9 -l 500 | gzip > nanofilt_porechopped/JKH125_all_minION_reads_nanofilt_porechopped.fastq.gz

zcat porechopped/JKH144_1D_porechopped.fastq.gz | python3 ~/Tools/nanofilt-1.0.5/nanofilt/NanoFilt.py -q 9 -l 500 | gzip > nanofilt_porechopped/JKH144_1D_nanofilt_porechopped.fastq.gz

zcat porechopped/JKH158_long_reads_porechopped.fastq.gz | python3 ~/Tools/nanofilt-1.0.5/nanofilt/NanoFilt.py -q 9 -l 500 | gzip > nanofilt_porechopped/JKH158_long_reads_nanofilt_porechopped.fastq.gz

# Unicycler Illumina assemblies

unicycler -1 jg3_new_trim_R1_paired.fastq -2 jg3_new_trim_R2_paired.fastq -o unicycler_RE_jg3/

unicycler -1 2348_new_trim_R1_paired.fastq -2 2348_new_trim_R2_paired.fastq -o unicycler_RE_2348/

unicycler -1 AH1_S1_L001_R1_001_tr.fastq -2 AH1_S1_L001_R2_001_tr.fastq -o unicycler_RE_1748/

unicycler -1 CIP107763_S35_L001_R1_001_tr.fastq -2 CIP107763_S35_L001_R2_001_tr.fastq -o unicycler_2071/

unicycler -1 /home/CAM/lbeka/minION/illumina_reads/2345_ARS-166-14_NextGenome_3_nextera/NextGenome-3_S111_L001_R1_001_tr.fastq.gz -2 /home/CAM/lbeka/minION/illumina_reads/2345_ARS-166-14_NextGenome_3_nextera/NextGenome-3_S111_L001_R2_001_tr.fastq.gz -o unicycler_2345

unicycler -1 2347_NextGenome6_R1_tr.fastq.gz -2 2347_NextGenome6_R2_tr.fastq.gz -o unicycler_2347

unicycler -1 JKH125_TruSeq_output_R1_paired.fastq -2 JKH125_TruSeq_output_R2_paired.fastq -o JKH125_Unicycler_TruSeq_1_17_18

unicycler -1 JKH144_TruSeq_output_R1_paired.fastq -2 JKH144_TruSeq_output_R2_paired.fastq -o JKH144_Unicycler_TruSeq_1_17_18

unicycler -1 JKH158_TruSeq_output_R1_paired.fastq -2 JKH158_TruSeq_output_R2_paired.fastq -o JKH158_Unicycler_TruSeq_1_17_18

# SPAdes Illumina assemblies

spades.py --pe1-1 jg3_new_trim_R1_paired.fastq --pe1-2 jg3_new_trim_R2_paired.fastq -o spades_redo_jg3

spades.py --pe1-1 AH1_S1_L001_R1_001_tr.fastq --pe1-2 AH1_S1_L001_R2_001_tr.fastq -o spades_redo_1748

spades.py --pe1-1 2348_new_trim_R1_paired.fastq --pe1-2 2348_new_trim_R2_paired.fastq -o spades_redo_2348

spades.py --pe1-1 CIP107763_S35_L001_R1_001_tr.fastq --pe1-2 CIP107763_S35_L001_R2_001_tr.fastq -o spades_2071

spades.py --pe1-1 /home/CAM/lbeka/minION/illumina_reads/2345_ARS-166-14_NextGenome_3_nextera/NextGenome-3_S111_L001_R1_001_tr.fastq.gz --pe1-2 /home/CAM/lbeka/minION/illumina_reads/2345_ARS-166-14_NextGenome_3_nextera/NextGenome-3_S111_L001_R2_001_tr.fastq.gz -t 4 -o 2345_spades

spades.py --pe1-1 2347_NextGenome6_R1_tr.fastq.gz --pe1-2 2347_NextGenome6_R2_tr.fastq.gz -t 4 -o 2347_spades

spades.py --pe1-1 JKH125_TruSeq_output_R1_paired.fastq --pe1-2 JKH125_TruSeq_output_R2_paired.fastq -o JKH125_SPAdes_TruSeq_1_17_18

spades.py --pe1-1 JKH144_TruSeq_output_R1_paired.fastq --pe1-2 JKH144_TruSeq_output_R2_paired.fastq -o JKH144_SPAdes_TruSeq_1_17_18

spades.py --pe1-1 JKH158_TruSeq_output_R1_paired.fastq --pe1-2 JKH158_TruSeq_output_R2_paired.fastq -o JKH158_SPAdes_TruSeq_1_17_18

# Unicycler hybrid assemblies

unicycler -1 JKH125_TruSeq_output_R1_paired_6_20.fastq.gz -2 JKH125_TruSeq_output_R2_paired_6_20.fastq.gz -l JKH125_all_minION_reads_nanofilt_porechopped.fastq.gz -o /home/CAM/ttesterman/Lidia/ --verbosity 2

unicycler -1 /home/CAM/ttesterman/Lidia/jkh144/JKH144_TruSeq_PCR_Free_R1_concat.fastq -2 /home/CAM/ttesterman/Lidia/jkh144/JKH144_TruSeq_PCR_Free_R2_concat.fastq -l /home/CAM/ttesterman/Lidia/jkh144/JKH144_1D_nanofilt_porechopped.fastq.gz -o /home/CAM/ttesterman/Lidia/jkh144/ --verbosity 2

unicycler -1 /home/CAM/ttesterman/Lidia/jkh158/try2/JKH158_TruSeq_output_R1_paired.fastq.gz -2 /home/CAM/ttesterman/Lidia/jkh158/try2/JKH158_TruSeq_output_R2_paired.fastq.gz -l /home/CAM/ttesterman/Lidia/jkh158/try2/JKH158_long_reads_nanofilt_porechopped.fastq.gz -o /home/CAM/ttesterman/Lidia/jkh125/try2/final_unicyclHY_jkh158 --verbosity 2 --threads 24

unicycler -1 AH1_S1_L001_R1_001_tr.fastq.gz -2 AH1_S1_L001_R2_001_tr.fastq.gz -l /home/CAM/lbeka/minION/new_analysis/spades_hybrid/1748/1748_moreX_dec1_46x_nanofilt_porechopped.fastq.gz -o uniHY_1748 -t 10 --verbosity 2

unicycler -1 CIP107763_S35_L001_R1_001_tr.fastq.gz -2 CIP107763_S35_L001_R2_001_tr.fastq.gz -l 2071_dec1_new_135x_nanofilt_porechopped.fastq.gz -o 2071_uniHY -t 4 --verbosity 2

unicycler -1 /home/CAM/lbeka/minION/illumina_reads/2345_ARS-166-14_NextGenome_3_nextera/NextGenome-3_S111_L001_R1_001_tr.fastq.gz -2 /home/CAM/lbeka/minION/illumina_reads/2345_ARS-166-14_NextGenome_3_nextera/NextGenome-3_S111_L001_R2_001_tr.fastq.gz -l /home/CAM/lbeka/minION/new_analysis/spades_hybrid/2345/2345_dec1_new_90x_nanofilt_porechopped.fastq.gz -o unicycler_hybrid_2345 --threads 4 --verbosity 2

unicycler -1 2347_NextGenome6_R1_tr.fastq.gz -2 2347_NextGenome6_R2_tr.fastq.gz -l 2347_nov16_dec1_103x_nanofilt_porechopped.fastq.gz -o unicycler_hybrid_assembly_2347 -t 4 --verbosity 2

unicycler -1 2348_new_trim_R1_paired.fastq.gz -2 2348_new_trim_R2_paired.fastq.gz -l /home/CAM/lbeka/minION/new_analysis/spades_hybrid/2348/2348_nov16_new_74x_nanofilt_porechopped.fastq.gz -o /home/CAM/lbeka/minION/new_analysis/unicycler_hybrid/2348/unicycler_hybrid_2348 -t 10 --verbosity 2

unicycler -1 jg3_new_trim_R1_paired.fastq.gz -2 jg3_new_trim_R2_paired.fastq.gz -l /home/CAM/lbeka/minION/new_analysis/spades_hybrid/jg3/jg3_oct_nov_combined_67x_nanofilt_porechopped.fastq.gz -o /home/CAM/lbeka/minION/new_analysis/unicycler_hybrid/jg3/uniHY -t 10 --verbosity 2

# SPAdes hybrid assemblies

spades.py -1 /home/CAM/ttesterman/Lidia/jkh125_unicycler/JKH125_TruSeq_output_R1_paired_6_20.fastq.gz -2 /home/CAM/ttesterman/Lidia/jkh125_unicycler/JKH125_TruSeq_output_R2_paired_6_20.fastq.gz --nanopore /home/CAM/ttesterman/Lidia/jkh125_unicycler/JKH125_all_minION_reads_nanofilt_porechopped.fastq.gz -t 4 -o /home/CAM/ttesterman/Lidia/SPADES_hybrid/jkh125/

spades.py -1 /home/CAM/ttesterman/Lidia/jkh144/JKH144_TruSeq_PCR_Free_R1_concat.fastq -2 /home/CAM/ttesterman/Lidia/jkh144/JKH144_TruSeq_PCR_Free_R2_concat.fastq -–nanopore /home/CAM/ttesterman/Lidia/jkh144/JKH144_1D_nanofilt_porechopped.fastq.gz -t 4 -o /home/CAM/ttesterman/Lidia/SPADES_hybrid/jkh144/

spades.py -1 /home/CAM/ttesterman/Lidia/jkh158/try2/JKH158_TruSeq_output_R1_paired.fastq.gz -2 /home/CAM/ttesterman/Lidia/jkh158/try2/JKH158_TruSeq_output_R2_paired.fastq.gz --nanopore /home/CAM/ttesterman/Lidia/jkh158/try2/JKH158_long_reads_nanofilt_porechopped.fastq.gz -t 10 -o /home/CAM/ttesterman/Lidia/SPADES_hybrid/jkh158/

spades.py -1 /home/CAM/lbeka/minION/new_analysis/unicycler_hybrid/1748/AH1_S1_L001_R1_001_tr.fastq.gz -2 /home/CAM/lbeka/minION/new_analysis/unicycler_hybrid/1748/AH1_S1_L001_R2_001_tr.fastq.gz --nanopore 1748_moreX_dec1_46x_nanofilt_porechopped.fastq.gz -t 20 -o 1748_spadesHY

spades.py -1 /home/CAM/lbeka/minION/new_analysis/unicycler_hybrid/2071/CIP107763_S35_L001_R1_001_tr.fastq.gz -2 home/CAM/lbeka/minION/new_analysis/unicycler_hybrid/2071/CIP107763_S35_L001_R2_001_tr.fastq.gz --nanopore 2071_dec1_new_135x_nanofilt_porechopped.fastq.gz -t 20 -o 2071_spadesHY

spades.py -1 /home/CAM/lbeka/minION/illumina_reads/2345_ARS-166-14_NextGenome_3_nextera/NextGenome-3_S111_L001_R1_001_tr.fastq.gz -2 /home/CAM/lbeka/minION/illumina_reads/2345_ARS-166-14_NextGenome_3_nextera/NextGenome-3_S111_L001_R2_001_tr.fastq.gz --nanopore /home/CAM/lbeka/minION/new_analysis/spades_hybrid/2345/NEW/2345_dec1_new_90x_nanofilt_porechopped.fastq.gz -t 20 -o 2345_spadesHY

spades.py -1 /home/CAM/lbeka/minION/new_analysis/unicycler_hybrid/2347/NEW/2347_NextGenome6_R1_tr.fastq.gz -2 /home/CAM/lbeka/minION/new_analysis/unicycler_hybrid/2347/NEW/2347_NextGenome6_R2_tr.fastq.gz --nanopore 2347_nov16_dec1_103x_nanofilt_porechopped.fastq.gz -o 2347_spadesHY -t 20

spades.py -1 /home/CAM/lbeka/minION/new_analysis/unicycler_hybrid/2348/2348_new_trim_R1_paired.fastq.gz -2 /home/CAM/lbeka/minION/new_analysis/unicycler_hybrid/2348/2348_new_trim_R2_paired.fastq.gz --nanopore 2348_nov16_new_74x_nanofilt_porechopped.fastq.gz -o 2348_spadyesHY -t 10

spades.py -1 jg3_new_trim_R1_paired.fastq -2 jg3_new_trim_R2_paired.fastq --nanopore jg3_oct_nov_combined_67x_nanofilt_porechopped.fastq -o jg3_spadesHY -t 10

# Canu assemblies

~/Tools/canu-1.5/Linux-amd64/bin/canu -p 1748_moreX_dec1_46x_nanofilt_porechopped -d canu/1748_moreX_dec1_46x_nanofilt_porechopped -genomeSize=4.7m -nanopore-raw nanofilt_porechopped/1748_moreX_dec1_46x_nanofilt_porechopped.fastq.gz gnuplotTested=true stopOnReadQuality=false > canu/1748_canu.log

~/Tools/canu-1.5/Linux-amd64/bin/canu -p 2071_dec1_new_135x_nanofilt_porechopped -d canu/2071_dec1_new_135x_nanofilt_porechopped -genomeSize=4.7m -nanopore-raw nanofilt_porechopped/2071_dec1_new_135x_nanofilt_porechopped.fastq.gz gnuplotTested=true stopOnReadQuality=false > canu/2071_canu.log

~/Tools/canu-1.5/Linux-amd64/bin/canu -p 2345_dec1_new_90x_nanofilt_porechopped -d canu/2345_dec1_new_90x_nanofilt_porechopped -genomeSize=3.2m -nanopore-raw nanofilt_porechopped/2345_dec1_new_90x_nanofilt_porechopped.fastq.gz gnuplotTested=true stopOnReadQuality=false > canu/2345_canu.log

~/Tools/canu-1.5/Linux-amd64/bin/canu -p 2347_nov16_dec1_103x_nanofilt_porechopped -d canu/2347_nov16_dec1_103x_nanofilt_porechopped -genomeSize=3.2m -nanopore-raw nanofilt_porechopped/2347_nov16_dec1_103x_nanofilt_porechopped.fastq.gz gnuplotTested=true stopOnReadQuality=false > canu/2347_canu.log

~/Tools/canu-1.5/Linux-amd64/bin/canu -p 2348_nov16_new_74x_nanofilt_porechopped -d canu/2348_nov16_new_74x_nanofilt_porechopped -genomeSize=3.2m -nanopore-raw nanofilt_porechopped/2348_nov16_new_74x_nanofilt_porechopped.fastq.gz gnuplotTested=true stopOnReadQuality=false > canu/2348_canu.log

~/Tools/canu-1.5/Linux-amd64/bin/canu -p jg3_oct_nov_combined_67x_nanofilt_porechopped -d canu/jg3_oct_nov_combined_67x_nanofilt_porechopped -genomeSize=4.7m -nanopore-raw nanofilt_porechopped/jg3_oct_nov_combined_67x_nanofilt_porechopped.fastq.gz gnuplotTested=true stopOnReadQuality=false > canu/jg3_canu.log

~/Tools/canu-1.5/Linux-amd64/bin/canu -p JKH125_all_minION_reads_nanofilt_porechopped -d canu/JKH125_all_minION_reads_nanofilt_porechopped -genomeSize=6.5m -nanopore-raw nanofilt_porechopped/JKH125_all_minION_reads_nanofilt_porechopped.fastq.gz gnuplotTested=true stopOnReadQuality=false > canu/JKH125_canu.log

~/Tools/canu-1.5/Linux-amd64/bin/canu -p JKH144_1D_nanofilt_porechopped -d canu/JKH144_1D_nanofilt_porechopped -genomeSize=6.5m -nanopore-raw nanofilt_porechopped/JKH144_1D_nanofilt_porechopped.fastq.gz gnuplotTested=true stopOnReadQuality=false > canu/JKH144_canu.log

~/Tools/canu-1.5/Linux-amd64/bin/canu -p JKH158_long_reads_nanofilt_porechopped -d canu/JKH158_long_reads_nanofilt_porechopped -genomeSize=6.5m -nanopore-raw nanofilt_porechopped/JKH158_long_reads_nanofilt_porechopped.fastq.gz gnuplotTested=true stopOnReadQuality=false > canu/JKH158_canu.log

# Canu + nanopolish assemblies

Indexing canu assembly

bwa index JKH125_all_minION_reads_nanofilt_porechopped.contigs.fasta

bwa index JKH144_1D_nanofilt_porechopped.contigs.fasta

bwa index JKH158_long_reads_nanofilt_porechopped.contigs.fasta

bwa index 2345_dec1_new_90x_nanofilt_porechopped.contigs.fasta

bwa index 1748_moreX_dec1_46x_nanofilt_porechopped.contigs.fasta

bwa index jg3_oct_nov_combined_67x_nanofilt_porechopped.contigs.fasta

bwa index 2071_canu.fasta

bwa index 2348_canu.fasta

bwa index 2347_nov16_dec1_103x_nanofilt_porechopped.contigs.fasta

Extracting base-called fast5’s

nanopolish extract --type template /home/CAM/sgoldstein/minION_fast5_nanopolish/JKH125_metrichore_pass > /home/CAM/sgoldstein/canus/jkh125/jkh125_nanoX_reads.fasta

nanopolish extract --type template /home/CAM/sgoldstein/minION_fast5_nanopolish/JKH158_Albacore_basecalled_workspace > /home/CAM/sgoldstein/canus/jkh158/jkh158_nanoX_reads.fasta

nanopolish extract --type template /home/CAM/sgoldstein/minION_fast5_nanopolish/JKH144_Albacore_basecalled_workspace > /home/CAM/sgoldstein/canus/jkh144/jkh144_nanoX_reads.fasta

nanopolish extract --type template /home/CAM/lbeka/minION/old_analysis/nanopolish/albacore_output_for_nanopolish/2345_moreX_bc7_dec1 > /home/CAM/lbeka/minION/new_analysis/nanopolish/2345_NEW_nanoX_reads.fasta

nanopolish extract --type template /home/CAM/lbeka/minION/old_analysis/nanopolish/march_nanoextract/jg3_bc3_nov1 > /home/CAM/lbeka/minION/new_analysis/nanopolish/jg3/jg3_nanoX_New_nov1_reads.fasta

nanopolish extract --type template /home/CAM/lbeka/minION/epi2me_pass_batch_1508684229561 > /home/CAM/lbeka/minION/new_analysis/nanopolish/jg3/jg3_nanoX_NEW_epi2me_reads.fasta

cat jg3_nanoX_New_nov1_reads.fasta jg3_nanoX_NEW_epi2me_reads.fasta > jg3_combined_nanoX_reads.fasta

nanopolish extract --type template /home/CAM/lbeka/minION/old_analysis/nanopolish/march_nanoextract/2347_moreX_bc8_dec1 > /home/CAM/lbeka/minION/new_analysis/nanopolish/2347/2347_nanoX_NEW_dec_reads.fasta

nanopolish extract --type template /home/CAM/lbeka/minION/old_analysis/nanopolish/march_nanoextract/2347_bc4_nov17 > /home/CAM/lbeka/minION/new_analysis/nanopolish/2347/2347_nanoX_NEW_nov17_reads.fasta

cat 2347_nanoX_NEW_dec_reads.fasta 2347_nanoX_NEW_nov17_reads.fasta > 2347_combined_NEW_nanoX_reads.fasta

nanopolish extract --type template /home/CAM/lbeka/minION/old_analysis/nanopolish/march_nanoextract/1748_moreX_bc1_dec1 > /home/CAM/lbeka/minION/new_analysis/nanopolish/1748/1748_nanoX_NEW_dec46Xonly_reads.fasta

nanopolish extract --type template /home/CAM/lbeka/minION/old_analysis/nanopolish/march_nanoextract/2071_moreX_bc2_dec1 > /home/CAM/lbeka/minION/new_analysis/nanopolish/2071/2071_nanoX_NEW_dec135Xonly_reads.fasta

nanopolish extract --type template /home/CAM/lbeka/minION/old_analysis/nanopolish/march_nanoextract/2348_bc6_nov17/ > /home/CAM/lbeka/minION/new_analysis/nanopolish/2348/2348_THIS_nanoX.fasta

Aligning base-called reads to draft assembly

bwa mem -x ont2d /home/CAM/sgoldstein/canus/jkh125/JKH125_all_minION_reads_nanofilt_porechopped.contigs.fasta /home/CAM/sgoldstein/canus/jkh125/jkh125_nanoX_reads.fasta | samtools sort -o /home/CAM/sgoldstein/canus/jkh125/jkh125_reads.sorted.bam -T jkh125_reads.tmp

samtools index /home/CAM/sgoldstein/canus/jkh125/jkh125_reads.sorted.bam

bwa mem -x ont2d /home/CAM/sgoldstein/canus/jkh144/JKH144_1D_nanofilt_porechopped.contigs.fasta /home/CAM/sgoldstein/canus/jkh144/jkh144_nanoX_reads.fasta | samtools sort -o /home/CAM/sgoldstein/canus/jkh144/jkh144_reads.sorted.bam -T jkh144_reads.tmp

samtools index /home/CAM/sgoldstein/canus/jkh144/jkh144_reads.sorted.bam

bwa mem -x ont2d /home/CAM/sgoldstein/canus/jkh158/JKH158_long_reads_nanofilt_porechopped.contigs.fasta /home/CAM/sgoldstein/canus/jkh158/jkh158_nanoX_reads.fasta | samtools sort -o /home/CAM/sgoldstein/canus/jkh158/jkh158_reads.sorted.bam -T jkh158_reads.tmp

samtools index /home/CAM/sgoldstein/canus/jkh158/jkh158_reads.sorted.bam

bwa mem -x ont2d /home/CAM/lbeka/minION/new_analysis/nanopolish/2345_dec1_new_90x_nanofilt_porechopped.contigs.fasta /home/CAM/lbeka/minION/new_analysis/nanopolish/2345_NEW_nanoX_reads.fasta | samtools sort -o /home/CAM/lbeka/minION/new_analysis/nanopolish/2345_reads.sorted.bam -T /home/CAM/lbeka/minION/new_analysis/nanopolish/2345_reads.tmp --threads 20

samtools index /home/CAM/lbeka/minION/new_analysis/nanopolish/2345_reads.sorted.bam

bwa mem -x ont2d /home/CAM/lbeka/minION/new_analysis/nanopolish/2347/2347_nov16_dec1_103x_nanofilt_porechopped.contigs.fasta /home/CAM/lbeka/minION/new_analysis/nanopolish/2347/2347_combined_NEW_nanoX_reads.fasta | samtools sort -o /home/CAM/lbeka/minION/new_analysis/nanopolish/2347/2347_reads.sorted.bam -T /home/CAM/lbeka/minION/new_analysis/nanopolish/2347/2347_reads.tmp --threads 20

samtools index /home/CAM/lbeka/minION/new_analysis/nanopolish/2347/2347_reads.sorted.bam

bwa mem -x ont2d /home/CAM/lbeka/minION/new_analysis/nanopolish/1748/1748_moreX_dec1_46x_nanofilt_porechopped.contigs.fasta /home/CAM/lbeka/minION/new_analysis/nanopolish/1748/1748_nanoX_NEW_dec46Xonly_reads.fasta | samtools sort -o /home/CAM/lbeka/minION/new_analysis/nanopolish/1748/1748_reads.sorted.bam -T /home/CAM/lbeka/minION/new_analysis/nanopolish/1748/1748_reads.tmp --threads 20

samtools index /home/CAM/lbeka/minION/new_analysis/nanopolish/1748/1748_reads.sorted.bam

bwa mem -x ont2d /home/CAM/lbeka/minION/new_analysis/nanopolish/jg3/jg3_oct_nov_combined_67x_nanofilt_porechopped.contigs.fasta /home/CAM/lbeka/minION/new_analysis/nanopolish/jg3/jg3_combined_nanoX_reads.fasta | samtools sort -o /home/CAM/lbeka/minION/new_analysis/nanopolish/jg3/jg3_reads.sorted.bam -T /home/CAM/lbeka/minION/new_analysis/nanopolish/jg3/jg3_reads.tmp --threads 24

samtools index /home/CAM/lbeka/minION/new_analysis/nanopolish/jg3/jg3_reads.sorted.bam

bwa mem -x ont2d 2348_canu.fasta 2348_THIS_nanoX.fasta | samtools sort -o 2348_reads.sorted.bam -T 2348_reads.tmp --threads 10

samtools index 2348_reads.sorted.bam

bwa mem -x ont2d 2071_canu.fasta 2071_nanoX_NEW_dec135Xonly_reads.fasta | samtools sort -o 2071_reads.sorted.bam -T 2071_reads.tmp --threads 10

samtools index 2071_reads.sorted.bam

Splitting draft genome into 50kb segments & consensus algorithm

python /home/CAM/sgoldstein/canus/nanopolish_makerange.py /home/CAM/sgoldstein/canus/jkh144/JKH144_1D_nanofilt_porechopped.contigs.fasta | parallel --results jkh144_nanopolish.results -P 20 \ nanopolish variants --consensus polished.{1}.fa -w {1} -r /home/CAM/sgoldstein/canus/jkh144/jkh144_nanoX_reads.fasta -b /home/CAM/sgoldstein/canus/jkh144/jkh144_reads.sorted.bam -g /home/CAM/sgoldstein/canus/jkh144/JKH144_1D_nanofilt_porechopped.contigs.fasta --min-candidate-frequency 0.1

python /home/CAM/sgoldstein/canus/nanopolish_makerange.py /home/CAM/sgoldstein/canus/jkh125/JKH125_all_minION_reads_nanofilt_porechopped.contigs.fasta | parallel --results jkh125_nanopolish.results -P 20 \ nanopolish variants --consensus polished.{1}.fa -w {1} -r /home/CAM/sgoldstein/canus/jkh125/jkh125_nanoX_reads.fasta -b /home/CAM/sgoldstein/canus/jkh125/jkh125_reads.sorted.bam -g /home/CAM/sgoldstein/canus/jkh125/JKH125_all_minION_reads_nanofilt_porechopped.contigs.fasta --min-candidate-frequency 0.1

python /home/CAM/sgoldstein/canus/nanopolish_makerange.py /home/CAM/sgoldstein/canus/jkh158/JKH158_long_reads_nanofilt_porechopped.contigs.fasta | parallel --results jkh158_nanopolish.results -P 20 \ nanopolish variants --consensus polished.{1}.fa -w {1} -r /home/CAM/sgoldstein/canus/jkh158/jkh158_nanoX_reads.fasta -b /home/CAM/sgoldstein/canus/jkh158/jkh158_reads.sorted.bam -g /home/CAM/sgoldstein/canus/jkh158/JKH158_long_reads_nanofilt_porechopped.contigs.fasta --min-candidate-frequency 0.1

python /home/CAM/lbeka/minION/new_analysis/nanopolish/scripts/nanopolish_makerange.py /home/CAM/lbeka/minION/new_analysis/nanopolish/2345_dec1_new_90x_nanofilt_porechopped.contigs.fasta | parallel --results 2345_nanopolish.results -P 20 \ nanopolish variants --consensus polished.{1}.fa -w {1} -r /home/CAM/lbeka/minION/new_analysis/nanopolish/2345_NEW_nanoX_reads.fasta -b /home/CAM/lbeka/minION/new_analysis/nanopolish/2345_reads.sorted.bam -g /home/CAM/lbeka/minION/new_analysis/nanopolish/2345_dec1_new_90x_nanofilt_porechopped.contigs.fasta --min-candidate-frequency 0.1

python /home/CAM/lbeka/minION/new_analysis/nanopolish/nanopolish_makerange.py /home/CAM/lbeka/minION/new_analysis/nanopolish/2347/2347_nov16_dec1_103x_nanofilt_porechopped.contigs.fasta | parallel --results 2347_nanopolish.results -P 24 \ nanopolish variants --consensus polished.{1}.fa -w {1} -r /home/CAM/lbeka/minION/new_analysis/nanopolish/2347/2347_combined_NEW_nanoX_reads.fasta -b /home/CAM/lbeka/minION/new_analysis/nanopolish/2347/2347_reads.sorted.bam -g /home/CAM/lbeka/minION/new_analysis/nanopolish/2347/2347_nov16_dec1_103x_nanofilt_porechopped.contigs.fasta --min-candidate-frequency 0.1

python /home/CAM/lbeka/minION/new_analysis/nanopolish/nanopolish_makerange.py /home/CAM/lbeka/minION/new_analysis/nanopolish/1748/1748_moreX_dec1_46x_nanofilt_porechopped.contigs.fasta | parallel --results 1748_nanopolish.results -P 24 \ nanopolish variants --consensus polished.{1}.fa -w {1} -r /home/CAM/lbeka/minION/new_analysis/nanopolish/1748/1748_nanoX_NEW_dec46Xonly_reads.fasta -b /home/CAM/lbeka/minION/new_analysis/nanopolish/1748/1748_reads.sorted.bam -g /home/CAM/lbeka/minION/new_analysis/nanopolish/1748/1748_moreX_dec1_46x_nanofilt_porechopped.contigs.fasta --min-candidate-frequency 0.1

python /home/CAM/lbeka/minION/new_analysis/nanopolish/nanopolish_makerange.py /home/CAM/lbeka/minION/new_analysis/nanopolish/jg3/jg3_oct_nov_combined_67x_nanofilt_porechopped.contigs.fasta | parallel --results jg3_nanopolish.results -P 24 \ nanopolish variants --consensus polished.{1}.fa -w {1} -r /home/CAM/lbeka/minION/new_analysis/nanopolish/jg3/jg3_combined_nanoX_reads.fasta -b /home/CAM/lbeka/minION/new_analysis/nanopolish/jg3/jg3_reads.sorted.bam -g /home/CAM/lbeka/minION/new_analysis/nanopolish/jg3/jg3_oct_nov_combined_67x_nanofilt_porechopped.contigs.fasta --min-candidate-frequency 0.1

python /home/CAM/lbeka/minION/new_analysis/nanopolish/nanopolish_makerange.py 2348_canu.fasta | parallel --results 2348_nanopolish.results -P 4 \ nanopolish variants --consensus polished.{1}.fa -w {1} -r 2348_THIS_nanoX.fasta -b 2348_reads.sorted.bam -g 2348_canu.fasta --min-candidate-frequency 0.1

python /home/CAM/lbeka/minION/new_analysis/nanopolish/nanopolish_makerange.py 2071_canu.fasta | parallel --results 2071_nanopolish.results -P 10 \ nanopolish variants --consensus polished.{1}.fa -w {1} -r 2071_nanoX_NEW_dec135Xonly_reads.fasta -b 2071_reads.sorted.bam -g 2071_canu.fasta --min-candidate-frequency 0.1

Merging 50kb segments back together to generate polished.fasta assembly

python /home/CAM/sgoldstein/canus/nanopolish_merge.py /home/CAM/sgoldstein/canus/jkh158/polished.*.fa > /home/CAM/sgoldstein/canus/jkh158/jkh158_nanopolished.fa

python /home/CAM/sgoldstein/canus/nanopolish_merge.py /home/CAM/sgoldstein/canus/jkh125/polished.*.fa > /home/CAM/sgoldstein/canus/jkh125/jkh125_nanopolished.fa

python /home/CAM/sgoldstein/canus/nanopolish_merge.py /home/CAM/sgoldstein/canus/jkh144/polished.*.fa > /home/CAM/sgoldstein/canus/jkh144/jkh144_nanopolished.fa

python /home/CAM/lbeka/minION/new_analysis/nanopolish/scripts/nanopolish_merge.py /home/CAM/lbeka/minION/new_analysis/nanopolish/polished.*.fa > /home/CAM/lbeka/minION/new_analysis/nanopolish/2345_new_nanopolished.fa

python /home/CAM/lbeka/minION/new_analysis/nanopolish/scripts/nanopolish_merge.py /home/CAM/lbeka/minION/new_analysis/nanopolish/1748/polished.*.fa > /home/CAM/lbeka/minION/new_analysis/nanopolish/1748/1748_dec46x_nanopolished.fa

python /home/CAM/lbeka/minION/new_analysis/nanopolish/scripts/nanopolish_merge.py /home/CAM/lbeka/minION/new_analysis/nanopolish/2347/polished.*.fa > /home/CAM/lbeka/minION/new_analysis/nanopolish/2347/2347_nanopolished.fa

python /home/CAM/lbeka/minION/new_analysis/nanopolish/scripts/nanopolish_merge.py /home/CAM/lbeka/minION/new_analysis/nanopolish/jg3/polished.*.fa > /home/CAM/lbeka/minION/new_analysis/nanopolish/jg3/jg3_nanopolished.fa

python /home/CAM/lbeka/minION/new_analysis/nanopolish/nanopolish_merge.py polished.*.fa > 2348_nanopolished.fa

python /home/CAM/lbeka/minION/new_analysis/nanopolish/nanopolish_merge.py polished.*.fa > 2071_nanopolished.fa

# Canu + pilon assemblies

Indexing canu assembly

bwa index JKH125_all_minION_reads_nanofilt_porechopped.contigs.fasta

bwa index JKH144_1D_nanofilt_porechopped.contigs.fasta

bwa index JKH158_long_reads_nanofilt_porechopped.contigs.fasta

bwa index 2345_dec1_new_90x_nanofilt_porechopped.contigs.fasta

bwa index 2347_nov16_dec1_103x_nanofilt_porechopped.contigs.fasta

bwa index jg3_oct_nov_combined_67x_nanofilt_porechopped.contigs.fasta

bwa index 1748_moreX_dec1_46x_nanofilt_porechopped.contigs.fasta

bwa index 2071_canu.fasta

bwa index 2348_canu.fasta

Mapping the Illumina reads to the indexed canu assembly

bwa mem -R '@RG\tID:all\tSM:all\tLB:library' /home/CAM/sgoldstein/pilon/jkh158/JKH158_long_reads_nanofilt_porechopped.contigs.fasta /home/CAM/sgoldstein/pilon/jkh158/JKH158_TruSeq_output_R1_paired.fastq /home/CAM/sgoldstein/pilon/jkh158/JKH158_TruSeq_output_R2_paired.fastq > /home/CAM/sgoldstein/pilon/jkh158/jkh158_align.sam

bwa mem -R '@RG\tID:all\tSM:all\tLB:library' /home/CAM/sgoldstein/pilon/jkh144/JKH144_1D_nanofilt_porechopped.contigs.fasta /home/CAM/sgoldstein/pilon/jkh144/JKH144_TruSeq_PCR_Free_R1_concat.fastq /home/CAM/sgoldstein/pilon/jkh144/JKH144_TruSeq_PCR_Free_R2_concat.fastq > /home/CAM/sgoldstein/pilon/jkh144/jkh144_align.sam

bwa mem -R '@RG\tID:all\tSM:all\tLB:library' /home/CAM/sgoldstein/pilon/jkh125/JKH125_all_minION_reads_nanofilt_porechopped.contigs.fasta /home/CAM/sgoldstein/pilon/jkh125/JKH125_TruSeq_output_R1_paired_6_20.fastq /home/CAM/sgoldstein/pilon/jkh125/JKH125_TruSeq_output_R2_paired_6_20.fastq > /home/CAM/sgoldstein/pilon/jkh125/jkh125_align.sam

bwa mem -R '@RG\tID:all\tSM:all\tLB:library' 1748_moreX_dec1_46x_nanofilt_porechopped.contigs.fasta AH1_S1_L001_R1_001_tr.fastq AH1_S1_L001_R2_001_tr.fastq > 1748_align.sam

bwa mem -R '@RG\tID:all\tSM:all\tLB:library' 2071_canu.fasta CIP107763_S35_L001_R2_001_tr.fastq CIP107763_S35_L001_R1_001_tr.fastq > 2071_align.sam

bwa mem -R '@RG\tID:all\tSM:all\tLB:library' 2345_dec1_new_90x_nanofilt_porechopped.contigs.fasta NextGenome-3_S111_L001_R1_001_tr.fastq NextGenome-3_S111_L001_R2_001_tr.fastq > 2345_align.sam

bwa mem -R '@RG\tID:all\tSM:all\tLB:library' 2348_canu.fasta 2348_new_trim_R1_paired.fastq 2348_new_trim_R2_paired.fastq > 2348_align.sam

bwa mem -R '@RG\tID:all\tSM:all\tLB:library' 2347_nov16_dec1_103x_nanofilt_porechopped.contigs.fasta 2347_NextGenome6_R1_tr.fastq 2347_NextGenome6_R2_tr.fastq > 2347_align.sam

bwa mem -R '@RG\tID:all\tSM:all\tLB:library' jg3_oct_nov_combined_67x_nanofilt_porechopped.contigs.fasta jg3_new_trim_R1_paired.fastq jg3_new_trim_R2_paired.fastq > jg3_align.sam

Converting .sam to a .bam file

samtools fixmate -O bam jkh125_align.sam jkh125_align_fixmate.bam

samtools fixmate -O bam jkh144_align.sam jkh144_align_fixmate.bam

samtools fixmate -O bam jkh158_align.sam jkh158_align_fixmate.bam

samtools fixmate -O bam 1748_align.sam 1748_align_fixmate.bam

samtools fixmate -O bam 2071_align.sam 2071_align_fixmate.bam

samtools fixmate -O bam 2345_align.sam 2345_align_fixmate.bam

samtools fixmate -O bam 2348_align.sam 2348_align_fixmate.bam

samtools fixmate -O bam 2347_align.sam 2347_align_fixmate.bam

samtools fixmate -O bam jg3_align.sam jg3_align_fixmate.bam

Sorting the new .bam file

samtools sort -o /home/CAM/sgoldstein/pilon/jkh125/jkh125_sorted.bam -O bam -T temp -@ 10 /home/CAM/sgoldstein/pilon/jkh125/jkh125_align_fixmate.bam

samtools sort -o /home/CAM/sgoldstein/pilon/jkh158/jkh158_sorted.bam -O bam -T temp -@ 10 /home/CAM/sgoldstein/pilon/jkh158/jkh158_align_fixmate.bam

samtools sort -o /home/CAM/sgoldstein/pilon/jkh144/jkh144_sorted.bam -O bam -T temp -@ 10 /home/CAM/sgoldstein/pilon/jkh144/jkh144_align_fixmate.bam

samtools sort -o 1748_sorted.bam -O bam -T temp --threads 20 1748_align_fixmate.bam

samtools sort -o 2071_sorted.bam -O bam -T temp --threads 20 2071_align_fixmate.bam

samtools sort -o 2345_sorted.bam -O bam -T temp 2345_align_fixmate.bam

samtools sort -o 2348_sorted.bam -O bam -T temp 2348_align_fixmate.bam

samtools sort -o 2347_sorted.bam -O bam -T temp 2347_align_fixmate.bam

samtools sort -o jg3_sorted.bam -O bam -T temp jg3_align_fixmate.bam

Indexing the sorted.bam file

samtools index jkh158_sorted.bam

samtools index jkh144_sorted.bam

samtools index jkh125_sorted.bam

samtools index 2345_sorted.bam

samtools index 2347_sorted.bam

samtools index jg3_sorted.bam

samtools index 2348_sorted.bam

samtools index 2071_sorted.bam

samtools index 1748_sorted.bam

Running Pilon

java -Xmx48G -jar $PILON/pilon-1.22.jar --genome /home/CAM/sgoldstein/pilon/jkh144/JKH144_1D_nanofilt_porechopped.contigs.fasta --bam /home/CAM/sgoldstein/pilon/jkh144/jkh144_sorted.bam --output jkh144_pilon_new --outdir jkh144_pilon_new --fix all --changes --verbose --threads 20

java -Xmx48G -jar $PILON/pilon-1.22.jar --genome /home/CAM/sgoldstein/pilon/jkh158/JKH158_long_reads_nanofilt_porechopped.contigs.fasta --bam /home/CAM/sgoldstein/pilon/jkh158/jkh158_sorted.bam --output jkh158_pilon_new --outdir jkh158_pilon_new --fix all --changes --verbose --threads 20

java -Xmx48G -jar $PILON/pilon-1.22.jar --genome /home/CAM/sgoldstein/pilon/jkh125/JKH125_all_minION_reads_nanofilt_porechopped.contigs.fasta --bam /home/CAM/sgoldstein/pilon/jkh125/jkh125_sorted.bam --output jkh125_pilon_new --outdir jkh125_pilon_new --fix all --changes --verbose --threads 20

java -Xmx48G -jar $PILON/pilon-1.22.jar --genome 1748_moreX_dec1_46x_nanofilt_porechopped.contigs.fasta --bam 1748_sorted.bam --output 1748_pilon_new --outdir 1748_pilon_new --fix all --changes --verbose --threads 20

java -Xmx48G -jar $PILON/pilon-1.22.jar --genome 2071_canu.fasta --bam 2071_sorted.bam --output 2071_pilon_new --outdir 2071_pilon_new --fix all --changes --verbose --threads 5

java -Xmx48G -jar $PILON/pilon-1.22.jar --genome 2345_dec1_new_90x_nanofilt_porechopped.contigs.fasta --bam 2345_sorted.bam --output 2345_pilon_new --outdir 2345_pilon_new --fix all --changes --verbose --threads 5

java -Xmx48G -jar $PILON/pilon-1.22.jar --genome 2348_canu.fasta --bam 2348_sorted.bam --output 2348_pilon_new --outdir 2348_pilon_new --fix all --changes --verbose --threads 5

java -Xmx48G -jar $PILON/pilon-1.22.jar --genome 2347_nov16_dec1_103x_nanofilt_porechopped.contigs.fasta --bam 2347_sorted.bam --output 2347_pilon_new --outdir 2347_pilon_new --fix all --changes --verbose --threads 5

java -Xmx48G -jar $PILON/pilon-1.22.jar --genome jg3_oct_nov_combined_67x_nanofilt_porechopped.contigs.fasta --bam jg3_sorted.bam --output jg3_pilon_new --outdir jg3_pilon_new --fix all --changes --verbose --threads 5

# QUAST

~/Tools/quast-4.6.3/quast.py 1748_SPAdes_new.fasta -o 1748_SPAdes_new_QUAST

~/Tools/quast-4.6.3/quast.py 2071_SPAdes.fasta -o 2071_SPAdes_QUAST

~/Tools/quast-4.6.3/quast.py jg3_SPAdes_new.fasta -o jg3_SPAdes_new_QUAST

~/Tools/quast-4.6.3/quast.py 2345_SPAdes.fasta -o 2345_SPAdes_QUAST

~/Tools/quast-4.6.3/quast.py 2347_SPAdes.fasta -o 2347_SPAdes_QUAST

~/Tools/quast-4.6.3/quast.py 2348_SPAdes_new.fasta -o 2348_SPAdes_new_QUAST

~/Tools/quast-4.6.3/quast.py JKH125_SPAdes.fasta -o JKH125_SPAdes_QUAST

~/Tools/quast-4.6.3/quast.py JKH144_SPAdes.fasta -o JKH144_SPAdes_QUAST

~/Tools/quast-4.6.3/quast.py JKH158_SPAdes.fasta -o JKH158_SPAdes_QUAST

~/Tools/quast-4.6.3/quast.py 1748_unicycler_new.fasta -o 1748_unicycler_new_QUAST

~/Tools/quast-4.6.3/quast.py 2071_unicycler.fasta -o 2071_unicycler_QUAST

~/Tools/quast-4.6.3/quast.py jg3_unicycler_new.fasta -o jg3_unicycler_new_QUAST

~/Tools/quast-4.6.3/quast.py 2345_unicycler.fasta -o 2345_unicycler_QUAST

~/Tools/quast-4.6.3/quast.py 2347_unicycler.fasta -o 2347_unicycler_QUAST

~/Tools/quast-4.6.3/quast.py 2348_unicycler_new.fasta -o 2348_unicycler_new_QUAST

~/Tools/quast-4.6.3/quast.py JKH125_unicycler.fasta -o JKH125_unicycler_QUAST

~/Tools/quast-4.6.3/quast.py JKH144_unicycler.fasta -o JKH144_unicycler_QUAST

~/Tools/quast-4.6.3/quast.py JKH158_unicycler.fasta -o JKH158_unicycler_QUAST

~/Tools/quast-4.6.3/quast.py 1748_spades_hybrid_contigs.fasta -o 1748_spades_hybrid_QUAST

~/Tools/quast-4.6.3/quast.py 2071_spades_hybrid_contigs.fasta -o 2071_spades_hybrid_QUAST

~/Tools/quast-4.6.3/quast.py jg3_spades_hybrid_contigs.fasta -o jg3_spades_hybrid_QUAST

~/Tools/quast-4.6.3/quast.py 2345_spades_hybrid_contigs.fasta -o 2345_spades_hybrid_QUAST

~/Tools/quast-4.6.3/quast.py 2347_spades_hybrid_contigs.fasta -o 2347_spades_hybrid_QUAST

~/Tools/quast-4.6.3/quast.py 2348_spades_hybrid_contigs.fasta -o 2348_spades_hybrid_QUAST

~/Tools/quast-4.6.3/quast.py JKH125_spadesHYBRID.fasta -o JKH125_spadesHYBRID_QUAST

~/Tools/quast-4.6.3/quast.py JKH144_new_spadesHYBRID.fasta -o JKH144_new_spadesHYBRID_QUAST

~/Tools/quast-4.6.3/quast.py JKH158_spadesHYBRID.fasta -o JKH158_spadesHYBRID_QUAST

~/Tools/quast-4.6.3/quast.py 1748_unicycler_hybrid.fasta -o 1748_unicycler_hybrid_QUAST

~/Tools/quast-4.6.3/quast.py 2347_unicyclerhybrid.fasta -o 2347_unicyclerhybrid_QUAST

~/Tools/quast-4.6.3/quast.py jg3_unicycler_hybrid.fasta -o jg3_unicycler_hybrid_QUAST

~/Tools/quast-4.6.3/quast.py 2345_unicycler_hybrid.fasta -o 2345_unicycler_hybrid_QUAST

~/Tools/quast-4.6.3/quast.py 2347_unicycler_hybrid.fasta -o 2347_unicycler_hybrid_QUAST

~/Tools/quast-4.6.3/quast.py 2348_unicycler_hybrid.fasta -o 2348_unicycler_hybrid_QUAST

~/Tools/quast-4.6.3/quast.py jkh125_unicyclerHYBRID.fasta -o jkh125_unicyclerHYBRID_QUAST

~/Tools/quast-4.6.3/quast.py jkh125_unicyclerHYBRID.fasta -o jkh125_unicyclerHYBRID_QUAST

~/Tools/quast-4.6.3/quast.py jkh125_unicyclerHYBRID.fasta -o jkh125_unicyclerHYBRID_QUAST

~/Tools/quast-4.6.3/quast.py 1748_canu.fasta -o 1748_canu_QUAST

~/Tools/quast-4.6.3/quast.py 2071_canu.fasta -o 2071_canu_QUAST

~/Tools/quast-4.6.3/quast.py jg3_canu.fasta -o jg3_canu_QUAST

~/Tools/quast-4.6.3/quast.py 2345_canu.fasta -o 2345_canu_QUAST

~/Tools/quast-4.6.3/quast.py 2347_canu.fasta -o 2347_canu_QUAST

~/Tools/quast-4.6.3/quast.py 234_canu.fasta -o 2348_canu_QUAST

~/Tools/quast-4.6.3/quast.py JKH125_canu.fasta -o JKH125_canu_QUAST

~/Tools/quast-4.6.3/quast.py JKH144_canu.fasta -o JKH144_canu_QUAST

~/Tools/quast-4.6.3/quast.py JKH158_canu.fasta -o JKH158_canu_QUAST

~/Tools/quast-4.6.3/quast.py 1748_nanopolished.fasta -o 1748_new_nanopolished_QUAST

~/Tools/quast-4.6.3/quast.py 2345_new_nanopolished.fasta -o 2345_new_nanopolished_QUAST

~/Tools/quast-4.6.3/quast.py 2347_nanopolished.fasta -o 2347_new_nanopolished_QUAST

~/Tools/quast-4.6.3/quast.py 2348_nanopolished.fasta -o 2348_new_nanopolished_QUAST

~/Tools/quast-4.6.3/quast.py jg3_new_nanopolished.fasta -o jg3_new_nanopolished_QUAST

~/Tools/quast-4.6.3/quast.py jkh125_nanopolished.fasta -o jkh125_nanopolished_QUAST

~/Tools/quast-4.6.3/quast.py jkh144_nanopolished.fasta -o jkh125_nanopolished_QUAST

~/Tools/quast-4.6.3/quast.py jkh158_nanopolished.fasta -o jkh125_nanopolished_QUAST

~/Tools/quast-4.6.3/quast.py 1748_pilon.fasta -o 1748_pilon_QUAST

~/Tools/quast-4.6.3/quast.py 2071_pilon.fasta -o 2071_pilon_QUAST

~/Tools/quast-4.6.3/quast.py jg3_pilon_new.fasta -o jg3_pilon_new_QUAST

~/Tools/quast-4.6.3/quast.py 2345_pilon.fasta -o 2345_pilon_QUAST

~/Tools/quast-4.6.3/quast.py 2347_pilon_new.fasta -o 2347_pilon_new_QUAST

~/Tools/quast-4.6.3/quast.py 2348_pilon.fasta -o 2348_pilon_QUAST

~/Tools/quast-4.6.3/quast.py jkh125_pilon.fasta -o jkh125_pilon_QUAST

~/Tools/quast-4.6.3/quast.py jkh144_pilon_new.fasta -o jkh144_pilon_new_QUAST

~/Tools/quast-4.6.3/quast.py jkh158_pilon.fasta -o jkh158_pilon_QUAST

Commands for data in Figure 2

# copy files for each genome into their own directory, run commands in each directory

mash sketch -o JKH125_reference *.fasta

mash info JKH125_reference.msh

mash dist JKH125_reference.msh *.fasta > JKH125_comparison.table

mash sketch -o JKH144_reference *.fasta

mash info JKH144_reference.msh

mash dist JKH144_reference.msh *.fasta > JKH144_comparison.table

mash sketch -o JKH158_reference *.fasta

mash info JKH158_reference.msh

mash dist JKH158_reference.msh *.fasta > JKH158_comparison.table

# make tree

~/Tools/mashtree-master/bin/mashtree *.fasta > JKH_mashtree.out

Commands for data in Figure 3

# SNPS and INDELS

# 1748 Pilon vs spades

echo "nucmer ../completed_assemblies/pilon/1748_pilon.fasta ../completed_assemblies/spades/1748_SPAdes.fasta -p 1748_pilon_vs_SPAdes"

nucmer ../completed_assemblies/pilon/1748_pilon.fasta ../completed_assemblies/spades/1748_SPAdes.fasta -p 1748_pilon_vs_SPAdes

echo "delta-filter -1 1748_pilon_vs_SPAdes.delta > 1748_pilon_vs_SPAdes.delta.filtered.delta"

delta-filter -1 1748_pilon_vs_SPAdes.delta > 1748_pilon_vs_SPAdes.delta.filtered.delta

echo "show-snps 1748_pilon_vs_SPAdes.delta.filtered.delta > 1748_pilon_vs_SPAdes.delta.filtered.snps"

show-snps 1748_pilon_vs_SPAdes.delta.filtered.delta > 1748_pilon_vs_SPAdes.delta.filtered.snps

echo "perl count_SNPS_indels.pl 1748_pilon_vs_SPAdes.delta.filtered.snps 1748_pilon_vs_SPAdes.counts.txt"

perl count_SNPS_indels.pl 1748_pilon_vs_SPAdes.delta.filtered.snps 1748_pilon_vs_SPAdes.counts.txt

# 1748 Pilon vs spades Hybrid

echo "nucmer ../completed_assemblies/pilon/1748_pilon.fasta ../completed_assemblies/spades_hybrid/1748_spadesHYBRID.fasta -p 1748_pilon_vs_S

PAdes-hybrid"

nucmer ../completed_assemblies/pilon/1748_pilon.fasta ../completed_assemblies/spades_hybrid/1748_spades_hybrid.fasta -p 1748_pilon_vs_SPAdes-hybrid

echo "delta-filter -1 1748_pilon_vs_SPAdes-hybrid.delta > 1748_pilon_vs_SPAdes-hybrid.delta.filtered.delta"

delta-filter -1 1748_pilon_vs_SPAdes-hybrid.delta > 1748_pilon_vs_SPAdes-hybrid.delta.filtered.delta

echo "show-snps 1748_pilon_vs_SPAdes-hybrid.delta.filtered.delta > 1748_pilon_vs_SPAdes-hybrid.delta.filtered.snps"

show-snps 1748_pilon_vs_SPAdes-hybrid.delta.filtered.delta > 1748_pilon_vs_SPAdes-hybrid.delta.filtered.snps

echo "perl count_SNPS_indels.pl 1748_pilon_vs_SPAdes-hybrid.delta.filtered.snps 1748_pilon_vs_SPAdes-hybrid.counts.txt"

perl count_SNPS_indels.pl 1748_pilon_vs_SPAdes-hybrid.delta.filtered.snps 1748_pilon_vs_SPAdes-hybrid.counts.txt

# 1748 Pilon vs Unicycler

echo "nucmer ../completed_assemblies/pilon/1748_pilon.fasta ../completed_assemblies/unicycler/1748_unicycler.fasta -p 1748_pilon_vs_unicycler"

nucmer ../completed_assemblies/pilon/1748_pilon.fasta ../completed_assemblies/unicycler/1748_unicycler.fasta -p 1748_pilon_vs_unicycler

echo "delta-filter -1 1748_pilon_vs_unicycler.delta > 1748_pilon_vs_unicycler.delta.filtered.delta"

delta-filter -1 1748_pilon_vs_unicycler.delta > 1748_pilon_vs_unicycler.delta.filtered.delta

echo "show-snps 1748_pilon_vs_unicycler.delta.filtered.delta > 1748_pilon_vs_unicycler.delta.filtered.snps"

show-snps 1748_pilon_vs_unicycler.delta.filtered.delta > 1748_pilon_vs_unicycler.delta.filtered.snps

echo "perl count_SNPS_indels.pl 1748_pilon_vs_unicycler.delta.filtered.snps 1748_pilon_vs_unicycler.counts.txt"

perl count_SNPS_indels.pl 1748_pilon_vs_unicycler.delta.filtered.snps 1748_pilon_vs_unicycler.counts.txt

# 1748 vs unicycler-hybrid

echo "nucmer ../completed_assemblies/pilon/1748_pilon.fasta ../completed_assemblies/unicycler_hybrid/1748_unicyclerHYBRID.fasta -p 1748_pilo

n_vs_unicycler-hybrid"

nucmer ../completed_assemblies/pilon/1748_pilon.fasta ../completed_assemblies/unicycler_hybrid/1748_unicycler_hybrid.fasta -p 1748_pilon_vs_unicycler-hybrid

echo "delta-filter -1 1748_pilon_vs_unicycler-hybrid.delta > 1748_pilon_vs_unicycler-hybrid.delta.filtered.delta"

delta-filter -1 1748_pilon_vs_unicycler-hybrid.delta > 1748_pilon_vs_unicycler-hybrid.delta.filtered.delta

echo "show-snps 1748_pilon_vs_unicycler-hybrid.delta.filtered.delta > 1748_pilon_vs_unicycler-hybrid.delta.filtered.snps"

show-snps 1748_pilon_vs_unicycler-hybrid.delta.filtered.delta > 1748_pilon_vs_unicycler-hybrid.delta.filtered.snps

echo "perl count_SNPS_indels.pl 1748_pilon_vs_unicycler-hybrid.delta.filtered.snps 1748_pilon_vs_unicycler-hybrid.counts.txt"

perl count_SNPS_indels.pl 1748_pilon_vs_unicycler-hybrid.delta.filtered.snps 1748_pilon_vs_unicycler-hybrid.counts.txt

# 1748 vs canu

echo "nucmer ../completed_assemblies/pilon/1748_pilon.fasta ../completed_assemblies/canu/1748_canu.fasta -p 1748_pilon_vs_canu"

nucmer ../completed_assemblies/pilon/1748_pilon.fasta ../completed_assemblies/canu/1748_canu.fasta -p 1748_pilon_vs_canu

echo "delta-filter -1 1748_pilon_vs_canu.delta > 1748_pilon_vs_canu.delta.filtered.delta"

delta-filter -1 1748_pilon_vs_canu.delta > 1748_pilon_vs_canu.delta.filtered.delta

echo "show-snps 1748_pilon_vs_canu.delta.filtered.delta > 1748_pilon_vs_canu.delta.filtered.snps"

show-snps 1748_pilon_vs_canu.delta.filtered.delta > 1748_pilon_vs_canu.delta.filtered.snps

echo "perl count_SNPS_indels.pl 1748_pilon_vs_canu.delta.filtered.snps 1748_pilon_vs_canu.counts.txt"

perl count_SNPS_indels.pl 1748_pilon_vs_canu.delta.filtered.snps 1748_pilon_vs_canu.counts.txt

# 1748 vs nanopolish

echo "nucmer ../completed_assemblies/pilon/1748_pilon.fasta ../completed_assemblies/nanopolish/1748_nanopolished.fasta -p 1748_pilon_vs_nano

polished"

nucmer ../completed_assemblies/pilon/1748_pilon.fasta ../completed_assemblies/nanopolish/1748_nanopolished.fasta -p 1748_pilon_vs_nanopolished

echo "delta-filter -1 1748_pilon_vs_nanopolished.delta > 1748_pilon_vs_nanopolished.delta.filtered.delta"

delta-filter -1 1748_pilon_vs_nanopolished.delta > 1748_pilon_vs_nanopolished.delta.filtered.delta

echo "show-snps 1748_pilon_vs_nanopolished.delta.filtered.delta > 1748_pilon_vs_nanopolished.delta.filtered.snps"

show-snps 1748_pilon_vs_nanopolished.delta.filtered.delta > 1748_pilon_vs_nanopolished.delta.filtered.snps

echo "perl count_SNPS_indels.pl 1748_pilon_vs_nanopolished.delta.filtered.snps 1748_pilon_vs_nanopolished.counts.txt"

perl count_SNPS_indels.pl 1748_pilon_vs_nanopolished.delta.filtered.snps 1748_pilon_vs_nanopolished.counts.txt

# 2071 2071 Pilon vs spades

echo "nucmer ../completed_assemblies/pilon/2071_pilon.fasta ../completed_assemblies/spades/2071_SPAdes.fasta -p 2071_pilon_vs_SPAdes"

nucmer ../completed_assemblies/pilon/2071_pilon.fasta ../completed_assemblies/spades/2071_SPAdes.fasta -p 2071_pilon_vs_SPAdes

echo "delta-filter -1 2071_pilon_vs_SPAdes.delta > 2071_pilon_vs_SPAdes.delta.filtered.delta"

delta-filter -1 2071_pilon_vs_SPAdes.delta > 2071_pilon_vs_SPAdes.delta.filtered.delta

echo "show-snps 2071_pilon_vs_SPAdes.delta.filtered.delta > 2071_pilon_vs_SPAdes.delta.filtered.snps"

show-snps 2071_pilon_vs_SPAdes.delta.filtered.delta > 2071_pilon_vs_SPAdes.delta.filtered.snps

echo "perl count_SNPS_indels.pl 2071_pilon_vs_SPAdes.delta.filtered.snps 2071_pilon_vs_SPAdes.counts.txt"

perl count_SNPS_indels.pl 2071_pilon_vs_SPAdes.delta.filtered.snps 2071_pilon_vs_SPAdes.counts.txt

# 20712071 Pilon vs spades Hybrid

echo "nucmer ../completed_assemblies/pilon/2071_pilon.fasta ../completed_assemblies/spades_hybrid/2071_spadesHYBRID.fasta -p 2071_pilon_vs_S

PAdes-hybrid"

nucmer ../completed_assemblies/pilon/2071_pilon.fasta ../completed_assemblies/spades_hybrid/2071_spades_hybrid.fasta -p 2071_pilon_vs_SPAdes-hybrid

echo "delta-filter -1 2071_pilon_vs_SPAdes-hybrid.delta > 2071_pilon_vs_SPAdes-hybrid.delta.filtered.delta"

delta-filter -1 2071_pilon_vs_SPAdes-hybrid.delta > 2071_pilon_vs_SPAdes-hybrid.delta.filtered.delta

echo "show-snps 2071_pilon_vs_SPAdes-hybrid.delta.filtered.delta > 2071_pilon_vs_SPAdes-hybrid.delta.filtered.snps"

show-snps 2071_pilon_vs_SPAdes-hybrid.delta.filtered.delta > 2071_pilon_vs_SPAdes-hybrid.delta.filtered.snps

echo "perl count_SNPS_indels.pl 2071_pilon_vs_SPAdes-hybrid.delta.filtered.snps 2071_pilon_vs_SPAdes-hybrid.counts.txt"

perl count_SNPS_indels.pl 2071_pilon_vs_SPAdes-hybrid.delta.filtered.snps 2071_pilon_vs_SPAdes-hybrid.counts.txt

# 2071 2071 Pilon vs Unicycler

echo "nucmer ../completed_assemblies/pilon/2071_pilon.fasta ../completed_assemblies/unicycler/2071_unicycler.fasta -p 2071_pilon_vs_unicycler"

nucmer ../completed_assemblies/pilon/2071_pilon.fasta ../completed_assemblies/unicycler/2071_unicycler.fasta -p 2071_pilon_vs_unicycler

echo "delta-filter -1 2071_pilon_vs_unicycler.delta > 2071_pilon_vs_unicycler.delta.filtered.delta"

delta-filter -1 2071_pilon_vs_unicycler.delta > 2071_pilon_vs_unicycler.delta.filtered.delta

echo "show-snps 2071_pilon_vs_unicycler.delta.filtered.delta > 2071_pilon_vs_unicycler.delta.filtered.snps"

show-snps 2071_pilon_vs_unicycler.delta.filtered.delta > 2071_pilon_vs_unicycler.delta.filtered.snps

echo "perl count_SNPS_indels.pl 2071_pilon_vs_unicycler.delta.filtered.snps 2071_pilon_vs_unicycler.counts.txt"

perl count_SNPS_indels.pl 2071_pilon_vs_unicycler.delta.filtered.snps 2071_pilon_vs_unicycler.counts.txt

# 2071 vs unicycler-hybrid

echo "nucmer ../completed_assemblies/pilon/2071_pilon.fasta ../completed_assemblies/unicycler_hybrid/2071_unicyclerHYBRID.fasta -p 2071_pilo

n_vs_unicycler-hybrid"

nucmer ../completed_assemblies/pilon/2071_pilon.fasta ../completed_assemblies/unicycler_hybrid/2071_unicycler_hybrid.fasta -p 2071_pilon_vs_unicycler-hybrid

echo "delta-filter -1 2071_pilon_vs_unicycler-hybrid.delta > 2071_pilon_vs_unicycler-hybrid.delta.filtered.delta"

delta-filter -1 2071_pilon_vs_unicycler-hybrid.delta > 2071_pilon_vs_unicycler-hybrid.delta.filtered.delta

echo "show-snps 2071_pilon_vs_unicycler-hybrid.delta.filtered.delta > 2071_pilon_vs_unicycler-hybrid.delta.filtered.snps"

show-snps 2071_pilon_vs_unicycler-hybrid.delta.filtered.delta > 2071_pilon_vs_unicycler-hybrid.delta.filtered.snps

echo "perl count_SNPS_indels.pl 2071_pilon_vs_unicycler-hybrid.delta.filtered.snps 2071_pilon_vs_unicycler-hybrid.counts.txt"

perl count_SNPS_indels.pl 2071_pilon_vs_unicycler-hybrid.delta.filtered.snps 2071_pilon_vs_unicycler-hybrid.counts.txt

# 2071 vs canu

echo "nucmer ../completed_assemblies/pilon/2071_pilon.fasta ../completed_assemblies/canu/2071_canu.fasta -p 2071_pilon_vs_canu"

nucmer ../completed_assemblies/pilon/2071_pilon.fasta ../completed_assemblies/canu/2071_canu.fasta -p 2071_pilon_vs_canu

echo "delta-filter -1 2071_pilon_vs_canu.delta > 2071_pilon_vs_canu.delta.filtered.delta"

delta-filter -1 2071_pilon_vs_canu.delta > 2071_pilon_vs_canu.delta.filtered.delta

echo "show-snps 2071_pilon_vs_canu.delta.filtered.delta > 2071_pilon_vs_canu.delta.filtered.snps"

show-snps 2071_pilon_vs_canu.delta.filtered.delta > 2071_pilon_vs_canu.delta.filtered.snps

echo "perl count_SNPS_indels.pl 2071_pilon_vs_canu.delta.filtered.snps 2071_pilon_vs_canu.counts.txt"

perl count_SNPS_indels.pl 2071_pilon_vs_canu.delta.filtered.snps 2071_pilon_vs_canu.counts.txt

# 2071 vs nanopolish

echo "nucmer ../completed_assemblies/pilon/2071_pilon.fasta ../completed_assemblies/nanopolish/2071_nanopolished.fasta -p 2071_pilon_vs_nano

polished"

nucmer ../completed_assemblies/pilon/2071_pilon.fasta ../completed_assemblies/nanopolish/2071_nanopolished.fasta -p 2071_pilon_vs_nanopolished

echo "delta-filter -1 2071_pilon_vs_nanopolished.delta > 2071_pilon_vs_nanopolished.delta.filtered.delta"

delta-filter -1 2071_pilon_vs_nanopolished.delta > 2071_pilon_vs_nanopolished.delta.filtered.delta

echo "show-snps 2071_pilon_vs_nanopolished.delta.filtered.delta > 2071_pilon_vs_nanopolished.delta.filtered.snps"

show-snps 2071_pilon_vs_nanopolished.delta.filtered.delta > 2071_pilon_vs_nanopolished.delta.filtered.snps

echo "perl count_SNPS_indels.pl 2071_pilon_vs_nanopolished.delta.filtered.snps 2071_pilon_vs_nanopolished.counts.txt"

perl count_SNPS_indels.pl 2071_pilon_vs_nanopolished.delta.filtered.snps 2071_pilon_vs_nanopolished.counts.txt

# 2345 2345 Pilon vs spades

echo "nucmer ../completed_assemblies/pilon/2345_pilon.fasta ../completed_assemblies/spades/2345_SPAdes.fasta -p 2345_pilon_vs_SPAdes"

nucmer ../completed_assemblies/pilon/2345_pilon.fasta ../completed_assemblies/spades/2345_SPAdes.fasta -p 2345_pilon_vs_SPAdes

echo "delta-filter -1 2345_pilon_vs_SPAdes.delta > 2345_pilon_vs_SPAdes.delta.filtered.delta"

delta-filter -1 2345_pilon_vs_SPAdes.delta > 2345_pilon_vs_SPAdes.delta.filtered.delta

echo "show-snps 2345_pilon_vs_SPAdes.delta.filtered.delta > 2345_pilon_vs_SPAdes.delta.filtered.snps"

show-snps 2345_pilon_vs_SPAdes.delta.filtered.delta > 2345_pilon_vs_SPAdes.delta.filtered.snps

echo "perl count_SNPS_indels.pl 2345_pilon_vs_SPAdes.delta.filtered.snps 2345_pilon_vs_SPAdes.counts.txt"

perl count_SNPS_indels.pl 2345_pilon_vs_SPAdes.delta.filtered.snps 2345_pilon_vs_SPAdes.counts.txt

# 23452345 Pilon vs spades Hybrid

echo "nucmer ../completed_assemblies/pilon/2345_pilon.fasta ../completed_assemblies/spades_hybrid/2345_spadesHYBRID.fasta -p 2345_pilon_vs_S

PAdes-hybrid"

nucmer ../completed_assemblies/pilon/2345_pilon.fasta ../completed_assemblies/spades_hybrid/2345_spades_hybrid.fasta -p 2345_pilon_vs_SPAdes-hybrid

echo "delta-filter -1 2345_pilon_vs_SPAdes-hybrid.delta > 2345_pilon_vs_SPAdes-hybrid.delta.filtered.delta"

delta-filter -1 2345_pilon_vs_SPAdes-hybrid.delta > 2345_pilon_vs_SPAdes-hybrid.delta.filtered.delta

echo "show-snps 2345_pilon_vs_SPAdes-hybrid.delta.filtered.delta > 2345_pilon_vs_SPAdes-hybrid.delta.filtered.snps"

show-snps 2345_pilon_vs_SPAdes-hybrid.delta.filtered.delta > 2345_pilon_vs_SPAdes-hybrid.delta.filtered.snps

echo "perl count_SNPS_indels.pl 2345_pilon_vs_SPAdes-hybrid.delta.filtered.snps 2345_pilon_vs_SPAdes-hybrid.counts.txt"

perl count_SNPS_indels.pl 2345_pilon_vs_SPAdes-hybrid.delta.filtered.snps 2345_pilon_vs_SPAdes-hybrid.counts.txt

# 2345 2345 Pilon vs Unicycler

echo "nucmer ../completed_assemblies/pilon/2345_pilon.fasta ../completed_assemblies/unicycler/2345_unicycler.fasta -p 2345_pilon_vs_unicycler"

nucmer ../completed_assemblies/pilon/2345_pilon.fasta ../completed_assemblies/unicycler/2345_unicycler.fasta -p 2345_pilon_vs_unicycler

echo "delta-filter -1 2345_pilon_vs_unicycler.delta > 2345_pilon_vs_unicycler.delta.filtered.delta"

delta-filter -1 2345_pilon_vs_unicycler.delta > 2345_pilon_vs_unicycler.delta.filtered.delta

echo "show-snps 2345_pilon_vs_unicycler.delta.filtered.delta > 2345_pilon_vs_unicycler.delta.filtered.snps"

show-snps 2345_pilon_vs_unicycler.delta.filtered.delta > 2345_pilon_vs_unicycler.delta.filtered.snps

echo "perl count_SNPS_indels.pl 2345_pilon_vs_unicycler.delta.filtered.snps 2345_pilon_vs_unicycler.counts.txt"

perl count_SNPS_indels.pl 2345_pilon_vs_unicycler.delta.filtered.snps 2345_pilon_vs_unicycler.counts.txt

# 2345 vs unicycler-hybrid

echo "nucmer ../completed_assemblies/pilon/2345_pilon.fasta ../completed_assemblies/unicycler_hybrid/2345_unicyclerHYBRID.fasta -p 2345_pilo

n_vs_unicycler-hybrid"

nucmer ../completed_assemblies/pilon/2345_pilon.fasta ../completed_assemblies/unicycler_hybrid/2345_unicycler_hybrid.fasta -p 2345_pilon_vs_unicycler-hybrid

echo "delta-filter -1 2345_pilon_vs_unicycler-hybrid.delta > 2345_pilon_vs_unicycler-hybrid.delta.filtered.delta"

delta-filter -1 2345_pilon_vs_unicycler-hybrid.delta > 2345_pilon_vs_unicycler-hybrid.delta.filtered.delta

echo "show-snps 2345_pilon_vs_unicycler-hybrid.delta.filtered.delta > 2345_pilon_vs_unicycler-hybrid.delta.filtered.snps"

show-snps 2345_pilon_vs_unicycler-hybrid.delta.filtered.delta > 2345_pilon_vs_unicycler-hybrid.delta.filtered.snps

echo "perl count_SNPS_indels.pl 2345_pilon_vs_unicycler-hybrid.delta.filtered.snps 2345_pilon_vs_unicycler-hybrid.counts.txt"

perl count_SNPS_indels.pl 2345_pilon_vs_unicycler-hybrid.delta.filtered.snps 2345_pilon_vs_unicycler-hybrid.counts.txt

# 2345 vs canu

echo "nucmer ../completed_assemblies/pilon/2345_pilon.fasta ../completed_assemblies/canu/2345_canu.fasta -p 2345_pilon_vs_canu"

nucmer ../completed_assemblies/pilon/2345_pilon.fasta ../completed_assemblies/canu/2345_canu.fasta -p 2345_pilon_vs_canu

echo "delta-filter -1 2345_pilon_vs_canu.delta > 2345_pilon_vs_canu.delta.filtered.delta"

delta-filter -1 2345_pilon_vs_canu.delta > 2345_pilon_vs_canu.delta.filtered.delta

echo "show-snps 2345_pilon_vs_canu.delta.filtered.delta > 2345_pilon_vs_canu.delta.filtered.snps"

show-snps 2345_pilon_vs_canu.delta.filtered.delta > 2345_pilon_vs_canu.delta.filtered.snps

echo "perl count_SNPS_indels.pl 2345_pilon_vs_canu.delta.filtered.snps 2345_pilon_vs_canu.counts.txt"

perl count_SNPS_indels.pl 2345_pilon_vs_canu.delta.filtered.snps 2345_pilon_vs_canu.counts.txt

# 2345 vs nanopolish

echo "nucmer ../completed_assemblies/pilon/2345_pilon.fasta ../completed_assemblies/nanopolish/2345_nanopolished.fasta -p 2345_pilon_vs_nano

polished"

nucmer ../completed_assemblies/pilon/2345_pilon.fasta ../completed_assemblies/nanopolish/2345_new_nanopolished.fasta -p 2345_pilon_vs_nanopolished

echo "delta-filter -1 2345_pilon_vs_nanopolished.delta > 2345_pilon_vs_nanopolished.delta.filtered.delta"

delta-filter -1 2345_pilon_vs_nanopolished.delta > 2345_pilon_vs_nanopolished.delta.filtered.delta

echo "show-snps 2345_pilon_vs_nanopolished.delta.filtered.delta > 2345_pilon_vs_nanopolished.delta.filtered.snps"

show-snps 2345_pilon_vs_nanopolished.delta.filtered.delta > 2345_pilon_vs_nanopolished.delta.filtered.snps

echo "perl count_SNPS_indels.pl 2345_pilon_vs_nanopolished.delta.filtered.snps 2345_pilon_vs_nanopolished.counts.txt"

perl count_SNPS_indels.pl 2345_pilon_vs_nanopolished.delta.filtered.snps 2345_pilon_vs_nanopolished.counts.txt

# 2347 2347 Pilon vs spades

echo "nucmer ../completed_assemblies/pilon/2347_pilon.fasta ../completed_assemblies/spades/2347_SPAdes.fasta -p 2347_pilon_vs_SPAdes"

nucmer ../completed_assemblies/pilon/2347_pilon_new.fasta ../completed_assemblies/spades/2347_SPAdes.fasta -p 2347_pilon_vs_SPAdes

echo "delta-filter -1 2347_pilon_vs_SPAdes.delta > 2347_pilon_vs_SPAdes.delta.filtered.delta"

delta-filter -1 2347_pilon_vs_SPAdes.delta > 2347_pilon_vs_SPAdes.delta.filtered.delta

echo "show-snps 2347_pilon_vs_SPAdes.delta.filtered.delta > 2347_pilon_vs_SPAdes.delta.filtered.snps"

show-snps 2347_pilon_vs_SPAdes.delta.filtered.delta > 2347_pilon_vs_SPAdes.delta.filtered.snps

echo "perl count_SNPS_indels.pl 2347_pilon_vs_SPAdes.delta.filtered.snps 2347_pilon_vs_SPAdes.counts.txt"

perl count_SNPS_indels.pl 2347_pilon_vs_SPAdes.delta.filtered.snps 2347_pilon_vs_SPAdes.counts.txt

# 2347 Pilon vs spades Hybrid

echo "nucmer ../completed_assemblies/pilon/2347_pilon.fasta ../completed_assemblies/spades_hybrid/2347_spadesHYBRID.fasta -p 2347_pilon_vs_S

PAdes-hybrid"

nucmer ../completed_assemblies/pilon/2347_pilon_new.fasta ../completed_assemblies/spades_hybrid/2347_spades_hybrid.fasta -p 2347_pilon_vs_SPAdes-hybrid

echo "delta-filter -1 2347_pilon_vs_SPAdes-hybrid.delta > 2347_pilon_vs_SPAdes-hybrid.delta.filtered.delta"

delta-filter -1 2347_pilon_vs_SPAdes-hybrid.delta > 2347_pilon_vs_SPAdes-hybrid.delta.filtered.delta

echo "show-snps 2347_pilon_vs_SPAdes-hybrid.delta.filtered.delta > 2347_pilon_vs_SPAdes-hybrid.delta.filtered.snps"

show-snps 2347_pilon_vs_SPAdes-hybrid.delta.filtered.delta > 2347_pilon_vs_SPAdes-hybrid.delta.filtered.snps

echo "perl count_SNPS_indels.pl 2347_pilon_vs_SPAdes-hybrid.delta.filtered.snps 2347_pilon_vs_SPAdes-hybrid.counts.txt"

perl count_SNPS_indels.pl 2347_pilon_vs_SPAdes-hybrid.delta.filtered.snps 2347_pilon_vs_SPAdes-hybrid.counts.txt

# 2347 2347 Pilon vs Unicycler

echo "nucmer ../completed_assemblies/pilon/2347_pilon.fasta ../completed_assemblies/unicycler/2347_unicycler.fasta -p 2347_pilon_vs_unicycler"

nucmer ../completed_assemblies/pilon/2347_pilon_new.fasta ../completed_assemblies/unicycler/2347_unicycler.fasta -p 2347_pilon_vs_unicycler

echo "delta-filter -1 2347_pilon_vs_unicycler.delta > 2347_pilon_vs_unicycler.delta.filtered.delta"

delta-filter -1 2347_pilon_vs_unicycler.delta > 2347_pilon_vs_unicycler.delta.filtered.delta

echo "show-snps 2347_pilon_vs_unicycler.delta.filtered.delta > 2347_pilon_vs_unicycler.delta.filtered.snps"

show-snps 2347_pilon_vs_unicycler.delta.filtered.delta > 2347_pilon_vs_unicycler.delta.filtered.snps

echo "perl count_SNPS_indels.pl 2347_pilon_vs_unicycler.delta.filtered.snps 2347_pilon_vs_unicycler.counts.txt"

perl count_SNPS_indels.pl 2347_pilon_vs_unicycler.delta.filtered.snps 2347_pilon_vs_unicycler.counts.txt

# 2347 vs unicycler-hybrid

echo "nucmer ../completed_assemblies/pilon/2347_pilon.fasta ../completed_assemblies/unicycler_hybrid/2347_unicyclerHYBRID.fasta -p 2347_pilo

n_vs_unicycler-hybrid"

nucmer ../completed_assemblies/pilon/2347_pilon_new.fasta ../completed_assemblies/unicycler_hybrid/2347_unicycler_hybrid.fasta -p 2347_pilon_vs_unicycler-hybrid

echo "delta-filter -1 2347_pilon_vs_unicycler-hybrid.delta > 2347_pilon_vs_unicycler-hybrid.delta.filtered.delta"

delta-filter -1 2347_pilon_vs_unicycler-hybrid.delta > 2347_pilon_vs_unicycler-hybrid.delta.filtered.delta

echo "show-snps 2347_pilon_vs_unicycler-hybrid.delta.filtered.delta > 2347_pilon_vs_unicycler-hybrid.delta.filtered.snps"

show-snps 2347_pilon_vs_unicycler-hybrid.delta.filtered.delta > 2347_pilon_vs_unicycler-hybrid.delta.filtered.snps

echo "perl count_SNPS_indels.pl 2347_pilon_vs_unicycler-hybrid.delta.filtered.snps 2347_pilon_vs_unicycler-hybrid.counts.txt"

perl count_SNPS_indels.pl 2347_pilon_vs_unicycler-hybrid.delta.filtered.snps 2347_pilon_vs_unicycler-hybrid.counts.txt

# 2347 vs canu

echo "nucmer ../completed_assemblies/pilon/2347_pilon.fasta ../completed_assemblies/canu/2347_canu.fasta -p 2347_pilon_vs_canu"

nucmer ../completed_assemblies/pilon/2347_pilon_new.fasta ../completed_assemblies/canu/2347_canu.fasta -p 2347_pilon_vs_canu

echo "delta-filter -1 2347_pilon_vs_canu.delta > 2347_pilon_vs_canu.delta.filtered.delta"

delta-filter -1 2347_pilon_vs_canu.delta > 2347_pilon_vs_canu.delta.filtered.delta

echo "show-snps 2347_pilon_vs_canu.delta.filtered.delta > 2347_pilon_vs_canu.delta.filtered.snps"

show-snps 2347_pilon_vs_canu.delta.filtered.delta > 2347_pilon_vs_canu.delta.filtered.snps

echo "perl count_SNPS_indels.pl 2347_pilon_vs_canu.delta.filtered.snps 2347_pilon_vs_canu.counts.txt"

perl count_SNPS_indels.pl 2347_pilon_vs_canu.delta.filtered.snps 2347_pilon_vs_canu.counts.txt

# 2347 vs nanopolish

echo "nucmer ../completed_assemblies/pilon/2347_pilon.fasta ../completed_assemblies/nanopolish/2347_nanopolished.fasta -p 2347_pilon_vs_nano

polished"

nucmer ../completed_assemblies/pilon/2347_pilon_new.fasta ../completed_assemblies/nanopolish/2347_nanopolished.fasta -p 2347_pilon_vs_nanopolished

echo "delta-filter -1 2347_pilon_vs_nanopolished.delta > 2347_pilon_vs_nanopolished.delta.filtered.delta"

delta-filter -1 2347_pilon_vs_nanopolished.delta > 2347_pilon_vs_nanopolished.delta.filtered.delta

echo "show-snps 2347_pilon_vs_nanopolished.delta.filtered.delta > 2347_pilon_vs_nanopolished.delta.filtered.snps"

show-snps 2347_pilon_vs_nanopolished.delta.filtered.delta > 2347_pilon_vs_nanopolished.delta.filtered.snps

echo "perl count_SNPS_indels.pl 2347_pilon_vs_nanopolished.delta.filtered.snps 2347_pilon_vs_nanopolished.counts.txt"

perl count_SNPS_indels.pl 2347_pilon_vs_nanopolished.delta.filtered.snps 2347_pilon_vs_nanopolished.counts.txt

# 2348 Pilon vs spades

echo "nucmer ../completed_assemblies/pilon/2348_pilon.fasta ../completed_assemblies/spades/2348_SPAdes.fasta -p 2348_pilon_vs_SPAdes"

nucmer ../completed_assemblies/pilon/2348_pilon_new.fasta ../completed_assemblies/spades/2348_SPAdes.fasta -p 2348_pilon_vs_SPAdes

echo "delta-filter -1 2348_pilon_vs_SPAdes.delta > 2348_pilon_vs_SPAdes.delta.filtered.delta"

delta-filter -1 2348_pilon_vs_SPAdes.delta > 2348_pilon_vs_SPAdes.delta.filtered.delta

echo "show-snps 2348_pilon_vs_SPAdes.delta.filtered.delta > 2348_pilon_vs_SPAdes.delta.filtered.snps"

show-snps 2348_pilon_vs_SPAdes.delta.filtered.delta > 2348_pilon_vs_SPAdes.delta.filtered.snps

echo "perl count_SNPS_indels.pl 2348_pilon_vs_SPAdes.delta.filtered.snps 2348_pilon_vs_SPAdes.counts.txt"

perl count_SNPS_indels.pl 2348_pilon_vs_SPAdes.delta.filtered.snps 2348_pilon_vs_SPAdes.counts.txt

# 2348 Pilon vs spades Hybrid

echo "nucmer ../completed_assemblies/pilon/2348_pilon.fasta ../completed_assemblies/spades_hybrid/2348_spadesHYBRID.fasta -p 2348_pilon_vs_S

PAdes-hybrid"

nucmer ../completed_assemblies/pilon/2348_pilon_new.fasta ../completed_assemblies/spades_hybrid/2348_spades_hybrid.fasta -p 2348_pilon_vs_SPAdes-hybrid

echo "delta-filter -1 2348_pilon_vs_SPAdes-hybrid.delta > 2348_pilon_vs_SPAdes-hybrid.delta.filtered.delta"

delta-filter -1 2348_pilon_vs_SPAdes-hybrid.delta > 2348_pilon_vs_SPAdes-hybrid.delta.filtered.delta

echo "show-snps 2348_pilon_vs_SPAdes-hybrid.delta.filtered.delta > 2348_pilon_vs_SPAdes-hybrid.delta.filtered.snps"

show-snps 2348_pilon_vs_SPAdes-hybrid.delta.filtered.delta > 2348_pilon_vs_SPAdes-hybrid.delta.filtered.snps

echo "perl count_SNPS_indels.pl 2348_pilon_vs_SPAdes-hybrid.delta.filtered.snps 2348_pilon_vs_SPAdes-hybrid.counts.txt"

perl count_SNPS_indels.pl 2348_pilon_vs_SPAdes-hybrid.delta.filtered.snps 2348_pilon_vs_SPAdes-hybrid.counts.txt

# 2348 2348 Pilon vs Unicycler

echo "nucmer ../completed_assemblies/pilon/2348_pilon.fasta ../completed_assemblies/unicycler/2348_unicycler.fasta -p 2348_pilon_vs_unicycler"

nucmer ../completed_assemblies/pilon/2348_pilon_new.fasta ../completed_assemblies/unicycler/2348_unicycler.fasta -p 2348_pilon_vs_unicycler

echo "delta-filter -1 2348_pilon_vs_unicycler.delta > 2348_pilon_vs_unicycler.delta.filtered.delta"

delta-filter -1 2348_pilon_vs_unicycler.delta > 2348_pilon_vs_unicycler.delta.filtered.delta

echo "show-snps 2348_pilon_vs_unicycler.delta.filtered.delta > 2348_pilon_vs_unicycler.delta.filtered.snps"

show-snps 2348_pilon_vs_unicycler.delta.filtered.delta > 2348_pilon_vs_unicycler.delta.filtered.snps

echo "perl count_SNPS_indels.pl 2348_pilon_vs_unicycler.delta.filtered.snps 2348_pilon_vs_unicycler.counts.txt"

perl count_SNPS_indels.pl 2348_pilon_vs_unicycler.delta.filtered.snps 2348_pilon_vs_unicycler.counts.txt

# 2348 vs unicycler-hybrid

echo "nucmer ../completed_assemblies/pilon/2348_pilon.fasta ../completed_assemblies/unicycler_hybrid/2348_unicyclerHYBRID.fasta -p 2348_pilo

n_vs_unicycler-hybrid"

nucmer ../completed_assemblies/pilon/2348_pilon_new.fasta ../completed_assemblies/unicycler_hybrid/2348_unicycler_hybrid.fasta -p 2348_pilon_vs_unicycler-hybrid

echo "delta-filter -1 2348_pilon_vs_unicycler-hybrid.delta > 2348_pilon_vs_unicycler-hybrid.delta.filtered.delta"

delta-filter -1 2348_pilon_vs_unicycler-hybrid.delta > 2348_pilon_vs_unicycler-hybrid.delta.filtered.delta

echo "show-snps 2348_pilon_vs_unicycler-hybrid.delta.filtered.delta > 2348_pilon_vs_unicycler-hybrid.delta.filtered.snps"

show-snps 2348_pilon_vs_unicycler-hybrid.delta.filtered.delta > 2348_pilon_vs_unicycler-hybrid.delta.filtered.snps

echo "perl count_SNPS_indels.pl 2348_pilon_vs_unicycler-hybrid.delta.filtered.snps 2348_pilon_vs_unicycler-hybrid.counts.txt"

perl count_SNPS_indels.pl 2348_pilon_vs_unicycler-hybrid.delta.filtered.snps 2348_pilon_vs_unicycler-hybrid.counts.txt

# 2348 vs canu

echo "nucmer ../completed_assemblies/pilon/2348_pilon.fasta ../completed_assemblies/canu/2348_canu.fasta -p 2348_pilon_vs_canu"

nucmer ../completed_assemblies/pilon/2348_pilon_new.fasta ../completed_assemblies/canu/2348_canu.fasta -p 2348_pilon_vs_canu

echo "delta-filter -1 2348_pilon_vs_canu.delta > 2348_pilon_vs_canu.delta.filtered.delta"

delta-filter -1 2348_pilon_vs_canu.delta > 2348_pilon_vs_canu.delta.filtered.delta

echo "show-snps 2348_pilon_vs_canu.delta.filtered.delta > 2348_pilon_vs_canu.delta.filtered.snps"

show-snps 2348_pilon_vs_canu.delta.filtered.delta > 2348_pilon_vs_canu.delta.filtered.snps

echo "perl count_SNPS_indels.pl 2348_pilon_vs_canu.delta.filtered.snps 2348_pilon_vs_canu.counts.txt"

perl count_SNPS_indels.pl 2348_pilon_vs_canu.delta.filtered.snps 2348_pilon_vs_canu.counts.txt

# 2348 vs nanopolish

echo "nucmer ../completed_assemblies/pilon/2348_pilon.fasta ../completed_assemblies/nanopolish/2348_nanopolished.fasta -p 2348_pilon_vs_nano

polished"

nucmer ../completed_assemblies/pilon/2348_pilon_new.fasta ../completed_assemblies/nanopolish/2348_nanopolished.fasta -p 2348_pilon_vs_nanopolished

echo "delta-filter -1 2348_pilon_vs_nanopolished.delta > 2348_pilon_vs_nanopolished.delta.filtered.delta"

delta-filter -1 2348_pilon_vs_nanopolished.delta > 2348_pilon_vs_nanopolished.delta.filtered.delta

echo "show-snps 2348_pilon_vs_nanopolished.delta.filtered.delta > 2348_pilon_vs_nanopolished.delta.filtered.snps"

show-snps 2348_pilon_vs_nanopolished.delta.filtered.delta > 2348_pilon_vs_nanopolished.delta.filtered.snps

echo "perl count_SNPS_indels.pl 2348_pilon_vs_nanopolished.delta.filtered.snps 2348_pilon_vs_nanopolished.counts.txt"

perl count_SNPS_indels.pl 2348_pilon_vs_nanopolished.delta.filtered.snps 2348_pilon_vs_nanopolished.counts.txt

# jg3 Pilon vs spades

echo "nucmer ../completed_assemblies/pilon/jg3_pilon.fasta ../completed_assemblies/spades/jg3_SPAdes.fasta -p jg3_pilon_vs_SPAdes"

nucmer ../completed_assemblies/pilon/jg3_pilon_new.fasta ../completed_assemblies/spades/jg3_SPAdes.fasta -p jg3_pilon_vs_SPAdes

echo "delta-filter -1 jg3_pilon_vs_SPAdes.delta > jg3_pilon_vs_SPAdes.delta.filtered.delta"

delta-filter -1 jg3_pilon_vs_SPAdes.delta > jg3_pilon_vs_SPAdes.delta.filtered.delta

echo "show-snps jg3_pilon_vs_SPAdes.delta.filtered.delta > jg3_pilon_vs_SPAdes.delta.filtered.snps"

show-snps jg3_pilon_vs_SPAdes.delta.filtered.delta > jg3_pilon_vs_SPAdes.delta.filtered.snps

echo "perl count_SNPS_indels.pl jg3_pilon_vs_SPAdes.delta.filtered.snps jg3_pilon_vs_SPAdes.counts.txt"

perl count_SNPS_indels.pl jg3_pilon_vs_SPAdes.delta.filtered.snps jg3_pilon_vs_SPAdes.counts.txt

# jg3 Pilon vs spades Hybrid

echo "nucmer ../completed_assemblies/pilon/jg3_pilon.fasta ../completed_assemblies/spades_hybrid/jg3_spadesHYBRID.fasta -p jg3_pilon_vs_S

PAdes-hybrid"

nucmer ../completed_assemblies/pilon/jg3_pilon_new.fasta ../completed_assemblies/spades_hybrid/jg3_spades_hybrid.fasta -p jg3_pilon_vs_SPAdes-hybrid

echo "delta-filter -1 jg3_pilon_vs_SPAdes-hybrid.delta > jg3_pilon_vs_SPAdes-hybrid.delta.filtered.delta"

delta-filter -1 jg3_pilon_vs_SPAdes-hybrid.delta > jg3_pilon_vs_SPAdes-hybrid.delta.filtered.delta

echo "show-snps jg3_pilon_vs_SPAdes-hybrid.delta.filtered.delta > jg3_pilon_vs_SPAdes-hybrid.delta.filtered.snps"

show-snps jg3_pilon_vs_SPAdes-hybrid.delta.filtered.delta > jg3_pilon_vs_SPAdes-hybrid.delta.filtered.snps

echo "perl count_SNPS_indels.pl jg3_pilon_vs_SPAdes-hybrid.delta.filtered.snps jg3_pilon_vs_SPAdes-hybrid.counts.txt"

perl count_SNPS_indels.pl jg3_pilon_vs_SPAdes-hybrid.delta.filtered.snps jg3_pilon_vs_SPAdes-hybrid.counts.txt

# jg3 Pilon vs Unicycler

echo "nucmer ../completed_assemblies/pilon/jg3_pilon.fasta ../completed_assemblies/unicycler/jg3_unicycler.fasta -p jg3_pilon_vs_unicycler"

nucmer ../completed_assemblies/pilon/jg3_pilon_new.fasta ../completed_assemblies/unicycler/jg3_unicycler.fasta -p jg3_pilon_vs_unicycler

echo "delta-filter -1 jg3_pilon_vs_unicycler.delta > jg3_pilon_vs_unicycler.delta.filtered.delta"

delta-filter -1 jg3_pilon_vs_unicycler.delta > jg3_pilon_vs_unicycler.delta.filtered.delta

echo "show-snps jg3_pilon_vs_unicycler.delta.filtered.delta > jg3_pilon_vs_unicycler.delta.filtered.snps"

show-snps jg3_pilon_vs_unicycler.delta.filtered.delta > jg3_pilon_vs_unicycler.delta.filtered.snps

echo "perl count_SNPS_indels.pl jg3_pilon_vs_unicycler.delta.filtered.snps jg3_pilon_vs_unicycler.counts.txt"

perl count_SNPS_indels.pl jg3_pilon_vs_unicycler.delta.filtered.snps jg3_pilon_vs_unicycler.counts.txt

# jg3 vs unicycler-hybrid

echo "nucmer ../completed_assemblies/pilon/jg3_pilon.fasta ../completed_assemblies/unicycler_hybrid/jg3_unicyclerHYBRID.fasta -p jg3_pilo

n_vs_unicycler-hybrid"

nucmer ../completed_assemblies/pilon/jg3_pilon_new.fasta ../completed_assemblies/unicycler_hybrid/jg3_unicycler_hybrid.fasta -p jg3_pilon_vs_unicycler-hybrid

echo "delta-filter -1 jg3_pilon_vs_unicycler-hybrid.delta > jg3_pilon_vs_unicycler-hybrid.delta.filtered.delta"

delta-filter -1 jg3_pilon_vs_unicycler-hybrid.delta > jg3_pilon_vs_unicycler-hybrid.delta.filtered.delta

echo "show-snps jg3_pilon_vs_unicycler-hybrid.delta.filtered.delta > jg3_pilon_vs_unicycler-hybrid.delta.filtered.snps"

show-snps jg3_pilon_vs_unicycler-hybrid.delta.filtered.delta > jg3_pilon_vs_unicycler-hybrid.delta.filtered.snps

echo "perl count_SNPS_indels.pl jg3_pilon_vs_unicycler-hybrid.delta.filtered.snps jg3_pilon_vs_unicycler-hybrid.counts.txt"

perl count_SNPS_indels.pl jg3_pilon_vs_unicycler-hybrid.delta.filtered.snps jg3_pilon_vs_unicycler-hybrid.counts.txt

# jg3 vs canu

echo "nucmer ../completed_assemblies/pilon/jg3_pilon.fasta ../completed_assemblies/canu/jg3_canu.fasta -p jg3_pilon_vs_canu"

nucmer ../completed_assemblies/pilon/jg3_pilon_new.fasta ../completed_assemblies/canu/jg3_canu.fasta -p jg3_pilon_vs_canu

echo "delta-filter -1 jg3_pilon_vs_canu.delta > jg3_pilon_vs_canu.delta.filtered.delta"

delta-filter -1 jg3_pilon_vs_canu.delta > jg3_pilon_vs_canu.delta.filtered.delta

echo "show-snps jg3_pilon_vs_canu.delta.filtered.delta > jg3_pilon_vs_canu.delta.filtered.snps"

show-snps jg3_pilon_vs_canu.delta.filtered.delta > jg3_pilon_vs_canu.delta.filtered.snps

echo "perl count_SNPS_indels.pl jg3_pilon_vs_canu.delta.filtered.snps jg3_pilon_vs_canu.counts.txt"

perl count_SNPS_indels.pl jg3_pilon_vs_canu.delta.filtered.snps jg3_pilon_vs_canu.counts.txt

# jg3 vs nanopolish

echo "nucmer ../completed_assemblies/pilon/jg3_pilon.fasta ../completed_assemblies/nanopolish/jg3_nanopolished.fasta -p jg3_pilon_vs_nano

polished"

nucmer ../completed_assemblies/pilon/jg3_pilon_new.fasta ../completed_assemblies/nanopolish/jg3_new_nanopolished.fasta -p jg3_pilon_vs_nanopolished

echo "delta-filter -1 jg3_pilon_vs_nanopolished.delta > jg3_pilon_vs_nanopolished.delta.filtered.delta"

delta-filter -1 jg3_pilon_vs_nanopolished.delta > jg3_pilon_vs_nanopolished.delta.filtered.delta

echo "show-snps jg3_pilon_vs_nanopolished.delta.filtered.delta > jg3_pilon_vs_nanopolished.delta.filtered.snps"

show-snps jg3_pilon_vs_nanopolished.delta.filtered.delta > jg3_pilon_vs_nanopolished.delta.filtered.snps

echo "perl count_SNPS_indels.pl jg3_pilon_vs_nanopolished.delta.filtered.snps jg3_pilon_vs_nanopolished.counts.txt"

perl count_SNPS_indels.pl jg3_pilon_vs_nanopolished.delta.filtered.snps jg3_pilon_vs_nanopolished.counts.txt

# JKS002056 vs SPAdes

echo "nucmer ../completed_assemblies/pilon/jkh125_pilon.fasta ../completed_assemblies/spades/JKH125_SPAdes.fasta -p jkh125_pilon_vs_SPAdes"

nucmer ../completed_assemblies/pilon/jkh125_pilon.fasta ../completed_assemblies/spades/JKH125_SPAdes.fasta -p jkh125_pilon_vs_SPAdes

echo "delta-filter -1 jkh125_pilon_vs_SPAdes.delta > jkh125_pilon_vs_SPAdes.delta.filtered.delta"

delta-filter -1 jkh125_pilon_vs_SPAdes.delta > jkh125_pilon_vs_SPAdes.delta.filtered.delta

echo "show-snps jkh125_pilon_vs_SPAdes.delta.filtered.delta > jkh125_pilon_vs_SPAdes.delta.filtered.snps"

show-snps jkh125_pilon_vs_SPAdes.delta.filtered.delta > jkh125_pilon_vs_SPAdes.delta.filtered.snps

echo "perl count_SNPS_indels.pl jkh125_pilon_vs_SPAdes.delta.filtered.snps jkh125_pilon_vs_SPAdes.counts.txt"

perl count_SNPS_indels.pl jkh125_pilon_vs_SPAdes.delta.filtered.snps jkh125_pilon_vs_SPAdes.counts.txt

# JKS002056 vs SPAdes-hybrid

echo "nucmer ../completed_assemblies/pilon/jkh125_pilon.fasta ../completed_assemblies/spades_hybrid/jkh125_spadesHYBRID.fasta -p jkh125_pilon_vs_SPAdes-hybrid"

nucmer ../completed_assemblies/pilon/jkh125_pilon.fasta ../completed_assemblies/spades_hybrid/jkh125_spadesHYBRID.fasta -p jkh125_pilon_vs_SPAdes-hybrid

echo "delta-filter -1 jkh125_pilon_vs_SPAdes-hybrid.delta > jkh125_pilon_vs_SPAdes-hybrid.delta.filtered.delta"

delta-filter -1 jkh125_pilon_vs_SPAdes-hybrid.delta > jkh125_pilon_vs_SPAdes-hybrid.delta.filtered.delta

echo "show-snps jkh125_pilon_vs_SPAdes-hybrid.delta.filtered.delta > jkh125_pilon_vs_SPAdes-hybrid.delta.filtered.snps"

show-snps jkh125_pilon_vs_SPAdes-hybrid.delta.filtered.delta > jkh125_pilon_vs_SPAdes-hybrid.delta.filtered.snps

echo "perl count_SNPS_indels.pl jkh125_pilon_vs_SPAdes-hybrid.delta.filtered.snps jkh125_pilon_vs_SPAdes-hybrid.counts.txt"

perl count_SNPS_indels.pl jkh125_pilon_vs_SPAdes-hybrid.delta.filtered.snps jkh125_pilon_vs_SPAdes-hybrid.counts.txt

# JKS002056 vs unicycler

echo "nucmer ../completed_assemblies/pilon/jkh125_pilon.fasta ../completed_assemblies/unicycler/JKH125_unicycler.fasta -p jkh125_pilon_vs_unicycler"

nucmer ../completed_assemblies/pilon/jkh125_pilon.fasta ../completed_assemblies/unicycler/JKH125_unicycler.fasta -p jkh125_pilon_vs_unicycler

echo "delta-filter -1 jkh125_pilon_vs_unicycler.delta > jkh125_pilon_vs_unicycler.delta.filtered.delta"

delta-filter -1 jkh125_pilon_vs_unicycler.delta > jkh125_pilon_vs_unicycler.delta.filtered.delta

echo "show-snps jkh125_pilon_vs_unicycler.delta.filtered.delta > jkh125_pilon_vs_unicycler.delta.filtered.snps"

show-snps jkh125_pilon_vs_unicycler.delta.filtered.delta > jkh125_pilon_vs_unicycler.delta.filtered.snps

echo "perl count_SNPS_indels.pl jkh125_pilon_vs_unicycler.delta.filtered.snps jkh125_pilon_vs_unicycler.counts.txt"

perl count_SNPS_indels.pl jkh125_pilon_vs_unicycler.delta.filtered.snps jkh125_pilon_vs_unicycler.counts.txt

# JKS002056 vs unicycler-hybrid

echo "nucmer ../completed_assemblies/pilon/jkh125_pilon.fasta ../completed_assemblies/unicycler_hybrid/jkh125_unicyclerHYBRID.fasta -p jkh125_pilon_vs_unicycler-hybrid"

nucmer ../completed_assemblies/pilon/jkh125_pilon.fasta ../completed_assemblies/unicycler_hybrid/jkh125_unicyclerHYBRID.fasta -p jkh125_pilon_vs_unicycler-hybrid

echo "delta-filter -1 jkh125_pilon_vs_unicycler-hybrid.delta > jkh125_pilon_vs_unicycler-hybrid.delta.filtered.delta"

delta-filter -1 jkh125_pilon_vs_unicycler-hybrid.delta > jkh125_pilon_vs_unicycler-hybrid.delta.filtered.delta

echo "show-snps jkh125_pilon_vs_unicycler-hybrid.delta.filtered.delta > jkh125_pilon_vs_unicycler-hybrid.delta.filtered.snps"

show-snps jkh125_pilon_vs_unicycler-hybrid.delta.filtered.delta > jkh125_pilon_vs_unicycler-hybrid.delta.filtered.snps

echo "perl count_SNPS_indels.pl jkh125_pilon_vs_unicycler-hybrid.delta.filtered.snps jkh125_pilon_vs_unicycler-hybrid.counts.txt"

perl count_SNPS_indels.pl jkh125_pilon_vs_unicycler-hybrid.delta.filtered.snps jkh125_pilon_vs_unicycler-hybrid.counts.txt

# JKS002056 vs canu

echo "nucmer ../completed_assemblies/pilon/jkh125_pilon.fasta ../completed_assemblies/canu/JKH125_canu.fasta -p jkh125_pilon_vs_canu"

nucmer ../completed_assemblies/pilon/jkh125_pilon.fasta ../completed_assemblies/canu/JKH125_canu.fasta -p jkh125_pilon_vs_canu

echo "delta-filter -1 jkh125_pilon_vs_canu.delta > jkh125_pilon_vs_canu.delta.filtered.delta"

delta-filter -1 jkh125_pilon_vs_canu.delta > jkh125_pilon_vs_canu.delta.filtered.delta

echo "show-snps jkh125_pilon_vs_canu.delta.filtered.delta > jkh125_pilon_vs_canu.delta.filtered.snps"

show-snps jkh125_pilon_vs_canu.delta.filtered.delta > jkh125_pilon_vs_canu.delta.filtered.snps

echo "perl count_SNPS_indels.pl jkh125_pilon_vs_canu.delta.filtered.snps jkh125_pilon_vs_canu.counts.txt"

perl count_SNPS_indels.pl jkh125_pilon_vs_canu.delta.filtered.snps jkh125_pilon_vs_canu.counts.txt

# JKS002056 vs nanopolish

echo "nucmer ../completed_assemblies/pilon/jkh125_pilon.fasta ../completed_assemblies/nanopolish/jkh125_nanopolished.fasta -p jkh125_pilon_vs_nanopolished"

nucmer ../completed_assemblies/pilon/jkh125_pilon.fasta ../completed_assemblies/nanopolish/jkh125_nanopolished.fasta -p jkh125_pilon_vs_nanopolished

echo "delta-filter -1 jkh125_pilon_vs_nanopolished.delta > jkh125_pilon_vs_nanopolished.delta.filtered.delta"

delta-filter -1 jkh125_pilon_vs_nanopolished.delta > jkh125_pilon_vs_nanopolished.delta.filtered.delta

echo "show-snps jkh125_pilon_vs_nanopolished.delta.filtered.delta > jkh125_pilon_vs_nanopolished.delta.filtered.snps"

show-snps jkh125_pilon_vs_nanopolished.delta.filtered.delta > jkh125_pilon_vs_nanopolished.delta.filtered.snps

echo "perl count_SNPS_indels.pl jkh125_pilon_vs_nanopolished.delta.filtered.snps jkh125_pilon_vs_nanopolished.counts.txt"

perl count_SNPS_indels.pl jkh125_pilon_vs_nanopolished.delta.filtered.snps jkh125_pilon_vs_nanopolished.counts.txt

# JKS002072 vs SPAdes

echo "nucmer ../completed_assemblies/pilon/jkh144_pilon_new.fasta ../completed_assemblies/spades/JKH144_SPAdes.fasta -p jkh144_pilon_vs_SPAdes"

nucmer ../completed_assemblies/pilon/jkh144_pilon_new.fasta ../completed_assemblies/spades/JKH144_SPAdes.fasta -p jkh144_pilon_vs_SPAdes

echo "delta-filter -1 jkh144_pilon_vs_SPAdes.delta > jkh144_pilon_vs_SPAdes.delta.filtered.delta"

delta-filter -1 jkh144_pilon_vs_SPAdes.delta > jkh144_pilon_vs_SPAdes.delta.filtered.delta

echo "show-snps jkh144_pilon_vs_SPAdes.delta.filtered.delta > jkh144_pilon_vs_SPAdes.delta.filtered.snps"

show-snps jkh144_pilon_vs_SPAdes.delta.filtered.delta > jkh144_pilon_vs_SPAdes.delta.filtered.snps

echo "perl count_SNPS_indels.pl jkh144_pilon_vs_SPAdes.delta.filtered.snps jkh144_pilon_vs_SPAdes.counts.txt"

perl count_SNPS_indels.pl jkh144_pilon_vs_SPAdes.delta.filtered.snps jkh144_pilon_vs_SPAdes.counts.txt

# JKS002072 vs SPAdes-hybrid

echo "nucmer ../completed_assemblies/pilon/jkh144_pilon_new.fasta ../completed_assemblies/spades_hybrid/jkh144_spadesHYBRID_new.fasta -p jkh144_pilon_vs_SPAdes-hybrid"

nucmer ../completed_assemblies/pilon/jkh144_pilon_new.fasta ../completed_assemblies/spades_hybrid/jkh144_spadesHYBRID_new.fasta -p jkh144_pilon_vs_SPAdes-hybrid

echo "delta-filter -1 jkh144_pilon_vs_SPAdes-hybrid.delta > jkh144_pilon_vs_SPAdes-hybrid.delta.filtered.delta"

delta-filter -1 jkh144_pilon_vs_SPAdes-hybrid.delta > jkh144_pilon_vs_SPAdes-hybrid.delta.filtered.delta

echo "show-snps jkh144_pilon_vs_SPAdes-hybrid.delta.filtered.delta > jkh144_pilon_vs_SPAdes-hybrid.delta.filtered.snps"

show-snps jkh144_pilon_vs_SPAdes-hybrid.delta.filtered.delta > jkh144_pilon_vs_SPAdes-hybrid.delta.filtered.snps

echo "perl count_SNPS_indels.pl jkh144_pilon_vs_SPAdes-hybrid.delta.filtered.snps jkh144_pilon_vs_SPAdes-hybrid.counts.txt"

perl count_SNPS_indels.pl jkh144_pilon_vs_SPAdes-hybrid.delta.filtered.snps jkh144_pilon_vs_SPAdes-hybrid.counts.txt

# JKS002072 vs unicycler

echo "nucmer ../completed_assemblies/pilon/jkh144_pilon_new.fasta ../completed_assemblies/unicycler/JKH144_unicycler.fasta -p jkh144_pilon_vs_unicycler"

nucmer ../completed_assemblies/pilon/jkh144_pilon_new.fasta ../completed_assemblies/unicycler/JKH144_unicycler.fasta -p jkh144_pilon_vs_unicycler

echo "delta-filter -1 jkh144_pilon_vs_unicycler.delta > jkh144_pilon_vs_unicycler.delta.filtered.delta"

delta-filter -1 jkh144_pilon_vs_unicycler.delta > jkh144_pilon_vs_unicycler.delta.filtered.delta

echo "show-snps jkh144_pilon_vs_unicycler.delta.filtered.delta > jkh144_pilon_vs_unicycler.delta.filtered.snps"

show-snps jkh144_pilon_vs_unicycler.delta.filtered.delta > jkh144_pilon_vs_unicycler.delta.filtered.snps

echo "perl count_SNPS_indels.pl jkh144_pilon_vs_unicycler.delta.filtered.snps jkh144_pilon_vs_unicycler.counts.txt"

perl count_SNPS_indels.pl jkh144_pilon_vs_unicycler.delta.filtered.snps jkh144_pilon_vs_unicycler.counts.txt

# JKS002072 vs unicycler-hybrid

echo "nucmer ../completed_assemblies/pilon/jkh144_pilon_new.fasta ../completed_assemblies/unicycler_hybrid/jkh144_unicyclerHYBRID_new.fasta -p jkh144_pilon_vs_unicycler-hybrid"

nucmer ../completed_assemblies/pilon/jkh144_pilon_new.fasta ../completed_assemblies/unicycler_hybrid/jkh144_unicyclerHYBRID_new.fasta -p jkh144_pilon_vs_unicycler-hybrid

echo "delta-filter -1 jkh144_pilon_vs_unicycler-hybrid.delta > jkh144_pilon_vs_unicycler-hybrid.delta.filtered.delta"

delta-filter -1 jkh144_pilon_vs_unicycler-hybrid.delta > jkh144_pilon_vs_unicycler-hybrid.delta.filtered.delta

echo "show-snps jkh144_pilon_vs_unicycler-hybrid.delta.filtered.delta > jkh144_pilon_vs_unicycler-hybrid.delta.filtered.snps"

show-snps jkh144_pilon_vs_unicycler-hybrid.delta.filtered.delta > jkh144_pilon_vs_unicycler-hybrid.delta.filtered.snps

echo "perl count_SNPS_indels.pl jkh144_pilon_vs_unicycler-hybrid.delta.filtered.snps jkh144_pilon_vs_unicycler-hybrid.counts.txt"

perl count_SNPS_indels.pl jkh144_pilon_vs_unicycler-hybrid.delta.filtered.snps jkh144_pilon_vs_unicycler-hybrid.counts.txt

# JKS002072 vs canu

echo "nucmer ../completed_assemblies/pilon/jkh144_pilon_new.fasta ../completed_assemblies/canu/JKH144_canu.fasta -p jkh144_pilon_vs_canu"

nucmer ../completed_assemblies/pilon/jkh144_pilon_new.fasta ../completed_assemblies/canu/JKH144_canu.fasta -p jkh144_pilon_vs_canu

echo "delta-filter -1 jkh144_pilon_vs_canu.delta > jkh144_pilon_vs_canu.delta.filtered.delta"

delta-filter -1 jkh144_pilon_vs_canu.delta > jkh144_pilon_vs_canu.delta.filtered.delta

echo "show-snps jkh144_pilon_vs_canu.delta.filtered.delta > jkh144_pilon_vs_canu.delta.filtered.snps"

show-snps jkh144_pilon_vs_canu.delta.filtered.delta > jkh144_pilon_vs_canu.delta.filtered.snps

echo "perl count_SNPS_indels.pl jkh144_pilon_vs_canu.delta.filtered.snps jkh144_pilon_vs_canu.counts.txt"

perl count_SNPS_indels.pl jkh144_pilon_vs_canu.delta.filtered.snps jkh144_pilon_vs_canu.counts.txt

# JKS002072 vs nanopolish

echo "nucmer ../completed_assemblies/pilon/jkh144_pilon_new.fasta ../completed_assemblies/nanopolish/jkh144_nanopolished.fasta -p jkh144_pilon_vs_nanopolished"

nucmer ../completed_assemblies/pilon/jkh144_pilon_new.fasta ../completed_assemblies/nanopolish/jkh144_nanopolished.fasta -p jkh144_pilon_vs_nanopolished

echo "delta-filter -1 jkh144_pilon_vs_nanopolished.delta > jkh144_pilon_vs_nanopolished.delta.filtered.delta"

delta-filter -1 jkh144_pilon_vs_nanopolished.delta > jkh144_pilon_vs_nanopolished.delta.filtered.delta

echo "show-snps jkh144_pilon_vs_nanopolished.delta.filtered.delta > jkh144_pilon_vs_nanopolished.delta.filtered.snps"

show-snps jkh144_pilon_vs_nanopolished.delta.filtered.delta > jkh144_pilon_vs_nanopolished.delta.filtered.snps

echo "perl count_SNPS_indels.pl jkh144_pilon_vs_nanopolished.delta.filtered.snps jkh144_pilon_vs_nanopolished.counts.txt"

perl count_SNPS_indels.pl jkh144_pilon_vs_nanopolished.delta.filtered.snps jkh144_pilon_vs_nanopolished.counts.txt

# JKS002128 vs SPAdes

echo "nucmer ../completed_assemblies/pilon/jkh158_pilon.fasta ../completed_assemblies/spades/JKH158_SPAdes.fasta -p jkh158_pilon_vs_SPAdes"

nucmer ../completed_assemblies/pilon/jkh158_pilon.fasta ../completed_assemblies/spades/JKH158_SPAdes.fasta -p jkh158_pilon_vs_SPAdes

echo "delta-filter -1 jkh158_pilon_vs_SPAdes.delta > jkh158_pilon_vs_SPAdes.delta.filtered.delta"

delta-filter -1 jkh158_pilon_vs_SPAdes.delta > jkh158_pilon_vs_SPAdes.delta.filtered.delta

echo "show-snps jkh158_pilon_vs_SPAdes.delta.filtered.delta > jkh158_pilon_vs_SPAdes.delta.filtered.snps"

show-snps jkh158_pilon_vs_SPAdes.delta.filtered.delta > jkh158_pilon_vs_SPAdes.delta.filtered.snps

echo "perl count_SNPS_indels.pl jkh158_pilon_vs_SPAdes.delta.filtered.snps jkh158_pilon_vs_SPAdes.counts.txt"

perl count_SNPS_indels.pl jkh158_pilon_vs_SPAdes.delta.filtered.snps jkh158_pilon_vs_SPAdes.counts.txt

# JKS002128 vs SPAdes-hybrid

echo "nucmer ../completed_assemblies/pilon/jkh158_pilon.fasta ../completed_assemblies/spades_hybrid/jkh158_spadesHYBRID.fasta -p jkh158_pilon_vs_SPAdes-hybrid"

nucmer ../completed_assemblies/pilon/jkh158_pilon.fasta ../completed_assemblies/spades_hybrid/jkh158_spadesHYBRID.fasta -p jkh158_pilon_vs_SPAdes-hybrid

echo "delta-filter -1 jkh158_pilon_vs_SPAdes-hybrid.delta > jkh158_pilon_vs_SPAdes-hybrid.delta.filtered.delta"

delta-filter -1 jkh158_pilon_vs_SPAdes-hybrid.delta > jkh158_pilon_vs_SPAdes-hybrid.delta.filtered.delta

echo "show-snps jkh158_pilon_vs_SPAdes-hybrid.delta.filtered.delta > jkh158_pilon_vs_SPAdes-hybrid.delta.filtered.snps"

show-snps jkh158_pilon_vs_SPAdes-hybrid.delta.filtered.delta > jkh158_pilon_vs_SPAdes-hybrid.delta.filtered.snps

echo "perl count_SNPS_indels.pl jkh158_pilon_vs_SPAdes-hybrid.delta.filtered.snps jkh158_pilon_vs_SPAdes-hybrid.counts.txt"

perl count_SNPS_indels.pl jkh158_pilon_vs_SPAdes-hybrid.delta.filtered.snps jkh158_pilon_vs_SPAdes-hybrid.counts.txt

# JKS002128 vs unicycler

echo "nucmer ../completed_assemblies/pilon/jkh158_pilon.fasta ../completed_assemblies/unicycler/JKH158_unicycler.fasta -p jkh158_pilon_vs_unicycler"

nucmer ../completed_assemblies/pilon/jkh158_pilon.fasta ../completed_assemblies/unicycler/JKH158_unicycler.fasta -p jkh158_pilon_vs_unicycler

echo "delta-filter -1 jkh158_pilon_vs_unicycler.delta > jkh158_pilon_vs_unicycler.delta.filtered.delta"

delta-filter -1 jkh158_pilon_vs_unicycler.delta > jkh158_pilon_vs_unicycler.delta.filtered.delta

echo "show-snps jkh158_pilon_vs_unicycler.delta.filtered.delta > jkh158_pilon_vs_unicycler.delta.filtered.snps"

show-snps jkh158_pilon_vs_unicycler.delta.filtered.delta > jkh158_pilon_vs_unicycler.delta.filtered.snps

echo "perl count_SNPS_indels.pl jkh158_pilon_vs_unicycler.delta.filtered.snps jkh158_pilon_vs_unicycler.counts.txt"

perl count_SNPS_indels.pl jkh158_pilon_vs_unicycler.delta.filtered.snps jkh158_pilon_vs_unicycler.counts.txt

# JKS002128 vs unicycler-hybrid

echo "nucmer ../completed_assemblies/pilon/jkh158_pilon.fasta ../completed_assemblies/unicycler_hybrid/jkh158_unicyclerHYBRID.fasta -p jkh158_pilon_vs_unicycler-hybrid"

nucmer ../completed_assemblies/pilon/jkh158_pilon.fasta ../completed_assemblies/unicycler_hybrid/jkh158_unicyclerHYBRID.fasta -p jkh158_pilon_vs_unicycler-hybrid

echo "delta-filter -1 jkh158_pilon_vs_unicycler-hybrid.delta > jkh158_pilon_vs_unicycler-hybrid.delta.filtered.delta"

delta-filter -1 jkh158_pilon_vs_unicycler-hybrid.delta > jkh158_pilon_vs_unicycler-hybrid.delta.filtered.delta

echo "show-snps jkh158_pilon_vs_unicycler-hybrid.delta.filtered.delta > jkh158_pilon_vs_unicycler-hybrid.delta.filtered.snps"

show-snps jkh158_pilon_vs_unicycler-hybrid.delta.filtered.delta > jkh158_pilon_vs_unicycler-hybrid.delta.filtered.snps

echo "perl count_SNPS_indels.pl jkh158_pilon_vs_unicycler-hybrid.delta.filtered.snps jkh158_pilon_vs_unicycler-hybrid.counts.txt"

perl count_SNPS_indels.pl jkh158_pilon_vs_unicycler-hybrid.delta.filtered.snps jkh158_pilon_vs_unicycler-hybrid.counts.txt

# JKS002128 vs canu

echo "nucmer ../completed_assemblies/pilon/jkh158_pilon.fasta ../completed_assemblies/canu/JKH158_canu.fasta -p jkh158_pilon_vs_canu"

nucmer ../completed_assemblies/pilon/jkh158_pilon.fasta ../completed_assemblies/canu/JKH158_canu.fasta -p jkh158_pilon_vs_canu

echo "delta-filter -1 jkh158_pilon_vs_canu.delta > jkh158_pilon_vs_canu.delta.filtered.delta"

delta-filter -1 jkh158_pilon_vs_canu.delta > jkh158_pilon_vs_canu.delta.filtered.delta

echo "show-snps jkh158_pilon_vs_canu.delta.filtered.delta > jkh158_pilon_vs_canu.delta.filtered.snps"

show-snps jkh158_pilon_vs_canu.delta.filtered.delta > jkh158_pilon_vs_canu.delta.filtered.snps

echo "perl count_SNPS_indels.pl jkh158_pilon_vs_canu.delta.filtered.snps jkh158_pilon_vs_canu.counts.txt"

perl count_SNPS_indels.pl jkh158_pilon_vs_canu.delta.filtered.snps jkh158_pilon_vs_canu.counts.txt

# JKS002128 vs nanopolish

echo "nucmer ../completed_assemblies/pilon/jkh158_pilon.fasta ../completed_assemblies/nanopolish/jkh158_nanopolished.fasta -p jkh158_pilon_vs_nanopolished"

nucmer ../completed_assemblies/pilon/jkh158_pilon.fasta ../completed_assemblies/nanopolish/jkh158_nanopolished.fasta -p jkh158_pilon_vs_nanopolished

echo "delta-filter -1 jkh158_pilon_vs_nanopolished.delta > jkh158_pilon_vs_nanopolished.delta.filtered.delta"

delta-filter -1 jkh158_pilon_vs_nanopolished.delta > jkh158_pilon_vs_nanopolished.delta.filtered.delta

echo "show-snps jkh158_pilon_vs_nanopolished.delta.filtered.delta > jkh158_pilon_vs_nanopolished.delta.filtered.snps"

show-snps jkh158_pilon_vs_nanopolished.delta.filtered.delta > jkh158_pilon_vs_nanopolished.delta.filtered.snps

echo "perl count_SNPS_indels.pl jkh158_pilon_vs_nanopolished.delta.filtered.snps jkh158_pilon_vs_nanopolished.counts.txt"

perl count_SNPS_indels.pl jkh158_pilon_vs_nanopolished.delta.filtered.snps jkh158_pilon_vs_nanopolished.counts.txt

#!/usr/bin/perl

#

# count_SNPS_indels.pl

# parses a nucmer SNP table to count the number of SNPs and indels in a genome alignment

use strict;

use warnings;

open (INFILE, $ARGV[0]) or die "Cannot open infile as ARGV[0]";

open (OUTFILE, ">$ARGV[1]") or die "Cannot open outfile as ARGV[1]";

# read through nucmer snps file, count snps and indels

my $indels = 0;

my $snps = 0;

while (my $line = <INFILE>){

my @linearray = split /\s+/, $line;

next unless (scalar @linearray == 16);

if ($linearray[2] eq "." or $linearray[3] eq "."){

$indels++;

}

else {

$snps++;

}

}

close INFILE;

# print to output file

print OUTFILE "Number of SNPS = $snps\n";

print OUTFILE "Number of indels = $indels\n";

close OUTFILE;

Commands for data in Figure 4

### Commands for Supplementary Information

## Prokka Annotations and Anvio Genome Comparisons

# Reformatting each assembly fasta file for Prokka so that output will be compatible with Anvio

Canu assemblies

anvi-script-reformat-fasta 1748_canu.fasta -o 1748_canu_reformat.fasta --min-len 0 --simplify-names

anvi-script-reformat-fasta 2071_canu.fasta -o 2071_canu_reformat.fasta --min-len 0 --simplify-names

anvi-script-reformat-fasta 2345_canu.fasta -o 2345_canu_reformat.fasta --min-len 0 --simplify-names

anvi-script-reformat-fasta 2347_canu.fasta -o 2347_canu_reformat.fasta --min-len 0 --simplify-names

anvi-script-reformat-fasta 2348_canu.fasta -o 2348_canu_reformat.fasta --min-len 0 --simplify-names

anvi-script-reformat-fasta jg3_canu.fasta -o jg3_canu_reformat.fasta --min-len 0 --simplify-names

anvi-script-reformat-fasta JKH125_canu.fasta -o JKH125_canu_reformatted.fasta --min-len 0 --simplify-names

anvi-script-reformat-fasta JKH144_canu.fasta -o JKH144_canu_reformatted.fasta --min-len 0 --simplify-names

anvi-script-reformat-fasta JKH158_canu.fasta -o JKH158_canu_reformatted.fasta --min-len 0 --simplify-names

Nanopolish assemblies

anvi-script-reformat-fasta 1748_nanopolished.fasta -o 1748_nanopolished_reformat.fasta --min-len 0 --simplify-names

anvi-script-reformat-fasta 2071_nanopolished.fasta -o 2071_nanopolished_reformat.fasta --min-len 0 --simplify-names

anvi-script-reformat-fasta 2345_new_nanopolished.fasta -o 2345_new_nanopolished_reformat.fasta --min-len 0 --simplify-names

anvi-script-reformat-fasta 2347_nanopolished.fasta -o 2347_nanopolished_reformat.fasta --min-len 0 --simplify-names

anvi-script-reformat-fasta 2348_nanopolished.fasta -o 2348_nanopolished_reformat.fasta --min-len 0 --simplify-names

anvi-script-reformat-fasta jg3_new_nanopolished.fasta -o jg3_new_nanopolished_reformat.fasta --min-len 0 --simplify-names

anvi-script-reformat-fasta jkh125_nanopolished.fasta -o jkh125_nanopolished_reformatted.fasta --min-len 0 --simplify-names

anvi-script-reformat-fasta jkh144_nanopolished.fasta -o jkh144_nanopolished_reformatted.fasta --min-len 0 --simplify-names

anvi-script-reformat-fasta jkh158_nanopolished.fasta -o jkh158_nanopolished_reformatted.fasta --min-len 0 --simplify-names

Pilon

anvi-script-reformat-fasta 1748_pilon.fasta -o 1748_pilon_reformat.fasta --min-len 0 --simplify-names

anvi-script-reformat-fasta 2071_pilon.fasta -o 2071_pilon_reformat.fasta --min-len 0 --simplify-names

anvi-script-reformat-fasta 2345_pilon.fasta -o 2345_pilon_reformat.fasta --min-len 0 --simplify-names

anvi-script-reformat-fasta 2347_pilon_new.fasta -o 2347_pilon_new_reformat.fasta --min-len 0 --simplify-names

anvi-script-reformat-fasta 2348_pilon_new.fasta -o 2348_pilon_new_reformat.fasta --min-len 0 --simplify-names

anvi-script-reformat-fasta jg3_pilon_new.fasta -o jg3_pilon_new_reformat.fasta --min-len 0 --simplify-names

anvi-script-reformat-fasta jkh144_pilon_new.fasta -o jkh144_pilon_reformatted.fasta --min-len 0 --simplify-names

anvi-script-reformat-fasta jkh125_pilon.fasta -o jkh125_pilon_reformatted.fasta --min-len 0 --simplify-names

anvi-script-reformat-fasta jkh158_pilon.fasta -o jkh158_pilon_reformatted.fasta --min-len 0 --simplify-names

Spades

anvi-script-reformat-fasta 1748_SPAdes_new.fasta -o 1748_SPAdes_new_reformat.fasta --min-len 0 --simplify-names

anvi-script-reformat-fasta 2071_SPAdes.fasta -o 2071_SPAdes_reformat.fasta --min-len 0 --simplify-names

anvi-script-reformat-fasta 2345_SPAdes.fasta -o 2345_SPAdes_reformat.fasta --min-len 0 --simplify-names

anvi-script-reformat-fasta 2347_SPAdes.fasta -o 2347_SPAdes_reformat.fasta --min-len 0 --simplify-names

anvi-script-reformat-fasta 2348_SPAdes_new.fasta -o 2348_SPAdes_new_reformat.fasta --min-len 0 --simplify-names

anvi-script-reformat-fasta jg3_SPAdes_new.fasta -o jg3_SPAdes_new_reformat.fasta --min-len 0 --simplify-names

anvi-script-reformat-fasta JKH125_SPAdes.fasta -o JKH125_SPAdes_reformatted.fasta --min-len 0 --simplify-names

anvi-script-reformat-fasta JKH144_SPAdes.fasta -o JKH144_SPAdes_reformatted.fasta --min-len 0 --simplify-names

anvi-script-reformat-fasta JKH158_SPAdes.fasta -o JKH158_SPAdes_reformatted.fasta --min-len 0 --simplify-names

Spades Hybrid

anvi-script-reformat-fasta 1748_spades_hybrid.fasta -o 1748_spades_hybrid_reformat.fasta --min-len 0 --simplify-names

anvi-script-reformat-fasta 2071_spades_hybrid.fasta -o 2071_spades_hybrid_reformat.fasta --min-len 0 --simplify-names

anvi-script-reformat-fasta 2345_spades_hybrid.fasta -o 2345_spades_hybrid_reformat.fasta --min-len 0 --simplify-names

anvi-script-reformat-fasta 2347_spades_hybrid.fasta -o 2347_spades_hybrid_reformat.fasta --min-len 0 --simplify-names

anvi-script-reformat-fasta 2348_spades_hybrid.fasta -o 2348_spades_hybrid_reformat.fasta --min-len 0 --simplify-names

anvi-script-reformat-fasta jg3_spades_hybrid.fasta -o jg3_spades_hybrid_reformat.fasta --min-len 0 --simplify-names

anvi-script-reformat-fasta jkh144_spadesHYBRID_new.fasta -o jkh144_spadesHYBRID_reformatted.fasta --min-len 0 --simplify-names

anvi-script-reformat-fasta jkh125_spadesHYBRID.fasta -o jkh125_spadesHYBRID_reformatted.fasta --min-len 0 --simplify-names

anvi-script-reformat-fasta jkh158_spadesHYBRID.fasta -o jkh158_spadesHYBRID_reformatted.fasta --min-len 0 --simplify-names

Unicycler

anvi-script-reformat-fasta 1748_unicycler_new.fasta -o 1748_unicycler_new_reformat.fasta --min-len 0 --simplify-names

anvi-script-reformat-fasta 2071_unicycler.fasta -o 2071_unicycler_reformat.fasta --min-len 0 --simplify-names

anvi-script-reformat-fasta 2345_unicycler.fasta -o 2345_unicycler_reformat.fasta --min-len 0 --simplify-names

anvi-script-reformat-fasta 2347_unicycler.fasta -o 2347_unicycler_reformat.fasta --min-len 0 --simplify-names

anvi-script-reformat-fasta 2348_unicycler_new.fasta -o 2348_unicycler_new_reformat.fasta --min-len 0 --simplify-names

anvi-script-reformat-fasta jg3_unicycler_new.fasta -o jg3_unicycler_new_reformat.fasta --min-len 0 --simplify-names

anvi-script-reformat-fasta JKH125_unicycler.fasta -o JKH125_unicycler_reformatted.fasta --min-len 0 --simplify-names

anvi-script-reformat-fasta JKH144_unicycler.fasta -o JKH144_unicycler_reformatted.fasta --min-len 0 --simplify-names

anvi-script-reformat-fasta JKH158_unicycler.fasta -o JKH158_unicycler_reformatted.fasta --min-len 0 --simplify-names

Unicycler Hybrid

anvi-script-reformat-fasta 1748_unicycler_hybrid.fasta -o 1748_unicycler_hybrid_reformat.fasta --min-len 0 --simplify-names

anvi-script-reformat-fasta 2071_unicycler_hybrid.fasta -o 2071_unicycler_hybrid_reformat.fasta --min-len 0 --simplify-names

anvi-script-reformat-fasta 2345_unicycler_hybrid.fasta -o 2345_unicycler_hybrid_reformat.fasta --min-len 0 --simplify-names

anvi-script-reformat-fasta 2347_unicycler_hybrid.fasta -o 2347_unicycler_hybrid_reformat.fasta --min-len 0 --simplify-names

anvi-script-reformat-fasta 2348_unicycler_hybrid.fasta -o 2348_unicycler_hybrid_reformat.fasta --min-len 0 --simplify-names

anvi-script-reformat-fasta jg3_unicycler_hybrid.fasta -o jg3_unicycler_hybrid_reformat.fasta --min-len 0 --simplify-names

anvi-script-reformat-fasta jkh144_unicyclerHYBRID_new.fasta -o jkh144_unicyclerHYBRID_reformatted.fasta --min-len 0 --simplify-names

anvi-script-reformat-fasta jkh125_unicyclerHYBRID.fasta -o jkh125_unicyclerHYBRID_reformatted.fasta --min-len 0 --simplify-names

anvi-script-reformat-fasta jkh158_unicyclerHYBRID.fasta -o jkh158_unicyclerHYBRID_reformatted.fasta --min-len 0 --simplify-names

# Running prokka (Version 1.11)

prokka 1748_canu_reformat.fasta --outdir 1748_canu_prokka_annotation --prefix 1748_canu

prokka 1748_nanopolished_reformat.fasta --outdir 1748_nanopolish_prokka_annotation --prefix 1748_nanopolish

prokka 1748_pilon_reformat.fasta --outdir 1748_pilon_prokka_annotation --prefix 1748_pilon

prokka 1748_spades_hybrid_reformat.fasta --outdir 1748_spades_hybrid_prokka_annotation --prefix 1748_spades_hybrid

prokka 1748_SPAdes_new_reformat.fasta --outdir 1748_SPAdes_prokka_annotation --prefix 1748_SPAdes

prokka 1748_unicycler_hybrid_reformat.fasta --outdir 1748_unicycler_hybrid_prokka_annotation --prefix 1748_unicycler_hybrid

prokka 1748_unicycler_new_reformat.fasta --outdir 1748_unicycler_prokka_annotation --prefix 1748_unicycler

prokka 2347_canu_reformat.fasta --outdir 2347_canu_prokka_annotation --prefix 2347_canu

prokka 2347_nanopolished_reformat.fasta --outdir 2347_nanopolish_prokka_annotation --prefix 2347_nanopolish

prokka 2347_pilon_new_reformat.fasta --outdir 2347_pilon_prokka_annotation --prefix 2347_pilon

prokka 2347_spades_hybrid_reformat.fasta --outdir 2347_spades_hybrid_prokka_annotation --prefix 2347_spades_hybrid

prokka 2347_SPAdes_reformat.fasta --outdir 2347_SPAdes_prokka_annotation --prefix 2347_SPAdes

prokka 2347_unicycler_hybrid_reformat.fasta --outdir 2347_unicycler_hybrid_prokka_annotation --prefix 2347_unicycler_hybrid

prokka 2347_unicycler_reformat.fasta --outdir 2347_unicycler_prokka_annotation --prefix 2347_unicycler

prokka 2345_canu_reformat.fasta --outdir 2345_canu_prokka_annotation --prefix 2345_canu

prokka 2345_new_nanopolished_reformat.fasta --outdir 2345_nanopolish_prokka_annotation --prefix 2345_nanopolish

prokka 2345_pilon_reformat.fasta --outdir 2345_pilon_prokka_annotation --prefix 2345_pilon

prokka 2345_spades_hybrid_reformat.fasta --outdir 2345_spades_hybrid_prokka_annotation --prefix 2345_spades_hybrid

prokka 2345_SPAdes_reformat.fasta --outdir 2345_SPAdes_prokka_annotation --prefix 2345_SPAdes

prokka 2345_unicycler_hybrid_reformat.fasta --outdir 2345_unicycler_hybrid_prokka_annotation --prefix 2345_unicycler_hybrid

prokka 2345_unicycler_reformat.fasta --outdir 2345_unicycler_prokka_annotation --prefix 2345_unicycler

prokka 2071_canu_reformat.fasta --outdir 2071_canu_prokka_annotation --prefix 2071_canu

prokka 2071_nanopolished_reformat.fasta --outdir 2071_nanopolished_prokka_annotation --prefix 2071_nanopolish

prokka 2071_pilon_reformat.fasta --outdir 2071_pilon_prokka_annotation --prefix 2071_pilon

prokka 2071_spades_hybrid_reformat.fasta --outdir 2071_spades_hybrid_prokka_annotation --prefix 2071_spades_hybrid

prokka 2071_SPAdes_reformat.fasta --outdir 2071_SPAdes_prokka_annotation --prefix 2071_SPAdes

prokka 2071_unicycler_hybrid_reformat.fasta --outdir 2071_unicycler_hybrid_prokka_annotation --prefix 2071_unicycler_hybrid

prokka 2071_unicycler_reformat.fasta --outdir 2071_unicycler_prokka_annotation --prefix 2071_unicycler

prokka jg3_canu_reformat.fasta --outdir jg3_canu_prokka_annotation --prefix jg3_canu

prokka jg3_new_nanopolished_reformat.fasta --outdir jg3_new_nanopolished_prokka_annotation --prefix jg3_new_nanopolished

prokka jg3_pilon_new_reformat.fasta --outdir jg3_pilon_prokka_annotation --prefix jg3_pilon

prokka jg3_spades_hybrid_reformat.fasta --outdir jg3_spades_hybrid_prokka_annotation --prefix jg3_spades_hybrid

prokka jg3_SPAdes_new_reformat.fasta --outdir jg3_SPAdes_prokka_annotation --prefix jg3_SPAdes

prokka jg3_unicycler_hybrid_reformat.fasta --outdir jg3_unicycler_hybrid_prokka_annotation --prefix jg3_unicycler_hybrid

prokka jg3_unicycler_new_reformat.fasta --outdir jg3_unicycler_prokka_annotation --prefix jg3_unicycler

prokka 2348_canu_reformat.fasta --outdir 2348_canu_prokka_annotation --prefix 2348_canu

prokka 2348_nanopolished_reformat.fasta --outdir 2348_nanopolished_prokka_annotation --prefix 2348_nanopolished

prokka 2348_pilon_new_reformat.fasta --outdir 2348_pilon_prokka_annotation --prefix 2348_pilon

prokka 2348_spades_hybrid_reformat.fasta --outdir 2348_spades_hybrid_prokka_annotation --prefix 2348_spades_hybrid

prokka 2348_SPAdes_new_reformat.fasta --outdir 2348_SPAdes_prokka_annotation --prefix 2348_SPAdes

prokka 2348_unicycler_hybrid_reformat.fasta --outdir 2348_unicycler_hybrid_prokka_annotation --prefix 2348_unicycler_hybrid

prokka 2348_unicycler_new_reformat.fasta --outdir 2348_unicycler_prokka_annotation --prefix 2348_unicycler

prokka JKH125_canu_reformatted.fasta --outdir JKH125_canu_prokka_annotations --prefix JKH125_canu

prokka jkh125_nanopolished_reformatted.fasta --outdir jkh125_nanopolished_prokka_annotations --prefix jkh125_nanopolished

prokka jkh125_pilon_reformatted.fasta --outdir jkh125_pilon_prokka_annotations --prefix jkh125_pilon

prokka JKH125_SPAdes_reformatted.fasta --outdir JKH125_SPAdes_prokka_annotations --prefix JKH125_SPAdes

prokka jkh125_spadesHYBRID_reformatted.fasta --outdir jkh125_spadesHYBRID_prokka_annotations --prefix jkh125_spadesHYBRID

prokka JKH125_unicycler_reformatted.fasta --outdir JKH125_unicycler_prokka_annotations --prefix JKH125_unicycler

prokka jkh125_unicyclerHYBRID_reformatted.fasta --outdir jkh125_unicyclerHYBRID_prokka_annotations --prefix jkh125_unicyclerHYBRID

prokka JKH144_canu_reformatted.fasta --outdir JKH144_canu_prokka_annotations --prefix JKH144_canu

prokka jkh144_nanopolished_reformatted.fasta --outdir jkh144_nanopolished_prokka_annotations --prefix jkh144_nanopolished

prokka jkh144_pilon_reformatted.fasta --outdir jkh144_pilon_prokka_annotations --prefix jkh144_pilon

prokka JKH144_SPAdes_reformatted.fasta --outdir JKH144_SPAdes_prokka_annotations --prefix JKH144_SPAdes

prokka jkh144_spadesHYBRID_reformatted.fasta --outdir jkh144_spadesHYBRID_prokka_annotations --prefix jkh144_spadesHYBRID

prokka JKH144_unicycler_reformatted.fasta --outdir JKH144_unicycler_prokka_annotations --prefix JKH144_unicycler

prokka jkh144_unicyclerHYBRID_reformatted.fasta --outdir jkh144_unicyclerHYBRID_prokka_annotations --prefix jkh144_unicyclerHYBRID

prokka JKH158_canu_reformatted.fasta --outdir JKH158_canu_prokka_annotations --prefix JKH158_canu

prokka jkh158_nanopolished_reformatted.fasta --outdir jkh158_nanopolished_prokka_annotations --prefix jkh158_nanopolished

prokka jkh158_pilon_reformatted.fasta --outdir jkh158_pilon_prokka_annotations --prefix jkh158_pilon

prokka JKH158_SPAdes_reformatted.fasta --outdir JKH158_SPAdes_prokka_annotations --prefix JKH158_SPAdes

prokka jkh158_spadesHYBRID_reformatted.fasta --outdir jkh158_spadesHYBRID_prokka_annotations --prefix jkh158_spadesHYBRID

prokka JKH158_unicycler_reformatted.fasta --outdir JKH158_unicycler_prokka_annotations --prefix JKH158_unicycler

prokka jkh158_unicyclerHYBRID_reformatted.fasta --outdir jkh158_unicyclerHYBRID_prokka_annotations --prefix jkh158_unicyclerHYBRID

# Using the gff_parser.py script provided by the Meren Lab Github

python gff_parser.py 1748_canu.gff --gene-calls 1748_canu_gene_calls.txt --annotation 1748_canu_annotation.txt

python gff_parser.py 1748_nanopolish.gff --gene-calls 1748_nanopolish_gene_calls.txt --annotation 1748_nanopolish_annotation.txt

python gff_parser.py 1748_pilon.gff --gene-calls 1748_pilon_gene_calls.txt --annotation 1748_pilon_annotation.txt

python gff_parser.py 1748_SPAdes.gff --gene-calls 1748_SPAdes_gene_calls.txt --annotation 1748_SPAdes_annotation.txt

python gff_parser.py 1748_spades_hybrid.gff --gene-calls 1748_spades_hybrid_gene_calls.txt --annotation 1748_spades_hybrid_annotation.txt

python gff_parser.py 1748_unicycler.gff --gene-calls 1748_unicycler_gene_calls.txt --annotation 1748_unicycler_annotation.txt

python gff_parser.py 1748_unicycler_hybrid.gff --gene-calls 1748_unicycler_hybrid_gene_calls.txt --annotation 1748_unicycler_hybrid_annotation.txt

python gff_parser.py 2071_canu.gff --gene-calls 2071_canu_gene_calls.txt --annotation 2071_canu_annotation.txt

python gff_parser.py 2071_nanopolish.gff --gene-calls 2071_nanopolish_gene_calls.txt --annotation 2071_nanopolish_annotation.txt

python gff_parser.py 2071_pilon.gff --gene-calls 2071_pilon_gene_calls.txt --annotation 2071_pilon_annotation.txt

python gff_parser.py 2071_SPAdes.gff --gene-calls 2071_SPAdes_gene_calls.txt --annotation 2071_SPAdes_annotation.txt

python gff_parser.py 2071_spades_hybrid.gff --gene-calls 2071_spades_hybrid_gene_calls.txt --annotation 2071_spades_hybrid_annotation.txt

python gff_parser.py 2071_unicycler.gff --gene-calls 2071_unicycler_gene_calls.txt --annotation 2071_unicycler_annotation.txt

python gff_parser.py 2071_unicycler_hybrid.gff --gene-calls 2071_unicycler_hybrid_gene_calls.txt --annotation 2071_unicycler_hybrid_annotation.txt

python gff_parser.py 2345_canu.gff --gene-calls 2345_canu_gene_calls.txt --annotation 2345_canu_annotation.txt

python gff_parser.py 2345_nanopolish.gff --gene-calls 2345_nanopolish_gene_calls.txt --annotation 2345_nanopolish_annotation.txt

python gff_parser.py 2345_pilon.gff --gene-calls 2345_pilon_gene_calls.txt --annotation 2345_pilon_annotation.txt

python gff_parser.py 2345_SPAdes.gff --gene-calls 2345_SPAdes_gene_calls.txt --annotation 2345_SPAdes_annotation.txt

python gff_parser.py 2345_spades_hybrid.gff --gene-calls 2345_spades_hybrid_gene_calls.txt --annotation 2345_spades_hybrid_annotation.txt

python gff_parser.py 2345_unicycler.gff --gene-calls 2345_unicycler_gene_calls.txt --annotation 2345_unicycler_annotation.txt

python gff_parser.py 2345_unicycler_hybrid.gff --gene-calls 2345_unicycler_hybrid_gene_calls.txt --annotation 2345_unicycler_hybrid_annotation.txt

python gff_parser.py 2347_canu.gff --gene-calls 2347_canu_gene_calls.txt --annotation 2347_canu_annotation.txt

python gff_parser.py 2347_nanopolish.gff --gene-calls 2347_nanopolish_gene_calls.txt --annotation 2347_nanopolish_annotation.txt

python gff_parser.py 2347_pilon.gff --gene-calls 2347_pilon_gene_calls.txt --annotation 2347_pilon_annotation.txt

python gff_parser.py 2347_SPAdes.gff --gene-calls 2347_SPAdes_gene_calls.txt --annotation 2347_SPAdes_annotation.txt

python gff_parser.py 2347_spades_hybrid.gff --gene-calls 2347_spades_hybrid_gene_calls.txt --annotation 2347_spades_hybrid_annotation.txt

python gff_parser.py 2347_unicycler.gff --gene-calls 2347_unicycler_gene_calls.txt --annotation 2347_unicycler_annotation.txt

python gff_parser.py 2347_unicycler_hybrid.gff --gene-calls 2347_unicycler_hybrid_gene_calls.txt --annotation 2347_unicycler_hybrid_annotation.txt

python gff_parser.py 2348_canu.gff --gene-calls 2348_canu_gene_calls.txt --annotation 2348_canu_annotation.txt

python gff_parser.py 2348_nanopolished.gff --gene-calls 2348_nanopolished_gene_calls.txt --annotation 2348_nanopolished_annotation.txt

python gff_parser.py 2348_pilon.gff --gene-calls 2348_pilon_gene_calls.txt --annotation 2348_pilon_annotation.txt

python gff_parser.py 2348_SPAdes.gff --gene-calls 2348_SPAdes_gene_calls.txt --annotation 2348_SPAdes_annotation.txt

python gff_parser.py 2348_spades_hybrid.gff --gene-calls 2348_spades_hybrid_gene_calls.txt --annotation 2348_spades_hybrid_annotation.txt

python gff_parser.py 2348_unicycler.gff --gene-calls 2348_unicycler_gene_calls.txt --annotation 2348_unicycler_annotation.txt

python gff_parser.py 2348_unicycler_hybrid.gff --gene-calls 2348_unicycler_hybrid_gene_calls.txt --annotation 2348_unicycler_hybrid_annotation.txt

python gff_parser.py jg3_canu.gff --gene-calls jg3_canu_gene_calls.txt --annotation jg3_canu_annotation.txt

python gff_parser.py jg3_new_nanopolished.gff --gene-calls jg3_new_nanopolished_gene_calls.txt --annotation jg3_new_nanopolished_annotation.txt

python gff_parser.py jg3_pilon.gff --gene-calls jg3_pilon_gene_calls.txt --annotation jg3_pilon_annotation.txt

python gff_parser.py jg3_SPAdes.gff --gene-calls jg3_SPAdes_gene_calls.txt --annotation jg3_SPAdes_annotation.txt

python gff_parser.py jg3_spades_hybrid.gff --gene-calls jg3_spades_hybrid_gene_calls.txt --annotation jg3_spades_hybrid_annotation.txt

python gff_parser.py jg3_unicycler.gff --gene-calls jg3_unicycler_gene_calls.txt --annotation jg3_unicycler_annotation.txt

python gff_parser.py jg3_unicycler_hybrid.gff --gene-calls jg3_unicycler_hybrid_gene_calls.txt --annotation jg3_unicycler_hybrid_annotation.txt

python gff_parser.py JKH125_SPAdes.gff --gene-calls JKH125_SPAdes_gene_calls.txt --annotation JKH125_SPAdes_annotation.txt

python gff_parser.py JKH125_canu.gff --gene-calls JKH125_canu_gene_calls.txt --annotation JKH125_canu_annotation.txt

python gff_parser.py JKH125_unicycler.gff --gene-calls JKH125_unicycler_gene_calls.txt --annotation JKH125_unicycler_annotation.txt

python gff_parser.py jkh125_nanopolished.gff --gene-calls jkh125_nanopolished_gene_calls.txt --annotation jkh125_nanopolished_annotation.txt

python gff_parser.py jkh125_pilon.gff --gene-calls jkh125_pilon_gene_calls.txt --annotation jkh125_pilon_annotation.txt

python gff_parser.py jkh125_spadesHYBRID.gff --gene-calls jkh125_spadesHYBRID_gene_calls.txt --annotation jkh125_spadesHYBRID_annotation.txt

python gff_parser.py jkh125_unicyclerHYBRID.gff --gene-calls jkh125_unicyclerHYBRID_gene_calls.txt --annotation jkh125_unicyclerHYBRID_annotation.txt

python gff_parser.py JKH144_SPAdes.gff --gene-calls JKH144_SPAdes_gene_calls.txt --annotation JKH144_SPAdes_annotation.txt

python gff_parser.py JKH144_canu.gff --gene-calls JKH144_canu_gene_calls.txt --annotation JKH144_canu_annotation.txt

python gff_parser.py JKH144_unicycler.gff --gene-calls JKH144_unicycler_gene_calls.txt --annotation JKH144_unicycler_annotation.txt

python gff_parser.py jkh144_pilon.gff --gene-calls jkh144_pilon_gene_calls.txt --annotation jkh144_pilon_annotation.txt

python gff_parser.py jkh144_spadesHYBRID.gff --gene-calls jkh144_spadesHYBRID_gene_calls.txt --annotation jkh144_spadesHYBRID_annotation.txt

python gff_parser.py jkh144_unicyclerHYBRID.gff --gene-calls jkh144_unicyclerHYBRID_gene_calls.txt --annotation jkh144_unicyclerHYBRID_annotation.txt

python gff_parser.py jkh144_nanopolished.gff --gene-calls jkh144_nanopolished_gene_calls.txt --annotation jkh144_nanopolished_annotation.txt

python gff_parser.py JKH158_SPAdes.gff --gene-calls JKH158_SPAdes_gene_calls.txt --annotation JKH158_SPAdes_annotation.txt

python gff_parser.py JKH158_canu.gff --gene-calls JKH158_canu_gene_calls.txt --annotation JKH158_canu_annotation.txt

python gff_parser.py JKH158_unicycler.gff --gene-calls JKH158_unicycler_gene_calls.txt --annotation JKH158_unicycler_annotation.txt

python gff_parser.py jkh158_nanopolished.gff --gene-calls jkh158_nanopolished_gene_calls.txt --annotation jkh158_nanopolished_annotation.txt

python gff_parser.py jkh158_pilon.gff --gene-calls jkh158_pilon_gene_calls.txt --annotation jkh158_pilon_annotation.txt

python gff_parser.py jkh158_spadesHYBRID.gff --gene-calls jkh158_spadesHYBRID_gene_calls.txt --annotation jkh158_spadesHYBRID_annotation.txt

python gff_parser.py jkh158_unicyclerHYBRID.gff --gene-calls jkh158_unicyclerHYBRID_gene_calls.txt --annotation jkh158_unicyclerHYBRID_annotation.txt

### Anvio

Anvi'o version ...............................: margaret (v5.2)

Profile DB version ...........................: 30

Contigs DB version ...........................: 12

Pan DB version ...............................: 12

Genome data storage version ..................: 6

Auxiliary data storage version ...............: 2

Structure DB version .........................: 1

# Create new database with the Prokka annotations and import functions

anvi-gen-contigs-database -f 1748_canu_reformat.fasta -o 1748_canu_prokka.db --external-gene-calls 1748_canu_gene_calls.txt -n 1748_canu_prokka_database

anvi-import-functions -c 1748_canu_prokka.db -i 1748_canu_annotation.txt

anvi-gen-contigs-database -f 1748_nanopolished_reformat.fasta -o 1748_nanopolish_prokka.db --external-gene-calls 1748_nanopolish_gene_calls.txt -n 1748_nanopolish_prokka_database

anvi-import-functions -c 1748_nanopolish_prokka.db -i 1748_nanopolish_annotation.txt

anvi-gen-contigs-database -f 1748_pilon_reformat.fasta -o 1748_pilon_prokka.db --external-gene-calls 1748_pilon_gene_calls.txt -n 1748_pilon_prokka_database

anvi-import-functions -c 1748_pilon_prokka.db -i 1748_pilon_annotation.txt

anvi-gen-contigs-database -f 1748_SPAdes_new_reformat.fasta -o 1748_SPAdes_prokka.db --external-gene-calls 1748_SPAdes_gene_calls.txt -n 1748_SPAdes_prokka_database

anvi-import-functions -c 1748_SPAdes_prokka.db -i 1748_SPAdes_annotation.txt

anvi-gen-contigs-database -f 1748_spades_hybrid_reformat.fasta -o 1748_spades_hybrid_prokka.db --external-gene-calls 1748_spades_hybrid_gene_calls.txt -n 1748_spades_hybrid_prokka_database

anvi-import-functions -c 1748_spades_hybrid_prokka.db -i 1748_spades_hybrid_annotation.txt

anvi-gen-contigs-database -f 1748_unicycler_new_reformat.fasta -o 1748_unicycler_prokka.db --external-gene-calls 1748_unicycler_gene_calls.txt -n 1748_unicycler_prokka_database

anvi-import-functions -c 1748_unicycler_prokka.db -i 1748_unicycler_annotation.txt

anvi-gen-contigs-database -f 1748_unicycler_hybrid_reformat.fasta -o 1748_unicycler_hybrid_prokka.db --external-gene-calls 1748_unicycler_hybrid_gene_calls.txt -n 1748_unicycler_hybrid_prokka_database

anvi-import-functions -c 1748_unicycler_hybrid_prokka.db -i 1748_unicycler_hybrid_annotation.txt

anvi-gen-contigs-database -f 2071_canu_reformat.fasta -o 2071_canu_prokka.db --external-gene-calls 2071_canu_gene_calls.txt -n 2071_canu_prokka_database

anvi-import-functions -c 2071_canu_prokka.db -i 2071_canu_annotation.txt

anvi-gen-contigs-database -f 2071_nanopolished_reformat.fasta -o 2071_nanopolish_prokka.db --external-gene-calls 2071_nanopolish_gene_calls.txt -n 2071_nanopolish_prokka_database

anvi-import-functions -c 2071_nanopolish_prokka.db -i 2071_nanopolish_annotation.txt

anvi-gen-contigs-database -f 2071_pilon_reformat.fasta -o 2071_pilon_prokka.db --external-gene-calls 2071_pilon_gene_calls.txt -n 2071_pilon_prokka_database

anvi-import-functions -c 2071_pilon_prokka.db -i 2071_pilon_annotation.txt

anvi-gen-contigs-database -f 2071_SPAdes_reformat.fasta -o 2071_SPAdes_prokka.db --external-gene-calls 2071_SPAdes_gene_calls.txt -n 2071_SPAdes_prokka_database

anvi-import-functions -c 2071_SPAdes_prokka.db -i 2071_SPAdes_annotation.txt

anvi-gen-contigs-database -f 2071_spades_hybrid_reformat.fasta -o 2071_spades_hybrid_prokka.db --external-gene-calls 2071_spades_hybrid_gene_calls.txt -n 2071_spades_hybrid_prokka_database

anvi-import-functions -c 2071_spades_hybrid_prokka.db -i 2071_spades_hybrid_annotation.txt

anvi-gen-contigs-database -f 2071_unicycler_reformat.fasta -o 2071_unicycler_prokka.db --external-gene-calls 2071_unicycler_gene_calls.txt -n 2071_unicycler_prokka_database

anvi-import-functions -c 2071_unicycler_prokka.db -i 2071_unicycler_annotation.txt

anvi-gen-contigs-database -f 2071_unicycler_hybrid_reformat.fasta -o 2071_unicycler_hybrid_prokka.db --external-gene-calls 2071_unicycler_hybrid_gene_calls.txt -n 2071_unicycler_hybrid_prokka_database

anvi-import-functions -c 2071_unicycler_hybrid_prokka.db -i 2071_unicycler_hybrid_annotation.txt

anvi-gen-contigs-database -f 2345_canu_reformat.fasta -o 2345_canu_prokka.db --external-gene-calls 2345_canu_gene_calls.txt -n 2345_canu_prokka_database

anvi-import-functions -c 2345_canu_prokka.db -i 2345_canu_annotation.txt

anvi-gen-contigs-database -f 2345_new_nanopolished_reformat.fasta -o 2345_nanopolish_prokka.db --external-gene-calls 2345_nanopolish_gene_calls.txt -n 2345_nanopolish_prokka_database

anvi-import-functions -c 2345_nanopolish_prokka.db -i 2345_nanopolish_annotation.txt

anvi-gen-contigs-database -f 2345_pilon_reformat.fasta -o 2345_pilon_prokka.db --external-gene-calls 2345_pilon_gene_calls.txt -n 2345_pilon_prokka_database

anvi-import-functions -c 2345_pilon_prokka.db -i 2345_pilon_annotation.txt

anvi-gen-contigs-database -f 2345_SPAdes_reformat.fasta -o 2345_SPAdes_prokka.db --external-gene-calls 2345_SPAdes_gene_calls.txt -n 2345_SPAdes_prokka_database

anvi-import-functions -c 2345_SPAdes_prokka.db -i 2345_SPAdes_annotation.txt

anvi-gen-contigs-database -f 2345_spades_hybrid_reformat.fasta -o 2345_spades_hybrid_prokka.db --external-gene-calls 2345_spades_hybrid_gene_calls.txt -n 2345_spades_hybrid_prokka_database

anvi-import-functions -c 2345_spades_hybrid_prokka.db -i 2345_spades_hybrid_annotation.txt

anvi-gen-contigs-database -f 2345_unicycler_reformat.fasta -o 2345_unicycler_prokka.db --external-gene-calls 2345_unicycler_gene_calls.txt -n 2345_unicycler_prokka_database

anvi-import-functions -c 2345_unicycler_prokka.db -i 2345_unicycler_annotation.txt

anvi-gen-contigs-database -f 2345_unicycler_hybrid_reformat.fasta -o 2345_unicycler_hybrid_prokka.db --external-gene-calls 2345_unicycler_hybrid_gene_calls.txt -n 2345_unicycler_hybrid_prokka_database

anvi-import-functions -c 2345_unicycler_hybrid_prokka.db -i 2345_unicycler_hybrid_annotation.txt

anvi-gen-contigs-database -f 2347_canu_reformat.fasta -o 2347_canu_prokka.db --external-gene-calls 2347_canu_gene_calls.txt -n 2347_canu_prokka_database

anvi-import-functions -c 2347_canu_prokka.db -i 2347_canu_annotation.txt

anvi-gen-contigs-database -f 2347_nanopolished_reformat.fasta -o 2347_nanopolish_prokka.db --external-gene-calls 2347_nanopolish_gene_calls.txt -n 2347_nanopolish_prokka_database

anvi-import-functions -c 2347_nanopolish_prokka.db -i 2347_nanopolish_annotation.txt

anvi-gen-contigs-database -f 2347_pilon_new_reformat.fasta -o 2347_pilon_prokka.db --external-gene-calls 2347_pilon_gene_calls.txt -n 2347_pilon_prokka_database

anvi-import-functions -c 2347_pilon_prokka.db -i 2347_pilon_annotation.txt

anvi-gen-contigs-database -f 2347_SPAdes_reformat.fasta -o 2347_SPAdes_prokka.db --external-gene-calls 2347_SPAdes_gene_calls.txt -n 2347_SPAdes_prokka_database

anvi-import-functions -c 2347_SPAdes_prokka.db -i 2347_SPAdes_annotation.txt

anvi-gen-contigs-database -f 2347_spades_hybrid_reformat.fasta -o 2347_spades_hybrid_prokka.db --external-gene-calls 2347_spades_hybrid_gene_calls.txt -n 2347_spades_hybrid_prokka_database

anvi-import-functions -c 2347_spades_hybrid_prokka.db -i 2347_spades_hybrid_annotation.txt

anvi-gen-contigs-database -f 2347_unicycler_reformat.fasta -o 2347_unicycler_prokka.db --external-gene-calls 2347_unicycler_gene_calls.txt -n 2347_unicycler_prokka_database

anvi-import-functions -c 2347_unicycler_prokka.db -i 2347_unicycler_annotation.txt

anvi-gen-contigs-database -f 2347_unicycler_hybrid_reformat.fasta -o 2347_unicycler_hybrid_prokka.db --external-gene-calls 2347_unicycler_hybrid_gene_calls.txt -n 2347_unicycler_hybrid_prokka_database

anvi-import-functions -c 2347_unicycler_hybrid_prokka.db -i 2347_unicycler_hybrid_annotation.txt

anvi-gen-contigs-database -f 2348_canu_reformat.fasta -o 2348_canu_prokka.db --external-gene-calls 2348_canu_gene_calls.txt -n 2348_canu_prokka_database

anvi-import-functions -c 2348_canu_prokka.db -i 2348_canu_annotation.txt

anvi-gen-contigs-database -f 2348_nanopolished_reformat.fasta -o 2348_nanopolished_prokka.db --external-gene-calls 2348_nanopolished_gene_calls.txt -n 2348_nanopolished_prokka_database

anvi-import-functions -c 2348_nanopolished_prokka.db -i 2348_nanopolished_annotation.txt

anvi-gen-contigs-database -f 2348_pilon_new_reformat.fasta -o 2348_pilon_prokka.db --external-gene-calls 2348_pilon_gene_calls.txt -n 2348_pilon_prokka_database

anvi-import-functions -c 2348_pilon_prokka.db -i 2348_pilon_annotation.txt

anvi-gen-contigs-database -f 2348_SPAdes_new_reformat.fasta -o 2348_SPAdes_prokka.db --external-gene-calls 2348_SPAdes_gene_calls.txt -n 2348_SPAdes_prokka_database

anvi-import-functions -c 2348_SPAdes_prokka.db -i 2348_SPAdes_annotation.txt

anvi-gen-contigs-database -f 2348_spades_hybrid_reformat.fasta -o 2348_spades_hybrid_prokka.db --external-gene-calls 2348_spades_hybrid_gene_calls.txt -n 2348_spades_hybrid_prokka_database

anvi-import-functions -c 2348_spades_hybrid_prokka.db -i 2348_spades_hybrid_annotation.txt

anvi-gen-contigs-database -f 2348_unicycler_new_reformat.fasta -o 2348_unicycler_prokka.db --external-gene-calls 2348_unicycler_gene_calls.txt -n 2348_unicycler_prokka_database

anvi-import-functions -c 2348_unicycler_prokka.db -i 2348_unicycler_annotation.txt

anvi-gen-contigs-database -f 2348_unicycler_hybrid_reformat.fasta -o 2348_unicycler_hybrid_prokka.db --external-gene-calls 2348_unicycler_hybrid_gene_calls.txt -n 2348_unicycler_hybrid_prokka_database

anvi-import-functions -c 2348_unicycler_hybrid_prokka.db -i 2348_unicycler_hybrid_annotation.txt

anvi-gen-contigs-database -f jg3_canu_reformat.fasta -o jg3_canu_prokka.db --external-gene-calls jg3_canu_gene_calls.txt -n jg3_canu_prokka_database

anvi-import-functions -c jg3_canu_prokka.db -i jg3_canu_annotation.txt

anvi-gen-contigs-database -f jg3_new_nanopolished_reformat.fasta -o jg3_new_nanopolished_prokka.db --external-gene-calls jg3_new_nanopolished_gene_calls.txt -n jg3_new_nanopolished_prokka_database

anvi-import-functions -c jg3_new_nanopolished_prokka.db -i jg3_new_nanopolished_annotation.txt

anvi-gen-contigs-database -f jg3_pilon_new_reformat.fasta -o jg3_pilon_prokka.db --external-gene-calls jg3_pilon_gene_calls.txt -n jg3_pilon_prokka_database

anvi-import-functions -c jg3_pilon_prokka.db -i jg3_pilon_annotation.txt

anvi-gen-contigs-database -f jg3_SPAdes_new_reformat.fasta -o jg3_SPAdes_prokka.db --external-gene-calls jg3_SPAdes_gene_calls.txt -n jg3_SPAdes_prokka_database

anvi-import-functions -c jg3_SPAdes_prokka.db -i jg3_SPAdes_annotation.txt

anvi-gen-contigs-database -f jg3_spades_hybrid_reformat.fasta -o jg3_spades_hybrid_prokka.db --external-gene-calls jg3_spades_hybrid_gene_calls.txt -n jg3_spades_hybrid_prokka_database

anvi-import-functions -c jg3_spades_hybrid_prokka.db -i jg3_spades_hybrid_annotation.txt

anvi-gen-contigs-database -f jg3_unicycler_new_reformat.fasta -o jg3_unicycler_prokka.db --external-gene-calls jg3_unicycler_gene_calls.txt -n jg3_unicycler_prokka_database

anvi-import-functions -c jg3_unicycler_prokka.db -i jg3_unicycler_annotation.txt

anvi-gen-contigs-database -f jg3_unicycler_hybrid_reformat.fasta -o jg3_unicycler_hybrid_prokka.db --external-gene-calls jg3_unicycler_hybrid_gene_calls.txt -n jg3_unicycler_hybrid_prokka_database

anvi-import-functions -c jg3_unicycler_hybrid_prokka.db -i jg3_unicycler_hybrid_annotation.txt

anvi-gen-contigs-database -f JKH125_SPAdes_reformatted.fasta -o JKH125_SPAdes_prokka.db --external-gene-calls JKH125_SPAdes_gene_calls.txt -n JKH125_SPAdes_prokka_database

anvi-import-functions -c JKH125_SPAdes_prokka.db -i JKH125_SPAdes_annotation.txt

anvi-gen-contigs-database -f JKH125_canu_reformatted.fasta -o JKH125_canu_prokka.db --external-gene-calls JKH125_canu_gene_calls.txt -n JKH125_canu_prokka_database

anvi-import-functions -c JKH125_canu_prokka.db -i JKH125_canu_annotation.txt

anvi-gen-contigs-database -f JKH125_unicycler_reformatted.fasta -o JKH125_unicycler_prokka.db --external-gene-calls JKH125_unicycler_gene_calls.txt -n JKH125_unicycler_prokka_database

anvi-import-functions -c JKH125_unicycler_prokka.db -i JKH125_unicycler_annotation.txt

anvi-gen-contigs-database -f jkh125_nanopolished_reformatted.fasta -o jkh125_nanopolished_prokka.db --external-gene-calls jkh125_nanopolished_gene_calls.txt -n jkh125_nanopolished_prokka_database

anvi-import-functions -c jkh125_nanopolished_prokka.db -i jkh125_nanopolished_annotation.txt

anvi-gen-contigs-database -f jkh125_pilon_reformatted.fasta -o jkh125_pilon_prokka.db --external-gene-calls jkh125_pilon_gene_calls.txt -n jkh125_pilon_prokka_database

anvi-import-functions -c jkh125_pilon_prokka.db -i jkh125_pilon_annotation.txt

anvi-gen-contigs-database -f jkh125_spadesHYBRID_reformatted.fasta -o jkh125_spadesHYBRID_prokka.db --external-gene-calls jkh125_spadesHYBRID_gene_calls.txt -n jkh125_spadesHYBRID_prokka_database

anvi-import-functions -c jkh125_spadesHYBRID_prokka.db -i jkh125_spadesHYBRID_annotation.txt

anvi-gen-contigs-database -f jkh125_unicyclerHYBRID_reformatted.fasta -o jkh125_unicyclerHYBRID_prokka.db --external-gene-calls jkh125_unicyclerHYBRID_gene_calls.txt -n jkh125_unicyclerHYBRID_prokka_database

anvi-import-functions -c jkh125_unicyclerHYBRID_prokka.db -i jkh125_unicyclerHYBRID_annotation.txt

anvi-gen-contigs-database -f JKH144_SPAdes_reformatted.fasta -o JKH144_SPAdes_prokka.db --external-gene-calls JKH144_SPAdes_gene_calls.txt -n JKH144_SPAdes_prokka_database

anvi-import-functions -c JKH144_SPAdes_prokka.db -i JKH144_SPAdes_annotation.txt

anvi-gen-contigs-database -f JKH144_canu_reformatted.fasta -o JKH144_canu_prokka.db --external-gene-calls JKH144_canu_gene_calls.txt -n JKH144_canu_prokka_database

anvi-import-functions -c JKH144_canu_prokka.db -i JKH144_canu_annotation.txt

anvi-gen-contigs-database -f JKH144_unicycler_reformatted.fasta -o JKH144_unicycler_prokka.db --external-gene-calls JKH144_unicycler_gene_calls.txt -n JKH144_unicycler_prokka_database

anvi-import-functions -c JKH144_unicycler_prokka.db -i JKH144_unicycler_annotation.txt

anvi-gen-contigs-database -f jkh144_nanopolished_reformatted.fasta -o jkh144_nanopolished_prokka.db --external-gene-calls jkh144_nanopolished_gene_calls.txt -n jkh144_nanopolished_prokka_database

anvi-import-functions -c jkh144_nanopolished_prokka.db -i jkh144_nanopolished_annotation.txt

anvi-gen-contigs-database -f jkh144_pilon_reformatted.fasta -o jkh144_pilon_prokka.db --external-gene-calls jkh144_pilon_gene_calls.txt -n jkh144_pilon_prokka_database

anvi-import-functions -c jkh144_pilon_prokka.db -i jkh144_pilon_annotation.txt

anvi-gen-contigs-database -f jkh144_spadesHYBRID_reformatted.fasta -o jkh144_spadesHYBRID_prokka.db --external-gene-calls jkh144_spadesHYBRID_gene_calls.txt -n jkh144_spadesHYBRID_prokka_database

anvi-import-functions -c jkh144_spadesHYBRID_prokka.db -i jkh144_spadesHYBRID_annotation.txt

anvi-gen-contigs-database -f jkh144_unicyclerHYBRID_reformatted.fasta -o jkh144_unicyclerHYBRID_prokka.db --external-gene-calls jkh144_unicyclerHYBRID_gene_calls.txt -n jkh144_unicyclerHYBRID_prokka_database

anvi-import-functions -c jkh144_unicyclerHYBRID_prokka.db -i jkh144_unicyclerHYBRID_annotation.txt

anvi-gen-contigs-database -f jkh158_nanopolished_reformatted.fasta -o jkh158_nanopolished_prokka.db --external-gene-calls jkh158_nanopolished_gene_calls.txt -n jkh158_nanopolished_prokka_database

anvi-import-functions -c jkh158_nanopolished_prokka.db -i jkh158_nanopolished_annotation.txt

anvi-gen-contigs-database -f JKH158_SPAdes_reformatted.fasta -o JKH158_SPAdes_prokka.db --external-gene-calls JKH158_SPAdes_gene_calls.txt -n JKH158_SPAdes_prokka_database

anvi-import-functions -c JKH158_SPAdes_prokka.db -i JKH158_SPAdes_annotation.txt

anvi-gen-contigs-database -f JKH158_canu_reformatted.fasta -o JKH158_canu_prokka.db --external-gene-calls JKH158_canu_gene_calls.txt -n JKH158_canu_prokka_database

anvi-import-functions -c JKH158_canu_prokka.db -i JKH158_canu_annotation.txt

anvi-gen-contigs-database -f JKH158_unicycler_reformatted.fasta -o JKH158_unicycler_prokka.db --external-gene-calls JKH158_unicycler_gene_calls.txt -n JKH158_unicycler_prokka_database

anvi-import-functions -c JKH158_unicycler_prokka.db -i JKH158_unicycler_annotation.txt

anvi-gen-contigs-database -f jkh158_pilon_reformatted.fasta -o jkh158_pilon_prokka.db --external-gene-calls jkh158_pilon_gene_calls.txt -n jkh158_pilon_prokka_database

anvi-import-functions -c jkh158_pilon_prokka.db -i jkh158_pilon_annotation.txt

anvi-gen-contigs-database -f jkh158_spadesHYBRID_reformatted.fasta -o jkh158_spadesHYBRID_prokka.db --external-gene-calls jkh158_spadesHYBRID_gene_calls.txt -n jkh158_spadesHYBRID_prokka_database

anvi-import-functions -c jkh158_spadesHYBRID_prokka.db -i jkh158_spadesHYBRID_annotation.txt

anvi-gen-contigs-database -f jkh158_unicyclerHYBRID_reformatted.fasta -o jkh158_unicyclerHYBRID_prokka.db --external-gene-calls jkh158_unicyclerHYBRID_gene_calls.txt -n jkh158_unicyclerHYBRID_prokka_database

anvi-import-functions -c jkh158_unicyclerHYBRID_prokka.db -i jkh158_unicyclerHYBRID_annotation.txt

# Anvio: running Hmms

anvi-run-hmms -c 1748_SPAdes_prokka.db --num-threads 4

anvi-run-hmms -c 1748_canu_prokka.db --num-threads 4

anvi-run-hmms -c 1748_nanopolish_prokka.db --num-threads 4

anvi-run-hmms -c 1748_pilon_prokka.db --num-threads 4

anvi-run-hmms -c 1748_spades_hybrid_prokka.db --num-threads 4

anvi-run-hmms -c 1748_unicycler_hybrid_prokka.db --num-threads 4

anvi-run-hmms -c 1748_unicycler_prokka.db --num-threads 4

anvi-run-hmms -c 2071_SPAdes_prokka.db --num-threads 4

anvi-run-hmms -c 2071_canu_prokka.db --num-threads 4

anvi-run-hmms -c 2071_nanopolish_prokka.db --num-threads 4

anvi-run-hmms -c 2071_pilon_prokka.db --num-threads 4

anvi-run-hmms -c 2071_spades_hybrid_prokka.db --num-threads 4

anvi-run-hmms -c 2071_unicycler_hybrid_prokka.db --num-threads 4

anvi-run-hmms -c 2071_unicycler_prokka.db --num-threads 4

anvi-run-hmms -c 2345_SPAdes_prokka.db --num-threads 4

anvi-run-hmms -c 2345_canu_prokka.db --num-threads 4

anvi-run-hmms -c 2345_nanopolish_prokka.db --num-threads 4

anvi-run-hmms -c 2345_pilon_prokka.db --num-threads 4

anvi-run-hmms -c 2345_spades_hybrid_prokka.db --num-threads 4

anvi-run-hmms -c 2345_unicycler_hybrid_prokka.db --num-threads 4

anvi-run-hmms -c 2345_unicycler_prokka.db --num-threads 4

anvi-run-hmms -c 2347_SPAdes_prokka.db --num-threads 4

anvi-run-hmms -c 2347_canu_prokka.db --num-threads 4

anvi-run-hmms -c 2347_nanopolish_prokka.db --num-threads 4

anvi-run-hmms -c 2347_pilon_prokka.db --num-threads 4

anvi-run-hmms -c 2347_spades_hybrid_prokka.db --num-threads 4

anvi-run-hmms -c 2347_unicycler_hybrid_prokka.db --num-threads 4

anvi-run-hmms -c 2347_unicycler_prokka.db --num-threads 4

anvi-run-hmms -c 2348_SPAdes_prokka.db --num-threads 4

anvi-run-hmms -c 2348_canu_prokka.db --num-threads 4

anvi-run-hmms -c 2348_nanopolished_prokka.db --num-threads 4

anvi-run-hmms -c 2348_pilon_prokka.db --num-threads 4

anvi-run-hmms -c 2348_spades_hybrid_prokka.db --num-threads 4

anvi-run-hmms -c 2348_unicycler_hybrid_prokka.db --num-threads 4

anvi-run-hmms -c 2348_unicycler_prokka.db --num-threads 4

anvi-run-hmms -c jg3_SPAdes_prokka.db --num-threads 4

anvi-run-hmms -c jg3_canu_prokka.db --num-threads 4

anvi-run-hmms -c jg3_new_nanopolished_prokka.db --num-threads 4

anvi-run-hmms -c jg3_pilon_prokka.db --num-threads 4

anvi-run-hmms -c jg3_spades_hybrid_prokka.db --num-threads 4

anvi-run-hmms -c jg3_unicycler_hybrid_prokka.db --num-threads 4

anvi-run-hmms -c jg3_unicycler_prokka.db --num-threads 4

anvi-run-hmms -c JKH125_SPAdes_prokka.db --num-threads 4

anvi-run-hmms -c JKH125_canu_prokka.db --num-threads 4

anvi-run-hmms -c JKH125_unicycler_prokka.db --num-threads 4

anvi-run-hmms -c jkh125_nanopolished_prokka.db --num-threads 4

anvi-run-hmms -c jkh125_pilon_prokka.db --num-threads 4

anvi-run-hmms -c jkh125_spadesHYBRID_prokka.db --num-threads 4

anvi-run-hmms -c jkh125_unicyclerHYBRID_prokka.db --num-threads 4

anvi-run-hmms -c jkh144_nanopolished_prokka.db --num-threads 4

anvi-run-hmms -c jkh144_pilon_prokka.db --num-threads 4

anvi-run-hmms -c jkh144_spadesHYBRID_prokka.db --num-threads 4

anvi-run-hmms -c jkh144_unicyclerHYBRID_prokka.db --num-threads 4

anvi-run-hmms -c JKH144_canu_prokka.db --num-threads 4

anvi-run-hmms -c JKH144_SPAdes_prokka.db --num-threads 4

anvi-run-hmms -c JKH144_unicycler_prokka.db --num-threads 4

anvi-run-hmms -c jkh158_spadesHYBRID_prokka.db --num-threads 4

anvi-run-hmms -c jkh158_unicyclerHYBRID_prokka.db --num-threads 4

anvi-run-hmms -c JKH158_SPAdes_prokka.db --num-threads 4

anvi-run-hmms -c JKH158_unicycler_prokka.db --num-threads 4

anvi-run-hmms -c jkh158_pilon_prokka.db --num-threads 4

anvi-run-hmms -c jkh158_nanopolished_prokka.db --num-threads 4

anvi-run-hmms -c JKH158_canu_prokka.db --num-threads 4

# Anvio: Generate genome storage database using .txt name files

anvi-gen-genomes-storage -e 1748_anvio_db.txt -o 1748-PROKKA-GENOMES.db --gene-caller Prodigal

anvi-gen-genomes-storage -e 2071_anvio_db.txt -o 2071-PROKKA-GENOMES.db --gene-caller Prodigal

anvi-gen-genomes-storage -e 2345_anvio_db.txt -o 2345-PROKKA-GENOMES.db --gene-caller Prodigal

anvi-gen-genomes-storage -e 2347_anvio_db.txt -o 2347-PROKKA-GENOMES.db --gene-caller Prodigal

anvi-gen-genomes-storage -e 2348_anvio_db.txt -o 2348-PROKKA-GENOMES.db --gene-caller Prodigal

anvi-gen-genomes-storage -e jg3_anvio_db.txt -o jg3-PROKKA-GENOMES.db --gene-caller Prodigal

anvi-gen-genomes-storage -e jkh125_anvio_db.txt -o JKH125-PROKKA-GENOMES.db --gene-caller Prodigal

anvi-gen-genomes-storage -e jkh144_anvio_db.txt -o JKH144-PROKKA-GENOMES.db --gene-caller Prodigal

anvi-gen-genomes-storage -e jkh158_anvio_db.txt -o JKH158-PROKKA-GENOMES.db --gene-caller Prodigal

# Name and path files for each genome:

name contigs_db_path

ah_1748_pilon 1748_pilon_prokka.db

ah_1748_nanopolish 1748_nanopolish_prokka.db

ah_1748_canu 1748_canu_prokka.db

ah_1748_unicycler_hybrid 1748_unicycler_hybrid_prokka.db

ah_1748_unicycler 1748_unicycler_prokka.db

ah_1748_spades_hybrid 1748_spades_hybrid_prokka.db

name contigs_db_path

aer_2071_pilon 2071_pilon_prokka.db

aer_2071_nanopolish 2071_nanopolish_prokka.db

aer_2071_canu 2071_canu_prokka.db

aer_2071_unicycler_hybrid 2071_unicycler_hybrid_prokka.db

aer_2071_unicycler 2071_unicycler_prokka.db

aer_2071_spades_hybrid 2071_spades_hybrid_prokka.db

aer_2071_spades 2071_SPAdes_prokka.db

ah_1748_spades 1748_SPAdes_prokka.db

name contigs_db_path

Fc_2345_pilon 2345_pilon_prokka.db

Fc_2345_nanopolish 2345_nanopolish_prokka.db

Fc_2345_canu 2345_canu_prokka.db

Fc_2345_unicycler_hybrid 2345_unicycler_hybrid_prokka.db

Fc_2345_unicycler 2345_unicycler_prokka.db

Fc_2345_spades_hybrid 2345_spades_hybrid_prokka.db

Fc_2345_spades 2345_SPAdes_prokka.db

name contigs_db_path

Fc_2347_pilon 2347_pilon_prokka.db

Fc_2347_nanopolish 2347_nanopolish_prokka.db

Fc_2347_canu 2347_canu_prokka.db

Fc_2347_unicycler_hybrid 2347_unicycler_hybrid_prokka.db

Fc_2347_unicycler 2347_unicycler_prokka.db

Fc_2347_spades_hybrid 2347_spades_hybrid_prokka.db

Fc_2347_spades 2347_SPAdes_prokka.db

name contigs_db_path

Fc_2348_pilon 2348_pilon_prokka.db

Fc_2348_nanopolish 2348_nanopolished_prokka.db

Fc_2348_canu 2348_canu_prokka.db

Fc_2348_unicycler_hybrid 2348_unicycler_hybrid_prokka.db

Fc_2348_unicycler 2348_unicycler_prokka.db

Fc_2348_spades_hybrid 2348_spades_hybrid_prokka.db

Fc_2348_spades 2348_SPAdes_prokka.db

name contigs_db_path

aer_jg3_pilon jg3_pilon_prokka.db

aer_jg3_nanopolish jg3_new_nanopolished_prokka.db

aer_jg3_canu jg3_canu_prokka.db

aer_jg3_unicycler_hybrid jg3_unicycler_hybrid_prokka.db

aer_jg3_unicycler jg3_unicycler_prokka.db

aer_jg3_spades_hybrid jg3_spades_hybrid_prokka.db

aer_jg3_spades jg3_SPAdes_prokka.db

name contigs_db_path

jkh125_pilon jkh125_pilon_prokka.db

jkh125_nanopolished jkh125_nanopolished_prokka.db

JKH125_canu JKH125_canu_prokka.db

jkh125_unicyclerHYBRID jkh125_unicyclerHYBRID_prokka.db

JKH125_unicycler JKH125_unicycler_prokka.db

jkh125_spadesHYBRID jkh125_spadesHYBRID_prokka.db

JKH125_SPAdes JKH125_SPAdes_prokka.db

name contigs_db_path

jkh144_pilon jkh144_pilon_prokka.db

jkh144_nanopolished jkh144_nanopolished_prokka.db

JKH144_canu JKH144_canu_prokka.db

jkh144_unicyclerHYBRID jkh144_unicyclerHYBRID_prokka.db

JKH144_unicycler JKH144_unicycler_prokka.db

jkh144_spadesHYBRID jkh144_spadesHYBRID_prokka.db

JKH144_SPAdes JKH144_SPAdes_prokka.db

name contigs_db_path

jkh158_pilon jkh158_pilon_prokka.db

jkh158_nanopolished jkh158_nanopolished_prokka.db

JKH158_canu JKH158_canu_prokka.db

jkh158_unicyclerHYBRID jkh158_unicyclerHYBRID_prokka.db

JKH158_unicycler JKH158_unicycler_prokka.db

jkh158_spadesHYBRID jkh158_spadesHYBRID_prokka.db

JKH158_SPAdes JKH158_SPAdes_prokka.db

# Anvio: create Pan-genome

anvi-pan-genome -g 1748-PROKKA-GENOMES.db -n Ah_1748_prokka-pangenome --num-threads 4

anvi-pan-genome -g 2071-PROKKA-GENOMES.db -n Ac_2071_prokka-pangenome --num-threads 4

anvi-pan-genome -g 2345-PROKKA-GENOMES.db -n Fc_2345_prokka-pangenome --num-threads 4

anvi-pan-genome -g 2347-PROKKA-GENOMES.db -n Fc_2347_prokka-pangenome --num-threads 4

anvi-pan-genome -g 2348-PROKKA-GENOMES.db -n Fc_2348_prokka-pangenome --num-threads 4

anvi-pan-genome -g jg3-PROKKA-GENOMES.db -n Av_jg3_prokka-pangenome --num-threads 4

anvi-pan-genome -g JKH125-PROKKA-GENOMES.db -n JKH125_prokka-pangenome --num-threads 4

anvi-pan-genome -g JKH144-PROKKA-GENOMES.db -n JKH144_prokka-pangenome --num-threads 4

anvi-pan-genome -g JKH158-PROKKA-GENOMES.db -n JKH158_prokka-pangenome --num-threads 4

#Anvio: Display pangenome in Chrome

Aeromonas

anvi-display-pan -p Ac_2071_prokka-pangenome-PAN.db -g ../2071-PROKKA-GENOMES.db

anvi-display-pan -p Ah_1748_prokka-pangenome-PAN.db -g ../1748-PROKKA-GENOMES.db

anvi-display-pan -p Av_jg3_prokka-pangenome-PAN.db -g ../jg3-PROKKA-GENOMES.db

Flavobacterium

anvi-display-pan -p Fc_2345_prokka-pangenome-PAN.db -g ../2345-PROKKA-GENOMES.db

anvi-display-pan -p Fc_2347_prokka-pangenome-PAN.db -g ../2347-PROKKA-GENOMES.db

anvi-display-pan -p Fc_2348_prokka-pangenome-PAN.db -g ../2348-PROKKA-GENOMES.db

Pseudonocardia

anvi-display-pan -p JKH125_prokka-pangenome-PAN.db -g ../JKH125-PROKKA-GENOMES.db

anvi-display-pan -p JKH158_prokka-pangenome-PAN.db -g ../JKH158-PROKKA-GENOMES.db

anvi-display-pan -p JKH144_prokka-pangenome-PAN.db -g ../JKH144-PROKKA-GENOMES.db

### The three Pseudonocardia genomes did not display in Chrome. Instead, the following terminal error printed:

File "/usr/local/bin/anvi-display-pan", line 72, in <module>

d = interactive.Interactive(args)

File "/usr/local/Cellar/anvio/5.2/libexec/lib/python3.7/site-packages/anvio/interactive.py", line 128, in __init__

self.layers_additional_data_keys, self.layers_additional_data_dict = TableForLayerAdditionalData(self.args).get_all() if a_db_is_found else ([], {})

File "/usr/local/Cellar/anvio/5.2/libexec/lib/python3.7/site-packages/anvio/tables/miscdata.py", line 723, in get_all

keys_dict[group_name], data_dict[group_name] = self.get()

File "/usr/local/Cellar/anvio/5.2/libexec/lib/python3.7/site-packages/anvio/tables/miscdata.py", line 598, in get

d[additional_data_item_name][key] = eval(entry['data_type'])(value or self.nulls_per_type[entry['data_type']])

ValueError: invalid literal for int() with base 10: '71.9424460431655'

According to MerenLab, this is a glitch and will be resolved in a new release. To get around this error, they advise users to use the following commands in terminal which corrected the display error for the three genomes:

$ sqlite3 JKH125_prokka-pangenome-PAN.db "UPDATE layer_additional_data SET data_type='float' WHERE data_key LIKE 'percent_completion'"

sqlite3 JKH125_prokka-pangenome-PAN.db "UPDATE layer_additional_data SET data_type='float' WHERE data_key LIKE 'percent_redundancy'"

anvi-display-pan -p JKH125_prokka-pangenome-PAN.db -g ../JKH125-PROKKA-GENOMES.db

$ sqlite3 JKH158_prokka-pangenome-PAN.db "UPDATE layer_additional_data SET data_type='float' WHERE data_key LIKE 'percent_completion'"

PSMMSAltop1sMBP:JKH158_prokka-pangenome psm_msa_admin$ sqlite3 JKH158_prokka-pangenome-PAN.db "UPDATE layer_additional_data SET data_type='float' WHERE data_key LIKE 'percent_redundancy'"

anvi-display-pan -p JKH158_prokka-pangenome-PAN.db -g ../JKH158-PROKKA-GENOMES.db

$ sqlite3 JKH144_prokka-pangenome-PAN.db "UPDATE layer_additional_data SET data_type='float' WHERE data_key LIKE 'percent_completion'"

PSMMSAltop1sMBP:JKH144_prokka-pangenome psm_msa_admin$ sqlite3 JKH144_prokka-pangenome-PAN.db "UPDATE layer_additional_data SET data_type='float' WHERE data_key LIKE 'percent_redundancy'"

anvi-display-pan -p JKH144_prokka-pangenome-PAN.db -g ../JKH144-PROKKA-GENOMES.db

Commands for data in Figure 5

# Fore each genome, the number of fast5 files needed to achieve the desired coverage (based on overage read length) was calculated and then the appropriate number of folders were used to generate fastq files using poretools.

# JKH158 subsampling

# 10X

porechop -i JKH158_JKS2128/JKH158_1D_coverage_10X.fastq.gz -o porechopped/JKH158_1D_coverage_10X_porechopped.fastq.gz --discard_middle > porechopped/JKH158_1D_coverage_10X_porechop.log

zcat porechopped/JKH158_1D_coverage_10X_porechopped.fastq.gz | python3 ~/Tools/nanofilt-1.0.5/nanofilt/NanoFilt.py -q 9 -l 500 | gzip > nanofilt_porechopped/JKH158_1D_coverage_10X_nanofilt_porechopped.fastq.gz

~/Tools/canu-1.5/Linux-amd64/bin/canu -p JKH158_1D_coverage_10X -d canu/JKH158_1D_coverage_10X -genomeSize=6.5m -nanopore-raw nanofilt_porechopped/JKH158_1D_coverage_10X_nanofilt_porechopped.fastq.gz gnuplotTested=true stopOnReadQuality=false > canu/JKH158_1D_coverage_10X_canu.log

# 20X

porechop -i JKH158_JKS2128/JKH158_1D_coverage_20X.fastq.gz -o porechopped/JKH158_1D_coverage_20X_porechopped.fastq.gz --discard_middle > porechopped/JKH158_1D_coverage_20X_porechop.log

zcat porechopped/JKH158_1D_coverage_20X_porechopped.fastq.gz | python3 ~/Tools/nanofilt-1.0.5/nanofilt/NanoFilt.py -q 9 -l 500 | gzip > nanofilt_porechopped/JKH158_1D_coverage_20X_nanofilt_porechopped.fastq.gz

~/Tools/canu-1.5/Linux-amd64/bin/canu -p JKH158_1D_coverage_20X -d canu/JKH158_1D_coverage_20X -genomeSize=6.5m -nanopore-raw nanofilt_porechopped/JKH158_1D_coverage_20X_nanofilt_porechopped.fastq.gz gnuplotTested=true stopOnReadQuality=false > canu/JKH158_1D_coverage_20X_canu.log

# 30X

porechop -i JKH158_JKS2128/JKH158_1D_coverage_30X.fastq.gz -o porechopped/JKH158_1D_coverage_30X_porechopped.fastq.gz --discard_middle > porechopped/JKH158_1D_coverage_30X_porechop.log

zcat porechopped/JKH158_1D_coverage_30X_porechopped.fastq.gz | python3 ~/Tools/nanofilt-1.0.5/nanofilt/NanoFilt.py -q 9 -l 500 | gzip > nanofilt_porechopped/JKH158_1D_coverage_30X_nanofilt_porechopped.fastq.gz

~/Tools/canu-1.5/Linux-amd64/bin/canu -p JKH158_1D_coverage_30X -d canu/JKH158_1D_coverage_30X -genomeSize=6.5m -nanopore-raw nanofilt_porechopped/JKH158_1D_coverage_30X_nanofilt_porechopped.fastq.gz gnuplotTested=true stopOnReadQuality=false > canu/JKH158_1D_coverage_30X_canu.log

# 40X

porechop -i JKH158_JKS2128/JKH158_1D_coverage_40X.fastq.gz -o porechopped/JKH158_1D_coverage_40X_porechopped.fastq.gz --discard_middle > porechopped/JKH158_1D_coverage_40X_porechop.log

zcat porechopped/JKH158_1D_coverage_40X_porechopped.fastq.gz | python3 ~/Tools/nanofilt-1.0.5/nanofilt/NanoFilt.py -q 9 -l 500 | gzip > nanofilt_porechopped/JKH158_1D_coverage_40X_nanofilt_porechopped.fastq.gz

~/Tools/canu-1.5/Linux-amd64/bin/canu -p JKH158_1D_coverage_40X -d canu/JKH158_1D_coverage_40X -genomeSize=6.5m -nanopore-raw nanofilt_porechopped/JKH158_1D_coverage_40X_nanofilt_porechopped.fastq.gz gnuplotTested=true stopOnReadQuality=false > canu/JKH158_1D_coverage_40X_canu.log

# 50X

porechop -i JKH158_JKS2128/JKH158_1D_coverage_50X.fastq.gz -o porechopped/JKH158_1D_coverage_50X_porechopped.fastq.gz --discard_middle > porechopped/JKH158_1D_coverage_50X_porechop.log

zcat porechopped/JKH158_1D_coverage_50X_porechopped.fastq.gz | python3 ~/Tools/nanofilt-1.0.5/nanofilt/NanoFilt.py -q 9 -l 500 | gzip > nanofilt_porechopped/JKH158_1D_coverage_50X_nanofilt_porechopped.fastq.gz

~/Tools/canu-1.5/Linux-amd64/bin/canu -p JKH158_1D_coverage_50X -d canu/JKH158_1D_coverage_50X -genomeSize=6.5m -nanopore-raw nanofilt_porechopped/JKH158_1D_coverage_50X_nanofilt_porechopped.fastq.gz gnuplotTested=true stopOnReadQuality=false > canu/JKH158_1D_coverage_50X_canu.log

# 60X

porechop -i JKH158_JKS2128/JKH158_1D_coverage_60X.fastq.gz -o porechopped/JKH158_1D_coverage_60X_porechopped.fastq.gz --discard_middle > porechopped/JKH158_1D_coverage_60X_porechop.log

zcat porechopped/JKH158_1D_coverage_60X_porechopped.fastq.gz | python3 ~/Tools/nanofilt-1.0.5/nanofilt/NanoFilt.py -q 9 -l 500 | gzip > nanofilt_porechopped/JKH158_1D_coverage_60X_nanofilt_porechopped.fastq.gz

~/Tools/canu-1.5/Linux-amd64/bin/canu -p JKH158_1D_coverage_60X -d canu/JKH158_1D_coverage_60X -genomeSize=6.5m -nanopore-raw nanofilt_porechopped/JKH158_1D_coverage_60X_nanofilt_porechopped.fastq.gz gnuplotTested=true stopOnReadQuality=false > canu/JKH158_1D_coverage_60X_canu.log

# 70X

porechop -i JKH158_JKS2128/JKH158_1D_coverage_70X.fastq.gz -o porechopped/JKH158_1D_coverage_70X_porechopped.fastq.gz --discard_middle > porechopped/JKH158_1D_coverage_70X_porechop.log

zcat porechopped/JKH158_1D_coverage_70X_porechopped.fastq.gz | python3 ~/Tools/nanofilt-1.0.5/nanofilt/NanoFilt.py -q 9 -l 500 | gzip > nanofilt_porechopped/JKH158_1D_coverage_70X_nanofilt_porechopped.fastq.gz

~/Tools/canu-1.5/Linux-amd64/bin/canu -p JKH158_1D_coverage_70X -d canu/JKH158_1D_coverage_70X -genomeSize=6.5m -nanopore-raw nanofilt_porechopped/JKH158_1D_coverage_70X_nanofilt_porechopped.fastq.gz gnuplotTested=true stopOnReadQuality=false > canu/JKH158_1D_coverage_70X_canu.log

# QUAST

~/Tools/quast-4.6.3/quast.py JKH158_70X_canu.fasta -o JKH158_70X_canu_QUAST

~/Tools/quast-4.6.3/quast.py JKH158_60X_canu.fasta -o JKH158_60X_canu_QUAST

~/Tools/quast-4.6.3/quast.py JKH158_50X_canu.fasta -o JKH158_50X_canu_QUAST

~/Tools/quast-4.6.3/quast.py JKH158_40X_canu.fasta -o JKH158_40X_canu_QUAST

~/Tools/quast-4.6.3/quast.py JKH158_40X_canu.fasta -o JKH158_30X_canu_QUAST

~/Tools/quast-4.6.3/quast.py JKH158_20X_canu.fasta -o JKH158_20X_canu_QUAST

~/Tools/quast-4.6.3/quast.py JKH158_10X_canu.fasta -o JKH158_10X_canu_QUAST

~/Tools/quast-4.6.3/quast.py jg3_10X_canu.fasta -o jg3_10X_canu_QUAST

~/Tools/quast-4.6.3/quast.py jg3_20X_canu.fasta -o jg3_20X_canu_QUAST

~/Tools/quast-4.6.3/quast.py jg3_30X_canu.fasta -o jg3_30X_canu_QUAST

~/Tools/quast-4.6.3/quast.py jg3_40X_canu.fasta -o jg3_40X_canu_QUAST

~/Tools/quast-4.6.3/quast.py 2345_10X_canu.fasta -o 2345_10X_canu_QUAST

~/Tools/quast-4.6.3/quast.py 2345_20X_canu.fasta -o 2345_20X_canu_QUAST

~/Tools/quast-4.6.3/quast.py 2345_30X_canu.fasta -o 2345_30X_canu_QUAST

~/Tools/quast-4.6.3/quast.py 2345_40X_canu.fasta -o 2345_40X_canu_QUAST

~/Tools/quast-4.6.3/quast.py 2345_50X_canu.fasta -o 2345_50X_canu_QUAST

~/Tools/quast-4.6.3/quast.py 2345_60X_canu.fasta -o 2345_60X_canu_QUAST

# 2345 subsampling

# 10X

porechop -i 2345/2345_10X.fastq.gz -o porechopped/2345_10X_porechopped.fastq.gz --discard_middle > porechopped/2345_10X_porechop.log

zcat porechopped/2345_10X_porechopped.fastq.gz | python3 ~/Tools/nanofilt-1.0.5/nanofilt/NanoFilt.py -q 9 -l 500 | gzip > nanofilt_porechopped/2345_10X_nanofilt_porechopped.fastq.gz

~/Tools/canu-1.5/Linux-amd64/bin/canu -p 2345_10X -d canu/2345_10X -genomeSize=3.2m -nanopore-raw nanofilt_porechopped/2345_10X_nanofilt_porechopped.fastq.gz gnuplotTested=true stopOnReadQuality=false > canu/2345_10X_canu.log

# 20X

porechop -i 2345/2345_20X.fastq.gz -o porechopped/2345_20X_porechopped.fastq.gz --discard_middle > porechopped/2345_20X_porechop.log

zcat porechopped/2345_20X_porechopped.fastq.gz | python3 ~/Tools/nanofilt-1.0.5/nanofilt/NanoFilt.py -q 9 -l 500 | gzip > nanofilt_porechopped/2345_20X_nanofilt_porechopped.fastq.gz

~/Tools/canu-1.5/Linux-amd64/bin/canu -p 2345_20X -d canu/2345_20X -genomeSize=3.2m -nanopore-raw nanofilt_porechopped/2345_20X_nanofilt_porechopped.fastq.gz gnuplotTested=true stopOnReadQuality=false > canu/2345_20X_canu.log

# 30X

porechop -i 2345/2345_30X.fastq.gz -o porechopped/2345_30X_porechopped.fastq.gz --discard_middle > porechopped/2345_30X_porechop.log

zcat porechopped/2345_30X_porechopped.fastq.gz | python3 ~/Tools/nanofilt-1.0.5/nanofilt/NanoFilt.py -q 9 -l 500 | gzip > nanofilt_porechopped/2345_30X_nanofilt_porechopped.fastq.gz

~/Tools/canu-1.5/Linux-amd64/bin/canu -p 2345_30X -d canu/2345_30X -genomeSize=3.2m -nanopore-raw nanofilt_porechopped/2345_30X_nanofilt_porechopped.fastq.gz gnuplotTested=true stopOnReadQuality=false > canu/2345_30X_canu.log

# 40X

porechop -i 2345/2345_40X.fastq.gz -o porechopped/2345_40X_porechopped.fastq.gz --discard_middle > porechopped/2345_40X_porechop.log

zcat porechopped/2345_40X_porechopped.fastq.gz | python3 ~/Tools/nanofilt-1.0.5/nanofilt/NanoFilt.py -q 9 -l 500 | gzip > nanofilt_porechopped/2345_40X_nanofilt_porechopped.fastq.gz

~/Tools/canu-1.5/Linux-amd64/bin/canu -p 2345_40X -d canu/2345_40X -genomeSize=3.2m -nanopore-raw nanofilt_porechopped/2345_40X_nanofilt_porechopped.fastq.gz gnuplotTested=true stopOnReadQuality=false > canu/2345_40X_canu.log

# 50X

porechop -i 2345/2345_50X.fastq.gz -o porechopped/2345_50X_porechopped.fastq.gz --discard_middle > porechopped/2345_50X_porechop.log

zcat porechopped/2345_50X_porechopped.fastq.gz | python3 ~/Tools/nanofilt-1.0.5/nanofilt/NanoFilt.py -q 9 -l 500 | gzip > nanofilt_porechopped/2345_50X_nanofilt_porechopped.fastq.gz

~/Tools/canu-1.5/Linux-amd64/bin/canu -p 2345_50X -d canu/2345_50X -genomeSize=3.2m -nanopore-raw nanofilt_porechopped/2345_50X_nanofilt_porechopped.fastq.gz gnuplotTested=true stopOnReadQuality=false > canu/2345_50X_canu.log

# 60X

porechop -i 2345/2345_60X.fastq.gz -o porechopped/2345_60X_porechopped.fastq.gz --discard_middle > porechopped/2345_60X_porechop.log

zcat porechopped/2345_60X_porechopped.fastq.gz | python3 ~/Tools/nanofilt-1.0.5/nanofilt/NanoFilt.py -q 9 -l 500 | gzip > nanofilt_porechopped/2345_60X_nanofilt_porechopped.fastq.gz

~/Tools/canu-1.5/Linux-amd64/bin/canu -p 2345_60X -d canu/2345_60X -genomeSize=3.2m -nanopore-raw nanofilt_porechopped/2345_60X_nanofilt_porechopped.fastq.gz gnuplotTested=true stopOnReadQuality=false > canu/2345_60X_canu.log

# 70X

porechop -i 2345/2345_70X.fastq.gz -o porechopped/2345_70X_porechopped.fastq.gz --discard_middle > porechopped/2345_70X_porechop.log

zcat porechopped/2345_70X_porechopped.fastq.gz | python3 ~/Tools/nanofilt-1.0.5/nanofilt/NanoFilt.py -q 9 -l 500 | gzip > nanofilt_porechopped/2345_70X_nanofilt_porechopped.fastq.gz

~/Tools/canu-1.5/Linux-amd64/bin/canu -p 2345_70X -d canu/2345_70X -genomeSize=3.2m -nanopore-raw nanofilt_porechopped/2345_70X_nanofilt_porechopped.fastq.gz gnuplotTested=true stopOnReadQuality=false > canu/2345_70X_canu.log

SNPs and Indels for Coverage Comparison

JKH158 10X

nucmer jkh158_pilon.fasta JKH158_1D_coverage_10X_canu.fasta -p JKH158_10X_vs_All

delta-filter -1 JKH158_10X_vs_All.delta > JKH158_10X_All.delta.filtered.delta

show-snps JKH158_10X_vs_All.delta.filtered.delta > JKH158_10X_vs_All.delta.filtered.snps

perl count_SNPS_indels.pl JKH158_10X_vs_All.delta.filtered.snps JKH158_10X_vs_All.counts.txt

JKH158 20X

nucmer jkh158_pilon.fasta JKH158_1D_coverage_20X_canu.fasta -p JKH158_20X_vs_All

delta-filter -1 JKH158_20X_vs_All.delta > JKH158_20X_All.delta.filtered.delta

show-snps JKH158_20X_vs_All.delta.filtered.delta > JKH158_20X_vs_All.delta.filtered.snps

perl count_SNPS_indels.pl JKH158_20X_vs_All.delta.filtered.snps JKH158_20X_vs_All.counts.txt

JKH158 30X

nucmer jkh158_pilon.fasta JKH158_1D_coverage_30X_canu.fasta -p JKH158_30X_vs_All

delta-filter -1 JKH158_30X_vs_All.delta > JKH158_30X_All.delta.filtered.delta

show-snps JKH158_30X_vs_All.delta.filtered.delta > JKH158_30X_vs_All.delta.filtered.snps

perl count_SNPS_indels.pl JKH158_30X_vs_All.delta.filtered.snps JKH158_30X_vs_All.counts.txt

JKH158 40X

nucmer jkh158_pilon.fasta JKH158_1D_coverage_40X_canu.fasta -p JKH158_40X_vs_All

delta-filter -1 JKH158_40X_vs_All.delta > JKH158_40X_All.delta.filtered.delta

show-snps JKH158_40X_vs_All.delta.filtered.delta > JKH158_40X_vs_All.delta.filtered.snps

perl count_SNPS_indels.pl JKH158_40X_vs_All.delta.filtered.snps JKH158_40X_vs_All.counts.txt

JKH158 50X

nucmer jkh158_pilon.fasta JKH158_1D_coverage_50X_canu.fasta -p JKH158_50X_vs_All

delta-filter -1 JKH158_50X_vs_All.delta > JKH158_50X_All.delta.filtered.delta

show-snps JKH158_50X_vs_All.delta.filtered.delta > JKH158_50X_vs_All.delta.filtered.snps

perl count_SNPS_indels.pl JKH158_50X_vs_All.delta.filtered.snps JKH158_50X_vs_All.counts.txt

JKH158 60X

nucmer jkh158_pilon.fasta JKH158_1D_coverage_60X_canu.fasta -p JKH158_60X_vs_All

delta-filter -1 JKH158_60X_vs_All.delta > JKH158_60X_All.delta.filtered.delta

show-snps JKH158_60X_vs_All.delta.filtered.delta > JKH158_60X_vs_All.delta.filtered.snps

perl count_SNPS_indels.pl JKH158_60X_vs_All.delta.filtered.snps JKH158_60X_vs_All.counts.txt

JKH158 70X

nucmer jkh158_pilon.fasta JKH158_1D_coverage_70X_canu.fasta -p JKH158_70X_vs_All

delta-filter -1 JKH158_70X_vs_All.delta > JKH158_70X_All.delta.filtered.delta

show-snps JKH158_70X_vs_All.delta.filtered.delta > JKH158_70X_vs_All.delta.filtered.snps

perl count_SNPS_indels.pl JKH158_70X_vs_All.delta.filtered.snps JKH158_70X_vs_All.counts.txt

JG3 10X

nucmer jg3_pilon_new.fasta jg3_10X_canu.fasta -p jg3_10X_vs_All

delta-filter -1 jg3_10X_vs_All.delta > jg3_10X_vs_All.filtered.delta

show-snps jg3_10X_vs_All.delta.filtered.delta > jg3_10X_vs_All.delta.filtered.snps

perl count_SNPS_indels.pl jg3_10X_vs_All.delta.filtered.snps jg3_10X_vs_All.counts.txt

JG3 20X

nucmer jg3_pilon_new.fasta jg3_20X_canu.fasta -p jg3_20X_vs_All

delta-filter -1 jg3_20X_vs_All.delta > jg3_20X_vs_All.filtered.delta

show-snps jg3_20X_vs_All.delta.filtered.delta > jg3_20X_vs_All.delta.filtered.snps

perl count_SNPS_indels.pl jg3_20X_vs_All.delta.filtered.snps jg3_20X_vs_All.counts.txt

JG3 30X

nucmer jg3_pilon_new.fasta jg3_30X_canu.fasta -p jg3_30X_vs_All

delta-filter -1 jg3_30X_vs_All.delta > jg3_30X_vs_All.filtered.delta

show-snps jg3_30X_vs_All.delta.filtered.delta > jg3_30X_vs_All.delta.filtered.snps

perl count_SNPS_indels.pl jg3_30X_vs_All.delta.filtered.snps jg3_30X_vs_All.counts.txt

JG3 40X

nucmer jg3_pilon_new.fasta jg3_40X_canu.fasta -p jg3_40X_vs_All

delta-filter -1 jg3_40X_vs_All.delta > jg3_40X_vs_All.filtered.delta

show-snps jg3_40X_vs_All.delta.filtered.delta > jg3_40X_vs_All.delta.filtered.snps

perl count_SNPS_indels.pl jg3_40X_vs_All.delta.filtered.snps jg3_40X_vs_All.counts.txt

FS ARS-166-14 10X

nucmer 2345_pilon.fasta 2345_10X_porechopped.fastq -p 2345_10X_vs_All

delta-filter -1 2345_10X_vs_All.delta > 2345_10X_vs_All.filtered.delta

show-snps 2345_10X_vs_All.delta.filtered.delta > 2345_10X_vs_All.delta.filtered.snps

perl count_SNPS_indels.pl 2345_10X_vs_All.delta.filtered.snps 2345_10X_vs_All.counts.txt

FS ARS-166-14 20X

nucmer 2345_pilon.fasta 2345_20X_porechopped.fastq -p 2345_20X_vs_All

delta-filter -1 2345_20X_vs_All.delta > 2345_20X_vs_All.filtered.delta

show-snps 2345_20X_vs_All.delta.filtered.delta > 2345_20X_vs_All.delta.filtered.snps

perl count_SNPS_indels.pl 2345_20X_vs_All.delta.filtered.snps 2345_20X_vs_All.counts.tx

FS ARS-166-14 30X

nucmer 2345_pilon.fasta 2345_30X_porechopped.fastq -p 2345_30X_vs_All

delta-filter -1 2345_30X_vs_All.delta > 2345_30X_vs_All.filtered.delta

show-snps 2345_30X_vs_All.delta.filtered.delta > 2345_30X_vs_All.delta.filtered.snps

perl count_SNPS_indels.pl 2345_30X_vs_All.delta.filtered.snps 2345_30X_vs_All.counts.txt

FS ARS-166-14 40X

nucmer 2345_pilon.fasta 2345_40X_porechopped.fastq -p 2345_40X_vs_All

delta-filter -1 2345_40X_vs_All.delta > 2345_40X_vs_All.filtered.delta

show-snps 2345_40X_vs_All.delta.filtered.delta > 2345_40X_vs_All.delta.filtered.snps

perl count_SNPS_indels.pl 2345_40X_vs_All.delta.filtered.snps 2345_40X_vs_All.counts.txt

FS ARS-166-14 50X

nucmer 2345_pilon.fasta 2345_50X_porechopped.fastq -p 2345_50X_vs_All

delta-filter -1 2345_50X_vs_All.delta > 2345_50X_vs_All.filtered.delta

show-snps 2345_50X_vs_All.delta.filtered.delta > 2345_50X_vs_All.delta.filtered.snps

perl count_SNPS_indels.pl 2345_50X_vs_All.delta.filtered.snps 2345_50X_vs_All.counts.txt

Commands for data in Figure 6

# annotated each genome using prokka

prokka ../../completed_assemblies/pilon/jkh158_pilon.fasta --outdir JKH158_Pilon_annotation --prefix JKH158_Pilon

prokka ../../completed_assemblies/nanopolish/jkh158_nanopolished.fasta --outdir JKH158_Nanopolish_annotation --prefix JKH158_NanoP

prokka ../../completed_assemblies/canu/JKH158_canu.fasta --outdir JKH158_Canu_annotation --prefix JKH158_Canu

prokka ../../completed_assemblies/unicycler/JKH158_unicycler.fasta --outdir JKH158_Unicycler_TruSeq_annotation --prefix JKH158_UniTru

prokka ../../completed_assemblies/unicycler_hybrid/jkh158_unicyclerHYBRID.fasta --outdir JKH158_Unicycler__Hybrid_annotation --prefix JKH158_UniH

prokka ../../completed_assemblies/spades/JKH158_SPAdes.fasta --center XXX --outdir JKH158_SPAdes_annotation --prefix JKH158_SPATru

prokka ../../completed_assemblies/spades_hybrid/jkh158_spadesHYBRID.fasta --center XXX --outdir JKH158_SPAdes_Hybrid_annotation --prefix JKH158_SPAH

# copy gbk files to ClusterCompare Data/genomes directory

bash antiSMASH_annotation.sh

bash cluster_pfam_BBH_comparison.sh

bash find_cluster_completeness.sh

# make a mashtree of all clusters

# copy fnas from BGC_fragmentation (except genomes)

cp ../ClustCompare-master/ClustCompare/Data/BGC_fragmentation/*.fna .

mash sketch -o clusters *.fna

mash info clusters.msh

mash dist clusters.msh *.fna > clusters_comparison.table

~/Tools/mashtree-master/bin/mashtree *.fna > clusters_mashtree.out

# multiple fasta files of clusters in each genome for alignment

cat cluster1.fna cluster2.fna cluster3.fna cluster4.fna cluster5.fna cluster6.fna cluster7.fna cluster8.fna cluster9.fna cluster10.fna cluster11.fna cluster12.fna cluster13.fna cluster14.fna cluster15.fna cluster16.fna cluster17.fna > JKH158_canu_clusters.fna

cat cluster18.fna cluster19.fna cluster20.fna cluster21.fna cluster22.fna cluster23.fna cluster24.fna cluster25.fna cluster26.fna cluster27.fna cluster28.fna cluster29.fna cluster30.fna cluster31.fna cluster32.fna cluster33.fna cluster34.fna cluster35.fna cluster36.fna > JKH158_nanopolish_clusters.fna

cat cluster37.fna cluster38.fna cluster39.fna cluster40.fna cluster41.fna cluster42.fna cluster43.fna cluster44.fna cluster45.fna cluster46.fna cluster47.fna cluster48.fna cluster49.fna cluster50.fna cluster51.fna cluster52.fna cluster53.fna cluster54.fna > JKH158_pilon_clusters.fna

cat cluster55.fna cluster56.fna cluster57.fna cluster58.fna cluster59.fna cluster60.fna cluster61.fna cluster62.fna cluster63.fna cluster64.fna cluster65.fna cluster66.fna > JKH158_SPAdesHybrid_clusters.fna

cat cluster67.fna cluster68.fna cluster69.fna cluster70.fna cluster71.fna cluster72.fna cluster73.fna cluster74.fna cluster75.fna cluster76.fna cluster77.fna cluster78.fna > JKH158_SPAdes_clusters.fna

cat cluster79.fna cluster80.fna cluster81.fna cluster82.fna cluster83.fna cluster84.fna cluster85.fna cluster86.fna cluster87.fna cluster88.fna cluster89.fna cluster90.fna > JKH158_UnicyclerHybrid_clusters.fna

cat cluster91.fna cluster92.fna cluster93.fna cluster94.fna cluster95.fna cluster96.fna cluster97.fna cluster98.fna cluster99.fna cluster100.fna cluster101.fna cluster102.fna cluster103.fna > JKH158_Unicycler_clusters.fna

# manually added cluster #s to fasta headers

# aligned all clusters to each other

nucmer JKH158_pilon_clusters.fna JKH158_canu_clusters.fna -p JKH158_pilon_vs_canu

delta-filter -1 JKH158_pilon_vs_canu.delta > JKH158_pilon_vs_canu.delta.filtered.delta

show-coords JKH158_pilon_vs_canu.delta.filtered.delta > JKH158_pilon_vs_canu.delta.filtered.coords

nucmer JKH158_pilon_clusters.fna JKH158_nanopolish_clusters.fna -p JKH158_pilon_vs_nanopolish

delta-filter -1 JKH158_pilon_vs_nanopolish.delta > JKH158_pilon_vs_nanopolish.delta.filtered.delta

show-coords JKH158_pilon_vs_nanopolish.delta.filtered.delta > JKH158_pilon_vs_nanopolish.delta.filtered.coords

nucmer JKH158_pilon_clusters.fna JKH158_SPAdes_clusters.fna -p JKH158_pilon_vs_SPAdes

delta-filter -1 JKH158_pilon_vs_SPAdes.delta > JKH158_pilon_vs_SPAdes.delta.filtered.delta

show-coords JKH158_pilon_vs_SPAdes.delta.filtered.delta > JKH158_pilon_vs_SPAdes.delta.filtered.coords

nucmer JKH158_pilon_clusters.fna JKH158_SPAdesHybrid_clusters.fna -p JKH158_pilon_vs_SPAdesHybrid

delta-filter -1 JKH158_pilon_vs_SPAdesHybrid.delta > JKH158_pilon_vs_SPAdesHybrid.delta.filtered.delta

show-coords JKH158_pilon_vs_SPAdesHybrid.delta.filtered.delta > JKH158_pilon_vs_SPAdesHybrid.delta.filtered.coords

nucmer JKH158_pilon_clusters.fna JKH158_Unicycler_clusters.fna -p JKH158_pilon_vs_Unicycler

delta-filter -1 JKH158_pilon_vs_Unicycler.delta > JKH158_pilon_vs_Unicycler.delta.filtered.delta

show-coords JKH158_pilon_vs_Unicycler.delta.filtered.delta > JKH158_pilon_vs_Unicycler.delta.filtered.coords

nucmer JKH158_pilon_clusters.fna JKH158_UnicyclerHybrid_clusters.fna -p JKH158_pilon_vs_UnicyclerHybrid

delta-filter -1 JKH158_pilon_vs_UnicyclerHybrid.delta > JKH158_pilon_vs_UnicyclerHybrid.delta.filtered.delta

show-coords JKH158_pilon_vs_UnicyclerHybrid.delta.filtered.delta > JKH158_pilon_vs_UnicyclerHybrid.delta.filtered.coords

nucmer JKH158_Unicycler_clusters.fna JKH158_canu_clusters.fna -p JKH158_Unicycler_vs_canu

delta-filter -1 JKH158_Unicycler_vs_canu.delta > JKH158_Unicycler_vs_canu.delta.filtered.delta

show-coords JKH158_Unicycler_vs_canu.delta.filtered.delta > JKH158_Unicycler_vs_canu.delta.filtered.coords

nucmer JKH158_Unicycler_clusters.fna JKH158_nanopolish_clusters.fna -p JKH158_Unicycler_vs_nanopolish

delta-filter -1 JKH158_Unicycler_vs_nanopolish.delta > JKH158_Unicycler_vs_nanopolish.delta.filtered.delta

show-coords JKH158_Unicycler_vs_nanopolish.delta.filtered.delta > JKH158_Unicycler_vs_nanopolish.delta.filtered.coords

nucmer JKH158_nanopolish_clusters.fna JKH158_canu_clusters.fna -p JKH158_nanopolish_vs_canu

delta-filter -1 JKH158_nanopolish_vs_canu.delta > JKH158_nanopolish_vs_canu.delta.filtered.delta

show-coords JKH158_nanopolish_vs_canu.delta.filtered.delta > JKH158_nanopolish_vs_canu.delta.filtered.coords

nucmer JKH158_SPAdesHybrid_clusters.fna JKH158_UnicyclerHybrid_clusters.fna -p JKH158_SPAdesHybrid_vs_UnicyclerHybrid

delta-filter -1 JKH158_SPAdesHybrid_vs_UnicyclerHybrid.delta > JKH158_SPAdesHybrid_vs_UnicyclerHybrid.delta.filtered.delta

show-coords JKH158_SPAdesHybrid_vs_UnicyclerHybrid.delta.filtered.delta > JKH158_SPAdesHybrid_vs_UnicyclerHybrid.delta.filtered.coords

Commands for S1 data

Commands for *Pseudonocardia* Nextera assemblies

# nextera spades assemblies

spades.py --pe1-1 JKH125_Nextera_output_R1_paired.fastq --pe1-2 JKH125_Nextera_output_R2_paired.fastq -o JKH125_SPAdes_Nextera

spades.py --pe1-1 JKH125_Nextera_output_R1_paired.fastq --pe1-2 JKH125_Nextera_output_R2_paired.fastq --nanopore JKH125_all_minION_reads_nanofilt_porechopped.fastq.gz -o JKH125_SPAdes_Hybrid_Nextera

# nexera unicycler assemblies

unicycler -1 JKH125_Nextera_output_R1_paired.fastq -2 JKH125_Nextera_output_R2_paired.fastq -o JKH125_Unicycler_Nextera

unicycler -1 JKH125_Nextera_output_R1_paired.fastq -2 JKH125_Nextera_output_R2_paired.fastq -l JKH125_all_minION_reads_nanofilt_porechopped.fastq.gz -o JKH125_Unicycler_Hybrid_Nextera

# nextera spades assemblies

spades.py --pe1-1 JKH144_nextera_output_R1_paired.fastq --pe1-2 JKH144_nextera_output_R2_paired.fastq -o JKH144_SPAdes_Nextera

spades.py --pe1-1 JKH144_Nextera_output_R1_paired.fastq --pe1-2 JKH144_Nextera_output_R2_paired.fastq --nanopore JKH144_1D_nanofilt_porechopped.fastq.gz -o JKH144_SPAdes_Hybrid_Nextera

# nexera unicycler assemblies

unicycler -1 JKH144_nextera_output_R1_paired.fastq -2 JKH144_nextera_output_R2_paired.fastq -o JKH144_Unicycler_Nextera

unicycler -1 JKH144_nextera_output_R1_paired.fastq -2 JKH144_nextera_output_R2_paired.fastq -l JKH144_1D_nanofilt_porechopped.fastq.gz -o JKH144_Unicycler_Hybrid_Nextera

# nextera spades assemblies

spades.py --pe1-1 JKH158_nextera_output_R1_paired.fastq --pe1-2 JKH158_nextera_output_R2_paired.fastq -o JKH158_SPAdes_Nextera

spades.py --pe1-1 JKH158_nextera_output_R1_paired.fastq --pe1-2 JKH158_nextera_output_R2_paired.fastq --nanopore JKH158_long_reads_nanofilt_porechopped.fastq.gz -o JKH158_SPAdes_Hybrid_Nextera

# nexera unicycler assemblies

unicycler -1 JKH158_nextera_output_R1_paired.fastq -2 JKH158_nextera_output_R2_paired.fastq -o JKH158_Unicycler_Nextera

unicycler -1 JKH158_nextera_output_R1_paired.fastq -2 JKH158_nextera_output_R2_paired.fastq -l JKH158_long_reads_nanofilt_porechopped.fastq.gz -o JKH158_Unicycler_Hybrid_Nextera

Commands for S3 and S4 Data

#!/usr/bin/perl

#

# find_local_sequence_context.pl

# v1.2 November 1, 2018

# Jonathan Klassen

#

# Uses output of a nucmer comparison of two genomes to identify the local sequence context of all SNP and indel differences

use strict;

use warnings;

open (INSNPS, $ARGV[0]) or die "Cannot open the input nucmer SNP output file from nucmer as \$ARGV[0]\n";

open (INREF, $ARGV[1]) or die "Cannot open the input query genome sequence as \$ARGV[1]\n";

open (INQUERY, $ARGV[2]) or die "Cannot open the input query genome sequence as \$ARGV[2]\n";

my $prefix = $ARGV[2] or die "The prefix for the output files is no \$ARGV[2]\n";

my $kmersize = 5; # define target kmer size to analyze, needs to be an odd number to center on snp/indel positions

###############################################################################

# load reference genome as hash where contig names are keys and seqs are values

###############################################################################

print "Loading reference genome...";

my %refseqs = ();

my $refkey;

while (<INREF>){

if (/^>(\S+)/){

$refkey = $1;

$refseqs{$refkey} = '';

}

else {

s/\s+$//;

$refseqs{$refkey} = $refseqs{$refkey} . $_;

}

}

print "done\n";

###############################################################################

# load query genome as hash where contig names are keys and seqs are values

###############################################################################

print "Loading query genome...";

my %queryseqs = ();

my $querykey;

while (<INQUERY>){

if (/^>(\S+)/){

$querykey = $1;

$queryseqs{$querykey} = '';

}

else {

s/\s+$//;

$queryseqs{$querykey} = $queryseqs{$querykey} . $_;

}

}

print "done\n";

###############################################################################

# determine base frequencies in reference genome

###############################################################################

print "Determining base frequencies in the reference genome...";

my %refkmers = ();

my %refhomopolymers = ();

my %refhomopolymer_lengths = ();

my $homopolymer = '';

foreach my $contig_name (keys %refseqs){

my @contig = split "", $refseqs{$contig_name};

for (my $a = 0; $a <= $#contig; $a++){ # loop through bases in each contig

# determine kmer frequencies in reference genome

unless ($a > $#contig - $kmersize){ # skip last partial kmer

my $kmer = substr $refseqs{$contig_name}, $a, $kmersize;

$refkmers{$kmer}++;

}

# determine homopolymer length distribution in reference genome

if ($homopolymer eq ''){ # begin with first base

$homopolymer = $contig[$a];

}

else {

$homopolymer =~ /(\w)$/;

if ($a == $#contig){ # store at last base

$refhomopolymers{$homopolymer}++;

my $homopolymer_length = length $homopolymer;

$refhomopolymer_lengths{$homopolymer_length}++;

}

elsif ($contig[$a] eq $1){ # extend existing homopolymer

$homopolymer = $homopolymer . $contig[$a];

}

else { # start a new homopolymer

$refhomopolymers{$homopolymer}++;

my $homopolymer_length = length $homopolymer;

$refhomopolymer_lengths{$homopolymer_length}++;

$homopolymer = $contig[$a];

}

}

}

}

###############################################################################

# determine base frequencies in query genome

###############################################################################

print "Determining base frequencies in the query genome...";

my %querykmers = ();

my %queryhomopolymers = ();

my %queryhomopolymer_lengths = ();

$homopolymer = '';

foreach my $contig_name (keys %queryseqs){

my @contig = split "", $queryseqs{$contig_name};

for (my $a = 0; $a <= $#contig; $a++){ # loop through bases in each contig

# determine kmer frequencies in query genome

unless ($a > $#contig - $kmersize){ # skip last partial kmer

my $kmer = substr $queryseqs{$contig_name}, $a, $kmersize;

$querykmers{$kmer}++;

}

# determine homopolymer length distribution in query genome

if ($homopolymer eq ''){ # begin with first base

$homopolymer = $contig[$a];

}

else {

$homopolymer =~ /(\w)$/;

if ($a == $#contig){ # store at last base

$queryhomopolymers{$homopolymer}++;

my $homopolymer_length = length $homopolymer;

$queryhomopolymer_lengths{$homopolymer_length}++;

}

elsif ($contig[$a] eq $1){ # extend existing homopolymer

$homopolymer = $homopolymer . $contig[$a];

}

else { # start a new homopolymer

$queryhomopolymers{$homopolymer}++;

my $homopolymer_length = length $homopolymer;

$queryhomopolymer_lengths{$homopolymer_length}++;

$homopolymer = $contig[$a];

}

}

}

}

print "done\n";

###############################################################################

# load nucmer comparison

###############################################################################

print "Loading nucmer comparison...";

<INSNPS>; # skip header

<INSNPS>;

<INSNPS>;

<INSNPS>;

<INSNPS>;

my %refsnps = ();

my %refindels = ();

my %querysnps = ();

my %queryindels = ();

while (<INSNPS>){

my @line = split /\s+/, $_;

# store snps/indels as hash of arrays, where contigs are key and positions on that contig are values

if ($line[2] eq "." or $line [3] eq "."){

push @{$refindels{$line[14]}}, $line[1];

push @{$queryindels{$line[15]}}, $line[4];

}

else {

push @{$refsnps{$line[14]}}, $line[1];

push @{$querysnps{$line[15]}}, $line[4];

}

}

print "done\n";

###############################################################################

# find SNP contexts in the reference genome

###############################################################################

print "Determining SNP contexts in the reference genome...";

my %ref_snp_kmer_contexts = ();

my %ref_snp_homopolymer_contexts = ();

my %ref_snp_homopolymer_lengths = ();

my $kmer = '';

$homopolymer = '';

foreach my $contig_name (keys %refsnps){

my @contig = split '', $refseqs{$contig_name};

foreach my $position (@{$refsnps{$contig_name}}){

# find kmer context of each snp in the reference genome

if ($position < 2){

$ref_snp_kmer_contexts{"5_truncated"}++;

}

elsif ((length $refseqs{$contig_name}) - $position < 2){

$ref_snp_kmer_contexts{"3_truncated"}++;

}

else {

$kmer = substr $refseqs{$contig_name}, $position - 1 - (($kmersize - 1) / 2), $kmersize;

# note: position in snp table starts at 1, position in array starts at 0

$ref_snp_kmer_contexts{$kmer}++;

}

# find homopolymer context of each snp in the reference genome

$homopolymer = $contig[$position - 1];

my $base = $homopolymer;

for (my $a = $position; $a <= $#contig; $a++){ # extend homopolymer towards 3'

if ($base eq $contig[$a]){

$homopolymer = $homopolymer . $contig[$a];

}

else {

last;

}

}

for (my $a = $position - 2; $a >= 0; $a--){ # extend homopolymer towards 5'

if ($base eq $contig[$a]){

$homopolymer = $contig[$a] . $homopolymer;

}

else {

last;

}

}

$ref_snp_homopolymer_contexts{$homopolymer}++;

my $length = length $homopolymer;

$ref_snp_homopolymer_lengths{$length}++;

}

}

print "done\n";

###############################################################################

# find SNP contexts in the query genome

###############################################################################

print "Determining SNP contexts in the query genome...";

my %query_snp_kmer_contexts = ();

my %query_snp_homopolymer_contexts = ();

my %query_snp_homopolymer_lengths = ();

$kmer = '';

$homopolymer = '';

foreach my $contig_name (keys %querysnps){

my @contig = split '', $queryseqs{$contig_name};

foreach my $position (@{$querysnps{$contig_name}}){

# find kmer context of each snp in the query genome

if ($position < 2){

$query_snp_kmer_contexts{"5_truncated"}++;

}

elsif ((length $queryseqs{$contig_name}) - $position < 2){

$query_snp_kmer_contexts{"3_truncated"}++;

}

else {

$kmer = substr $queryseqs{$contig_name}, $position - 1 - (($kmersize - 1) / 2), $kmersize;

# note: position in snp table starts at 1, position in array starts at 0

$query_snp_kmer_contexts{$kmer}++;

}

# find homopolymer context of each snp in the query genome

$homopolymer = $contig[$position - 1];

my $base = $homopolymer;

for (my $a = $position; $a <= $#contig; $a++){ # extend homopolymer towards 3'

if ($base eq $contig[$a]){

$homopolymer = $homopolymer . $contig[$a];

}

else {

last;

}

}

for (my $a = $position - 2; $a >= 0; $a--){ # extend homopolymer towards 5'

if ($base eq $contig[$a]){

$homopolymer = $contig[$a] . $homopolymer;

}

else {

last;

}

}

$query_snp_homopolymer_contexts{$homopolymer}++;

my $length = length $homopolymer;

$query_snp_homopolymer_lengths{$length}++;

}

}

print "done\n";

###############################################################################

# find indel contexts in the reference genome

###############################################################################

print "Determining indel contexts in the reference genome...";

my %ref_indel_kmer_contexts = ();

my %ref_indel_homopolymer_contexts = ();

my %ref_indel_homopolymer_lengths = ();

$kmer = '';

$homopolymer = '';

foreach my $contig_name (keys %refindels){

my @contig = split '', $refseqs{$contig_name};

foreach my $position (@{$refindels{$contig_name}}){

# find kmer context of each indel in the reference genome

if ($position < 2){

$ref_indel_kmer_contexts{"5_truncated"}++;

}

elsif ((length $refseqs{$contig_name}) - $position < 2){

$ref_indel_kmer_contexts{"3_truncated"}++;

}

else {

$kmer = substr $refseqs{$contig_name}, $position - 1 - (($kmersize - 1) / 2), $kmersize;

# note: position in indel table starts at 1, position in array starts at 0

$ref_indel_kmer_contexts{$kmer}++;

}

# find homopolymer context of each indel in the reference genome

$homopolymer = $contig[$position - 1];

my $base = $homopolymer;

for (my $a = $position; $a <= $#contig; $a++){ # extend homopolymer towards 3'

if ($base eq $contig[$a]){

$homopolymer = $homopolymer . $contig[$a];

}

else {

last;

}

}

for (my $a = $position - 2; $a >= 0; $a--){ # extend homopolymer towards 5'

if ($base eq $contig[$a]){

$homopolymer = $contig[$a] . $homopolymer;

}

else {

last;

}

}

$ref_indel_homopolymer_contexts{$homopolymer}++;

my $length = length $homopolymer;

$ref_indel_homopolymer_lengths{$length}++;

}

}

print "done\n";

###############################################################################

# find indel contexts in the query genome

###############################################################################

print "Determining indel contexts in the query genome...";

my %query_indel_kmer_contexts = ();

my %query_indel_homopolymer_contexts = ();

my %query_indel_homopolymer_lengths = ();

$kmer = '';

$homopolymer = '';

foreach my $contig_name (keys %queryindels){

my @contig = split '', $queryseqs{$contig_name};

foreach my $position (@{$queryindels{$contig_name}}){

# find kmer context of each indel in the query genome

if ($position < 2){

$query_indel_kmer_contexts{"5_truncated"}++;

}

elsif ((length $queryseqs{$contig_name}) - $position < 2){

$query_indel_kmer_contexts{"3_truncated"}++;

}

else {

$kmer = substr $queryseqs{$contig_name}, $position - 1 - (($kmersize - 1) / 2), $kmersize;

# note: position in indel table starts at 1, position in array starts at 0

$query_indel_kmer_contexts{$kmer}++;

}

# find homopolymer context of each indel in the query genome

$homopolymer = $contig[$position - 1];

my $base = $homopolymer;

for (my $a = $position; $a <= $#contig; $a++){ # extend homopolymer towards 3'

if ($base eq $contig[$a]){

$homopolymer = $homopolymer . $contig[$a];

}

else {

last;

}

}

for (my $a = $position - 2; $a >= 0; $a--){ # extend homopolymer towards 5'

if ($base eq $contig[$a]){

$homopolymer = $contig[$a] . $homopolymer;

}

else {

last;

}

}

$query_indel_homopolymer_contexts{$homopolymer}++;

my $length = length $homopolymer;

$query_indel_homopolymer_lengths{$length}++;

}

}

print "done\n";

# calculate total numbers of counts in each dataset

my $totalrefkmers = 0;

my $totalquerykmers = 0;

my $totalrefsnps = 0;

my $totalquerysnps = 0;

my $totalrefindels = 0;

my $totalqueryindels = 0;

my $totalrefhomopolymers = 0;

my $totalqueryhomopolymers = 0;

my $totalrefsnphomopolymers = 0;

my $totalquerysnphomopolymers = 0;

my $totalrefindelhomopolymers = 0;

my $totalqueryindelhomopolymers = 0;

foreach my $kmer (keys %refkmers){ $totalrefkmers += $refkmers{$kmer}; }

foreach my $kmer (keys %querykmers){ $totalquerykmers += $querykmers{$kmer}; }

foreach my $kmer (keys %ref_snp_kmer_contexts){ $totalrefsnps += $ref_snp_kmer_contexts{$kmer}; }

foreach my $kmer (keys %query_snp_kmer_contexts){ $totalquerysnps += $query_snp_kmer_contexts{$kmer}; }

foreach my $kmer (keys %ref_indel_kmer_contexts){ $totalrefindels += $ref_indel_kmer_contexts{$kmer}; }

foreach my $kmer (keys %query_indel_kmer_contexts){ $totalqueryindels += $query_indel_kmer_contexts{$kmer}; }

foreach my $homopolymer (keys %refhomopolymers){ $totalrefhomopolymers += $refhomopolymers{$homopolymer}; }

foreach my $homopolymer (keys %queryhomopolymers){ $totalqueryhomopolymers += $queryhomopolymers{$homopolymer}; }

foreach my $homopolymer (keys %ref_snp_homopolymer_contexts){ $totalrefsnphomopolymers += $ref_snp_homopolymer_contexts{$homopolymer}; }

foreach my $homopolymer (keys %query_snp_homopolymer_contexts){ $totalquerysnphomopolymers += $query_snp_homopolymer_contexts{$homopolymer}; }

foreach my $homopolymer (keys %ref_indel_homopolymer_contexts){ $totalrefindelhomopolymers += $ref_indel_homopolymer_contexts{$homopolymer}; }

foreach my $homopolymer (keys %query_indel_homopolymer_contexts){ $totalqueryindelhomopolymers += $query_indel_homopolymer_contexts{$homopolymer}; }

foreach my $homopolymer (keys %ref_snp_homopolymer_lengths){ $totalrefsnphomopolymers += $ref_snp_homopolymer_lengths{$homopolymer}; }

foreach my $homopolymer (keys %query_snp_homopolymer_lengths){ $totalquerysnphomopolymers += $query_snp_homopolymer_lengths{$homopolymer}; }

foreach my $homopolymer (keys %ref_indel_homopolymer_lengths){ $totalrefindelhomopolymers += $ref_indel_homopolymer_lengths{$homopolymer}; }

foreach my $homopolymer (keys %query_indel_homopolymer_lengths){ $totalqueryindelhomopolymers += $query_indel_homopolymer_lengths{$homopolymer}; }

# add zeros to kmer hashes if kmer appears in one hash put not the other

foreach my $kmer (keys %refkmers){

unless ($ref_snp_kmer_contexts{$kmer}){ $ref_snp_kmer_contexts{$kmer} = 0; }

unless ($ref_indel_kmer_contexts{$kmer}){ $ref_indel_kmer_contexts{$kmer} = 0; }

}

foreach my $kmer (keys %ref_snp_kmer_contexts){

unless ($refkmers{$kmer}){ $refkmers{$kmer} = 0 }

}

foreach my $kmer (keys %ref_indel_kmer_contexts){

unless ($refkmers{$kmer}){ $refkmers{$kmer} = 0 }

}

foreach my $kmer (keys %querykmers){

unless ($query_snp_kmer_contexts{$kmer}){ $query_snp_kmer_contexts{$kmer} = 0; }

unless ($query_indel_kmer_contexts{$kmer}){ $query_indel_kmer_contexts{$kmer} = 0; }

}

foreach my $kmer (keys %query_snp_kmer_contexts){

unless ($querykmers{$kmer}){ $querykmers{$kmer} = 0 }

}

foreach my $kmer (keys %query_indel_kmer_contexts){

unless ($querykmers{$kmer}){ $querykmers{$kmer} = 0 }

}

# add zeros to homopolymer hashes if kmer appears in one hash put not the other

foreach my $homopolymer (keys %refhomopolymers){

unless ($ref_snp_homopolymer_contexts{$homopolymer}){ $ref_snp_homopolymer_contexts{$homopolymer} = 0; }

unless ($ref_indel_homopolymer_contexts{$homopolymer}){ $ref_indel_homopolymer_contexts{$homopolymer} = 0; }

}

foreach my $homopolymer (keys %queryhomopolymers){

unless ($query_snp_homopolymer_contexts{$homopolymer}){ $query_snp_homopolymer_contexts{$homopolymer} = 0; }

unless ($query_indel_homopolymer_contexts{$homopolymer}){ $query_indel_homopolymer_contexts{$homopolymer} = 0; }

}

foreach my $homopolymer (keys %query_snp_homopolymer_contexts){

unless ($queryhomopolymers{$homopolymer}){ $queryhomopolymers{$homopolymer} = 0 }

}

foreach my $homopolymer (keys %query_indel_homopolymer_contexts){

unless ($queryhomopolymers{$homopolymer}){ $queryhomopolymers{$homopolymer} = 0 }

}

foreach my $homopolymer (keys %refhomopolymer_lengths){

unless ($ref_snp_homopolymer_lengths{$homopolymer}){ $ref_snp_homopolymer_lengths{$homopolymer} = 0; }

unless ($ref_indel_homopolymer_lengths{$homopolymer}){ $ref_indel_homopolymer_lengths{$homopolymer} = 0; }

}

foreach my $homopolymer (keys %ref_snp_homopolymer_lengths){

unless ($refhomopolymer_lengths{$homopolymer}){ $refhomopolymer_lengths{$homopolymer} = 0 }

}

foreach my $homopolymer (keys %ref_indel_homopolymer_lengths){

unless ($refhomopolymer_lengths{$homopolymer}){ $refhomopolymer_lengths{$homopolymer} = 0 }

}

foreach my $homopolymer (keys %queryhomopolymer_lengths){

unless ($query_snp_homopolymer_lengths{$homopolymer}){ $query_snp_homopolymer_lengths{$homopolymer} = 0; }

unless ($query_indel_homopolymer_lengths{$homopolymer}){ $query_indel_homopolymer_lengths{$homopolymer} = 0; }

}

foreach my $homopolymer (keys %query_snp_homopolymer_lengths){

unless ($queryhomopolymer_lengths{$homopolymer}){ $queryhomopolymer_lengths{$homopolymer} = 0 }

}

foreach my $homopolymer (keys %query_indel_homopolymer_lengths){

unless ($queryhomopolymer_lengths{$homopolymer}){ $queryhomopolymer_lengths{$homopolymer} = 0 }

}

###############################################################################

# compare SNP kmer context in reference genome to expected

###############################################################################

open (OUTFILE, ">$prefix\_ref_snp_kmer_contexts.tsv") or die "Cannot open output file ref_snp_kmer_contexts.tsv";

print OUTFILE "kmer\toccurrences_at_snps\t\%_all_occurrences_at_snps\toccurrences_in_ref\t\%_all_occurrences_in_ref\tratio_occurrences_at_snps_to_ref\n";

foreach my $kmer (sort keys %refkmers){

print OUTFILE "$kmer\t";

print OUTFILE "$ref_snp_kmer_contexts{$kmer}\t";

my $percent_snp_kmer = ($ref_snp_kmer_contexts{$kmer} / $totalrefsnps) * 100;

print OUTFILE "$percent_snp_kmer\t";

print OUTFILE "$refkmers{$kmer}\t";

my $percent_ref_kmer = ($refkmers{$kmer} / $totalrefkmers) * 100;

print OUTFILE "$percent_ref_kmer\t";

if ($refkmers{$kmer} == 0){ print OUTFILE "kmer_not_in_ref\n"; }

elsif ($ref_snp_kmer_contexts{$kmer} == 0){ print OUTFILE "kmer_not_in_snps\n"; }

else { print OUTFILE $percent_snp_kmer / $percent_ref_kmer, "\n"; }

}

close OUTFILE;

###############################################################################

# compare SNP kmer context in query genome to expected

###############################################################################

open (OUTFILE, ">$prefix\_query_snp_kmer_contexts.tsv") or die "Cannot open output file query_snp_kmer_contexts.tsv";

print OUTFILE "kmer\toccurrences_at_snps\t\%_all_occurrences_at_snps\toccurrences_in_query\t\%_all_occurrences_in_query\tratio_occurrences_at_snps_to_query\n";

foreach my $kmer (sort keys %querykmers){

print OUTFILE "$kmer\t";

print OUTFILE "$query_snp_kmer_contexts{$kmer}\t";

my $percent_snp_kmer = ($query_snp_kmer_contexts{$kmer} / $totalquerysnps) * 100;

print OUTFILE "$percent_snp_kmer\t";

print OUTFILE "$querykmers{$kmer}\t";

my $percent_query_kmer = ($querykmers{$kmer} / $totalquerykmers) * 100;

print OUTFILE "$percent_query_kmer\t";

if ($querykmers{$kmer} == 0){ print OUTFILE "kmer_not_in_query\n"; }

elsif ($query_snp_kmer_contexts{$kmer} == 0){ print OUTFILE "kmer_not_in_snps\n"; }

else { print OUTFILE $percent_snp_kmer / $percent_query_kmer, "\n"; }

}

close OUTFILE;

###############################################################################

# compare indel kmer context in reference genome to expected

###############################################################################

open (OUTFILE, ">$prefix\_ref_indel_kmer_contexts.tsv") or die "Cannot open output file ref_indel_kmer_contexts.tsv";

print OUTFILE "kmer\toccurrences_at_indel\t\%_all_occurrences_at_indel\toccurrences_in_ref\t\%_all_occurrences_in_ref\tratio_occurrences_at_indel_to_ref\n";

foreach my $kmer (sort keys %refkmers){

print OUTFILE "$kmer\t";

print OUTFILE "$ref_indel_kmer_contexts{$kmer}\t";

my $percent_indel_kmer = ($ref_indel_kmer_contexts{$kmer} / $totalrefindels) * 100;

print OUTFILE "$percent_indel_kmer\t";

print OUTFILE "$refkmers{$kmer}\t";

my $percent_ref_kmer = ($refkmers{$kmer} / $totalrefkmers) * 100;

print OUTFILE "$percent_ref_kmer\t";

if ($refkmers{$kmer} == 0){ print OUTFILE "kmer_not_in_ref\n"; }

elsif ($ref_indel_kmer_contexts{$kmer} == 0){ print OUTFILE "kmer_not_in_indels\n"; }

else { print OUTFILE $percent_indel_kmer / $percent_ref_kmer, "\n"; }

}

close OUTFILE;

###############################################################################

# compare indel kmer context in query genome to expected

###############################################################################

open (OUTFILE, ">$prefix\_query_indel_kmer_contexts.tsv") or die "Cannot open output file query_indel_kmer_contexts.tsv";

print OUTFILE "kmer\toccurrences_at_indel\t\%_all_occurrences_at_indel\toccurrences_in_query\t\%_all_occurrences_in_query\tratio_occurrences_at_indel_to_query\n";

foreach my $kmer (sort keys %querykmers){

print OUTFILE "$kmer\t";

print OUTFILE "$query_indel_kmer_contexts{$kmer}\t";

my $percent_indel_kmer = ($query_indel_kmer_contexts{$kmer} / $totalqueryindels) * 100;

print OUTFILE "$percent_indel_kmer\t";

print OUTFILE "$querykmers{$kmer}\t";

my $percent_query_kmer = ($querykmers{$kmer} / $totalquerykmers) * 100;

print OUTFILE "$percent_query_kmer\t";

if ($querykmers{$kmer} == 0){ print OUTFILE "kmer_not_in_query\n"; }

elsif ($query_indel_kmer_contexts{$kmer} == 0){ print OUTFILE "kmer_not_in_indels\n"; }

else { print OUTFILE $percent_indel_kmer / $percent_query_kmer, "\n"; }

}

close OUTFILE;

###############################################################################

# compare SNP homopolymer context in reference genome to expected

###############################################################################

open (OUTFILE, ">$prefix\_ref_snp_homopolymer_contexts.tsv") or die "Cannot open output file ref_snp_homopolymer_contexts.tsv";

print OUTFILE "homopolymer\toccurrences_at_snps\t\%_all_occurrences_at_snps\toccurrences_in_ref\t\%_all_occurrences_in_ref\tratio_occurrences_at_snps_to_ref\n";

foreach my $homopolymer (sort keys %refhomopolymers){

print OUTFILE "$homopolymer\t";

print OUTFILE "$ref_snp_homopolymer_contexts{$homopolymer}\t";

my $percent_snp_homopolymer = ($ref_snp_homopolymer_contexts{$homopolymer} / $totalrefsnps) * 100;

print OUTFILE "$percent_snp_homopolymer\t";

print OUTFILE "$refhomopolymers{$homopolymer}\t";

my $percent_ref_homopolymer = ($refhomopolymers{$homopolymer} / $totalrefhomopolymers) * 100;

print OUTFILE "$percent_ref_homopolymer\t";

if ($refhomopolymers{$homopolymer} == 0){ print OUTFILE "homopolymer_not_in_ref\n"; }

elsif ($ref_snp_homopolymer_contexts{$homopolymer} == 0){ print OUTFILE "homopolymer_not_in_snps\n"; }

else { print OUTFILE $percent_snp_homopolymer / $percent_ref_homopolymer, "\n"; }

}

close OUTFILE;

###############################################################################

# compare SNP homopolymer context in query genome to expected

###############################################################################

open (OUTFILE, ">$prefix\_query_snp_homopolymer_contexts.tsv") or die "Cannot open output file query_snp_homopolymer_contexts.tsv";

print OUTFILE "homopolymer\toccurrences_at_snps\t\%_all_occurrences_at_snps\toccurrences_in_query\t\%_all_occurrences_in_query\tratio_occurrences_at_snps_to_query\n";

foreach my $homopolymer (sort keys %queryhomopolymers){

print OUTFILE "$homopolymer\t";

print OUTFILE "$query_snp_homopolymer_contexts{$homopolymer}\t";

my $percent_snp_homopolymer = ($query_snp_homopolymer_contexts{$homopolymer} / $totalquerysnps) * 100;

print OUTFILE "$percent_snp_homopolymer\t";

print OUTFILE "$queryhomopolymers{$homopolymer}\t";

my $percent_query_homopolymer = ($queryhomopolymers{$homopolymer} / $totalqueryhomopolymers) * 100;

print OUTFILE "$percent_query_homopolymer\t";

if ($queryhomopolymers{$homopolymer} == 0){ print OUTFILE "homopolymer_not_in_query\n"; }

elsif ($query_snp_homopolymer_contexts{$homopolymer} == 0){ print OUTFILE "homopolymer_not_in_snps\n"; }

else { print OUTFILE $percent_snp_homopolymer / $percent_query_homopolymer, "\n"; }

}

close OUTFILE;

###############################################################################

# compare indel homopolymer context in reference genome to expected

###############################################################################

open (OUTFILE, ">$prefix\_ref_indel_homopolymer_contexts.tsv") or die "Cannot open output file ref_indel_homopolymer_contexts.tsv";

print OUTFILE "homopolymer\toccurrences_at_indels\t\%_all_occurrences_at_indels\toccurrences_in_ref\t\%_all_occurrences_in_ref\tratio_occurrences_at_indel_to_ref\n";

foreach my $homopolymer (sort keys %refhomopolymers){

print OUTFILE "$homopolymer\t";

print OUTFILE "$ref_indel_homopolymer_contexts{$homopolymer}\t";

my $percent_indel_homopolymer = ($ref_indel_homopolymer_contexts{$homopolymer} / $totalrefindels) * 100;

print OUTFILE "$percent_indel_homopolymer\t";

print OUTFILE "$refhomopolymers{$homopolymer}\t";

my $percent_ref_homopolymer = ($refhomopolymers{$homopolymer} / $totalrefhomopolymers) * 100;

print OUTFILE "$percent_ref_homopolymer\t";

if ($refhomopolymers{$homopolymer} == 0){ print OUTFILE "homopolymer_not_in_ref\n"; }

elsif ($ref_indel_homopolymer_contexts{$homopolymer} == 0){ print OUTFILE "homopolymer_not_in_indels\n"; }

else { print OUTFILE $percent_indel_homopolymer / $percent_ref_homopolymer, "\n"; }

}

close OUTFILE;

###############################################################################

# compare indel homopolymer context in query genome to expected

###############################################################################

open (OUTFILE, ">$prefix\_query_indel_homopolymer_contexts.tsv") or die "Cannot open output file query_indel_homopolymer_contexts.tsv";

print OUTFILE "homopolymer\toccurrences_at_indels\t\%_all_occurrences_at_indels\toccurrences_in_query\t\%_all_occurrences_in_query\tratio_occurrences_at_indels_to_query\n";

foreach my $homopolymer (sort keys %queryhomopolymers){

print OUTFILE "$homopolymer\t";

print OUTFILE "$query_indel_homopolymer_contexts{$homopolymer}\t";

my $percent_indel_homopolymer = ($query_indel_homopolymer_contexts{$homopolymer} / $totalqueryindels) * 100;

print OUTFILE "$percent_indel_homopolymer\t";

print OUTFILE "$queryhomopolymers{$homopolymer}\t";

my $percent_query_homopolymer = ($queryhomopolymers{$homopolymer} / $totalqueryhomopolymers) * 100;

print OUTFILE "$percent_query_homopolymer\t";

if ($queryhomopolymers{$homopolymer} == 0){ print OUTFILE "homopolymer_not_in_query\n"; }

elsif ($query_indel_homopolymer_contexts{$homopolymer} == 0){ print OUTFILE "homopolymer_not_in_indels\n"; }

else { print OUTFILE $percent_indel_homopolymer / $percent_query_homopolymer, "\n"; }

}

close OUTFILE;

###############################################################################

# compare SNP homopolymer lengths in reference genome to expected

###############################################################################

open (OUTFILE, ">$prefix\_ref_snp_homopolymer_lengths.tsv") or die "Cannot open output file ref_snp_homopolymer_lengths.tsv";

print OUTFILE "homopolymer\toccurrences_at_snps\t\%_all_occurrences_at_snps\toccurrences_in_ref\t\%_all_occurrences_in_ref\tratio_occurrences_at_snps_to_ref\n";

foreach my $length (sort {$a <=> $b} keys %refhomopolymer_lengths){

print OUTFILE "$length\t";

print OUTFILE "$ref_snp_homopolymer_lengths{$length}\t";

my $percent_snp_homopolymer = ($ref_snp_homopolymer_lengths{$length} / $totalrefsnps) * 100;

print OUTFILE "$percent_snp_homopolymer\t";

print OUTFILE "$refhomopolymer_lengths{$length}\t";

my $percent_ref_homopolymer = ($refhomopolymer_lengths{$length} / $totalrefhomopolymers) * 100;

print OUTFILE "$percent_ref_homopolymer\t";

if ($refhomopolymer_lengths{$length} == 0){ print OUTFILE "homopolymer_not_in_ref\n"; }

elsif ($ref_snp_homopolymer_lengths{$length} == 0){ print OUTFILE "homopolymer_not_in_snps\n"; }

else { print OUTFILE $percent_snp_homopolymer / $percent_ref_homopolymer, "\n"; }

}

close OUTFILE;

###############################################################################

# compare SNP homopolymer lengths in query genome to expected

###############################################################################

open (OUTFILE, ">$prefix\_query_snp_homopolymer_lengths.tsv") or die "Cannot open output file query_snp_homopolymer_lengths.tsv";

print OUTFILE "homopolymer\toccurrences_at_snps\t\%_all_occurrences_at_snps\toccurrences_in_query\t\%_all_occurrences_in_query\tratio_occurrences_at_snps_to_query\n";

foreach my $length (sort {$a <=> $b} keys %queryhomopolymer_lengths){

print OUTFILE "$length\t";

print OUTFILE "$query_snp_homopolymer_lengths{$length}\t";

my $percent_snp_homopolymer = ($query_snp_homopolymer_lengths{$length} / $totalquerysnps) * 100;

print OUTFILE "$percent_snp_homopolymer\t";

print OUTFILE "$queryhomopolymer_lengths{$length}\t";

my $percent_query_homopolymer = ($queryhomopolymer_lengths{$length} / $totalqueryhomopolymers) * 100;

print OUTFILE "$percent_query_homopolymer\t";

if ($queryhomopolymer_lengths{$length} == 0){ print OUTFILE "homopolymer_not_in_query\n"; }

elsif ($query_snp_homopolymer_lengths{$length} == 0){ print OUTFILE "homopolymer_not_in_snps\n"; }

else { print OUTFILE $percent_snp_homopolymer / $percent_query_homopolymer, "\n"; }

}

close OUTFILE;

###############################################################################

# compare indel homopolymer lengths in reference genome to expected

###############################################################################

open (OUTFILE, ">$prefix\_ref_indel_homopolymer_lengths.tsv") or die "Cannot open output file ref_indel_homopolymer_lengths.tsv";

print OUTFILE "homopolymer\toccurrences_at_indels\t\%_all_occurrences_at_indels\toccurrences_in_ref\t\%_all_occurrences_in_ref\tratio_occurrences_at_indel_to_ref\n";

foreach my $length (sort {$a <=> $b} keys %refhomopolymer_lengths){

print OUTFILE "$length\t";

print OUTFILE "$ref_indel_homopolymer_lengths{$length}\t";

my $percent_indel_homopolymer = ($ref_indel_homopolymer_lengths{$length} / $totalrefindels) * 100;

print OUTFILE "$percent_indel_homopolymer\t";

print OUTFILE "$refhomopolymer_lengths{$length}\t";

my $percent_ref_homopolymer = ($refhomopolymer_lengths{$length} / $totalrefhomopolymers) * 100;

print OUTFILE "$percent_ref_homopolymer\t";

if ($refhomopolymer_lengths{$length} == 0){ print OUTFILE "homopolymer_not_in_ref\n"; }

elsif ($ref_indel_homopolymer_lengths{$length} == 0){ print OUTFILE "homopolymer_not_in_indels\n"; }

else { print OUTFILE $percent_indel_homopolymer / $percent_ref_homopolymer, "\n"; }

}

close OUTFILE;

###############################################################################

# compare indel homopolymer lengths in query genome to expected

###############################################################################

open (OUTFILE, ">$prefix\_query_indel_homopolymer_lengths.tsv") or die "Cannot open output file query_indel_homopolymer_lengths.tsv";

print OUTFILE "homopolymer\toccurrences_at_indels\t\%_all_occurrences_at_indels\toccurrences_in_query\t\%_all_occurrences_in_query\tratio_occurrences_at_indels_to_query\n";

foreach my $length (sort {$a <=> $b} keys %queryhomopolymer_lengths){

print OUTFILE "$length\t";

print OUTFILE "$query_indel_homopolymer_lengths{$length}\t";

my $percent_indel_homopolymer = ($query_indel_homopolymer_lengths{$length} / $totalqueryindels) * 100;

print OUTFILE "$percent_indel_homopolymer\t";

print OUTFILE "$queryhomopolymer_lengths{$length}\t";

my $percent_query_homopolymer = ($queryhomopolymer_lengths{$length} / $totalqueryhomopolymers) * 100;

print OUTFILE "$percent_query_homopolymer\t";

if ($queryhomopolymer_lengths{$length} == 0){ print OUTFILE "homopolymer_not_in_query\n"; }

elsif ($query_indel_homopolymer_lengths{$length} == 0){ print OUTFILE "homopolymer_not_in_indels\n"; }

else { print OUTFILE $percent_indel_homopolymer / $percent_query_homopolymer, "\n"; }

}

close OUTFILE;

Commands for S6 Data

nucmer cluster_31.fasta cluster_87.fasta -p Cluster_31_vs_87

delta-filter -1 Cluster_31_vs_87.delta > Cluster_31_vs_87.delta.filtered.delta

show-coords Cluster_31_vs_87.delta.filtered.delta > Cluster_31_vs_87_filtered.coords

nucmer cluster_31.fasta cluster_94.fasta -p Cluster_31_vs_94

delta-filter -1 Cluster_31_vs_94.delta > Cluster_31_vs_94.delta.filtered.delta

show-coords Cluster_31_vs_94.delta.filtered.delta > Cluster_31_vs_94_filtered.coords

nucmer cluster_31.fasta cluster_49.fasta -p Cluster_31_vs_49

delta-filter -1 Cluster_31_vs_49.delta > Cluster_31_vs_49.delta.filtered.delta

show-coords Cluster_31_vs_49.delta.filtered.delta > Cluster_31_vs_49_filtered.coords

nucmer cluster_31.fasta cluster_11.fasta -p Cluster_31_vs_11

delta-filter -1 Cluster_31_vs_11.delta > Cluster_31_vs_11.delta.filtered.delta

show-coords Cluster_31_vs_11.delta.filtered.delta > Cluster_31_vs_11_filtered.coords

nucmer cluster_31.fasta cluster_22.fasta -p Cluster_31_vs_22

delta-filter -1 Cluster_31_vs_22.delta > Cluster_31_vs_22.delta.filtered.delta

show-coords Cluster_31_vs_22.delta.filtered.delta > Cluster_31_vs_22_filtered.coords

nucmer cluster_49.fasta cluster_59.fasta -p Cluster_49_vs_59

delta-filter -1 Cluster_49_vs_59.delta > Cluster_49_vs_59.delta.filtered.delta

show-coords Cluster_49_vs_59.delta.filtered.delta > Cluster_49_vs_59_filtered.coords

nucmer cluster_49.fasta cluster_69.fasta -p Cluster_49_vs_69

delta-filter -1 Cluster_49_vs_69.delta > Cluster_49_vs_69.delta.filtered.delta

show-coords Cluster_49_vs_69.delta.filtered.delta > Cluster_49_vs_69_filtered.coords

nucmer cluster_49.fasta cluster_87.fasta -p Cluster_49_vs_87

delta-filter -1 Cluster_49_vs_87.delta > Cluster_49_vs_87.delta.filtered.delta

show-coords Cluster_49_vs_87.delta.filtered.delta > Cluster_49_vs_87_filtered.coords

nucmer cluster_49.fasta cluster_94.fasta -p Cluster_49_vs_94

delta-filter -1 Cluster_49_vs_94.delta > Cluster_49_vs_94.delta.filtered.delta

show-coords Cluster_49_vs_94.delta.filtered.delta > Cluster_49_vs_94_filtered.coords

nucmer cluster_49.fasta cluster_11.fasta -p Cluster_49_vs_11

delta-filter -1 Cluster_49_vs_11.delta > Cluster_49_vs_11.delta.filtered.delta

show-coords Cluster_49_vs_11.delta.filtered.delta > Cluster_49_vs_11_filtered.coords

nucmer cluster_59.fasta cluster_69.fasta -p Cluster_59_vs_69

delta-filter -1 Cluster_59_vs_69.delta > Cluster_59_vs_69.delta.filtered.delta

show-coords Cluster_59_vs_69.delta.filtered.delta > Cluster_59_vs_69_filtered.coords

nucmer cluster_59.fasta cluster_87.fasta -p Cluster_59_vs_87

delta-filter -1 Cluster_59_vs_87.delta > Cluster_59_vs_87.delta.filtered.delta

show-coords Cluster_59_vs_87.delta.filtered.delta > Cluster_59_vs_87_filtered.coords

nucmer cluster_59.fasta cluster_94.fasta -p Cluster_59_vs_94

delta-filter -1 Cluster_59_vs_94.delta > Cluster_59_vs_94.delta.filtered.delta

show-coords Cluster_59_vs_94.delta.filtered.delta > Cluster_59_vs_94_filtered.coords

nucmer cluster_59.fasta cluster_11.fasta -p Cluster_59_vs_11

delta-filter -1 Cluster_59_vs_11.delta > Cluster_59_vs_11.delta.filtered.delta

show-coords Cluster_59_vs_11.delta.filtered.delta > Cluster_59_vs_11_filtered.coords

nucmer cluster_59.fasta cluster_22.fasta -p Cluster_59_vs_22

delta-filter -1 Cluster_59_vs_22.delta > Cluster_59_vs_22.delta.filtered.delta

show-coords Cluster_59_vs_22.delta.filtered.delta > Cluster_59_vs_22_filtered.coords

nucmer cluster_69.fasta cluster_87.fasta -p Cluster_69_vs_87

delta-filter -1 Cluster_69_vs_87.delta > Cluster_69_vs_87.delta.filtered.delta

show-coords Cluster_69_vs_87.delta.filtered.delta > Cluster_69_vs_87_filtered.coords

nucmer cluster_69.fasta cluster_94.fasta -p Cluster_69_vs_94

delta-filter -1 Cluster_69_vs_94.delta > Cluster_69_vs_94.delta.filtered.delta

show-coords Cluster_69_vs_94.delta.filtered.delta > Cluster_69_vs_94_filtered.coords

nucmer cluster_69.fasta cluster_11.fasta -p Cluster_69_vs_11

delta-filter -1 Cluster_69_vs_11.delta > Cluster_69_vs_11.delta.filtered.delta

show-coords Cluster_69_vs_11.delta.filtered.delta > Cluster_69_vs_11_filtered.coords

nucmer cluster_69.fasta cluster_22.fasta -p Cluster_69_vs_22

delta-filter -1 Cluster_69_vs_22.delta > Cluster_69_vs_22.delta.filtered.delta

show-coords Cluster_69_vs_22.delta.filtered.delta > Cluster_69_vs_22_filtered.coords

nucmer cluster_94.fasta cluster_11.fasta -p Cluster_94_vs_11

delta-filter -1 Cluster_94_vs_11.delta > Cluster_94_vs_11.delta.filtered.delta

show-coords Cluster_94_vs_11.delta.filtered.delta > Cluster_94_vs_11_filtered.coords

nucmer cluster_94.fasta cluster_22.fasta -p Cluster_94_vs_22

delta-filter -1 Cluster_94_vs_22.delta > Cluster_94_vs_22.delta.filtered.delta

show-coords Cluster_94_vs_22.delta.filtered.delta > Cluster_94_vs_22_filtered.coords

nucmer cluster_11.fasta cluster_22.fasta -p Cluster_11_vs_22

delta-filter -1 Cluster_11_vs_22.delta > Cluster_11_vs_22.delta.filtered.delta

show-coords Cluster_11_vs_22.delta.filtered.delta > Cluster_11_vs_22_filtered.coords
