# Supplementary material for: Evaluation of strategies for the assembly of diverse bacterial genomes using MinION long-read sequencing
Source: BMC Genomics. 2019 Jan 9;20:23. doi: 10.1186/s12864-018-5381-7 (PMC6325685; doi:10.1186/s12864-018-5381-7)
Supplement: Supplementary file 2 — Figure S1. Summary of Illumina Nextera-based assemblies for Pseudonocardia strains JKS002056, JKS002072, and JKS002128. Figure S2. BRIG analysis for Pseudonocardia strain JKS002128. The Canu+Pilon assembly was used as the reference strain. Each ring represents a different assembly type, Canu+Nanopolish (dark blue), Canu (pink), Unicycler Hybrid (green), Spades Hybrid (teal), Unicycler (orange), and Spades (purple). The inner rings describe GC content (black) and GC skew (purple/green). Figure S3. Analysis showing the ratio of SNPs and Indels present in homopolymeric regions ranging from 1 to 8 basepairs long for each Ps JKS002128, Av JG3, and Fs ARS-166-14 assembly relative to the Canu+Pilon assembly. Results for Ps JKS002128 did not detect SNPs present in homopolymeric regions 7 or 8 basepairs long in either the SPAdes, Unicycler, SPAdes-hybrid, or Unicycler-hybrid assemblies; these data points are therefore missing in this panel. Figure S4. Anvi’o Pangenome display for all strains. The Anvi’o (v.5.2) pangenome pipeline was used following the developer’s pipelines for importing Prokka gene annotations and for performing HMM analyses. Assembly methods are abbreviated as follows: S (SPAdes), U (Unicycler), SH (SPAdes-hybrid), UH (Unicycler-hybrid), P (Canu+Pilon), N (Canu+Nanopolish), and C (Canu). A. Pseudonocardia strains. B. Aeromonas strains. C. Flavobacterium strains. Figure S5. Alignments of Biosynthetic Gene Cluster family 6 (see Fig. 6a). Some Canu-based BGCs were shorter than the less error-prone BGCs annotated on the Illumina-based genomes. (DOCX 2669 kb) [file 12864_2018_5381_MOESM2_ESM.docx]

**Supplemental Figures**

**Supplementary Figure S1:** Summary of Illumina Nextera-based assemblies for *Pseudonocardia* strains JKS002056, JKS002072, and JKS002128.

| **Assembly** | **Contigs** | **Genome length (bp)** | **n50 (bp)** |
| --- | --- | --- | --- |
| ***Ps* JKS002056 SPAdes** | 3,250 | 5,589,123 | 2,619 |
| ***Ps* JKS002056 Unicycler** | 3,157 | 3,867,813 | 2,032 |
| ***Ps* JKS002056 SPAdes-hybrid** | 1,477 | 5,858,159 | 12,343 |
| ***Ps* JKS002056 Unicycler-hybrid** | 78 | 6,444,509 | 3,371,076 |
| ***Ps* JKS002072 SPAdes** | 958 | 6,172,755 | 22,043 |
| ***Ps* JKS002072 Unicycler** | 465 | 5,917,646 | 21,557 |
| ***Ps* JKS002072 SPAdes-hybrid** | 582 | 6,215,045 | 182,949 |
| ***Ps* JKS002072 Unicycler-hybrid** | 9 | 6,138,088 | 2,403,235 |
| ***Ps* JKS002128 SPAdes** | 912 | 6,158,157 | 74,317 |
| ***Ps* JKS002128 Unicycler** | 902 | 5,898,041 | 11,360 |
| ***Ps* JKS002128 SPAdes-hybrid** | 245 | 6,363,541 | 74,317 |
| ***Ps* JKS002128 Unicycler hybrid** | 62 | 6,589,850 | 1,354,853 |

**Supplementary Figure S2:** BRIG analysis for *Pseudonocardia* strain JKS002128. The Canu+Pilon assembly was used as the reference strain. Each ring represents a different assembly type, Canu+Nanopolish (dark blue), Canu (pink), Unicycler Hybrid (green), Spades Hybrid (teal), Unicycler (orange), and Spades (purple). The inner rings describe GC content (black) and GC skew (purple/green).


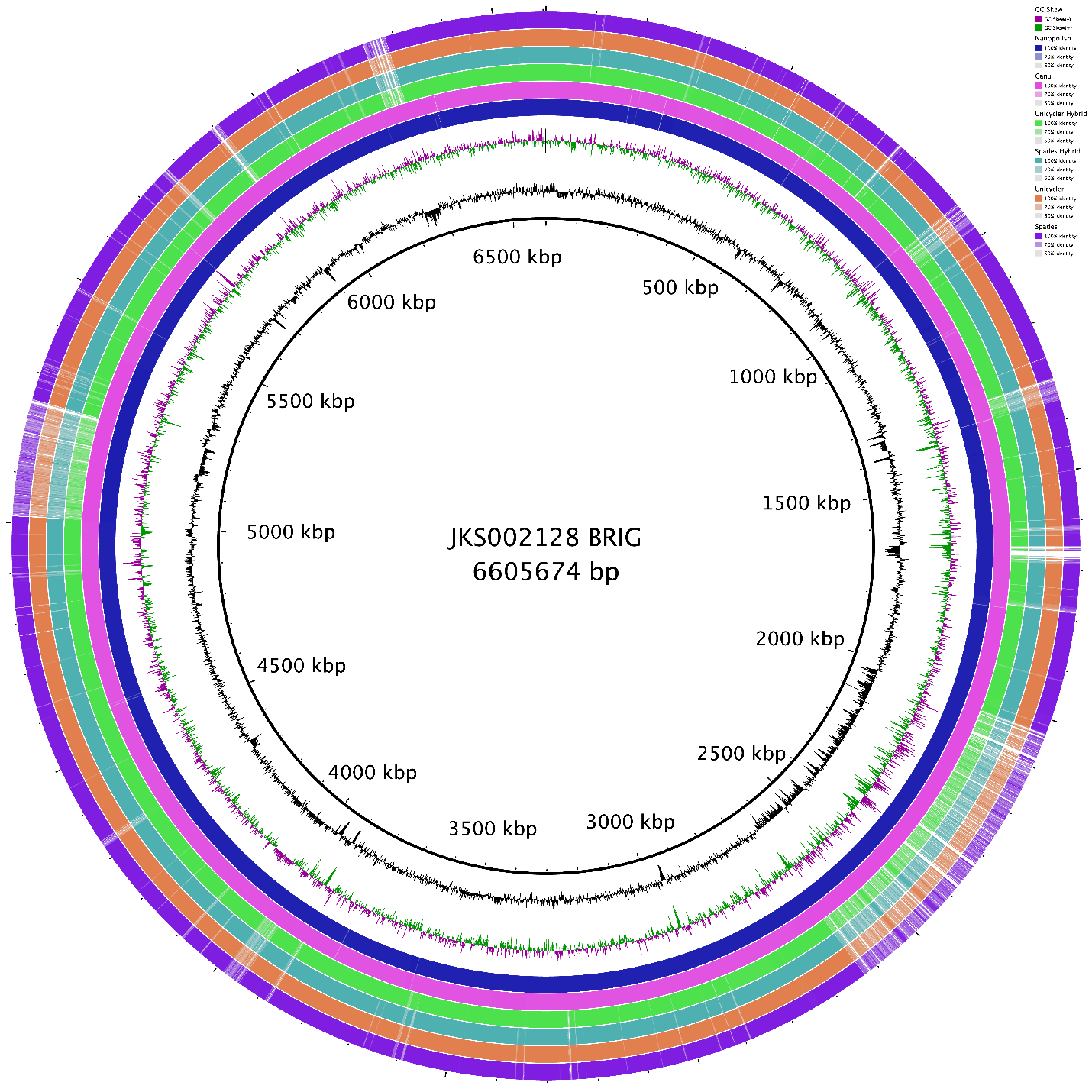


**Supplementary Figure S3:** Analysis showing the ratio of SNPs and Indels present in homopolymeric regions ranging from 1 to 8 basepairs long for each *Ps* JKS002128, *Av* JG3, and *Fs* ARS-166-14 assembly relative to the Canu+Pilon assembly. Results for *Ps* JKS002128 did not detect SNPs present in homopolymeric regions 7 or 8 basepairs long in either the SPAdes, Unicycler, SPAdes-hybrid, or Unicycler-hybrid assemblies; these data points are therefore missing in this panel.


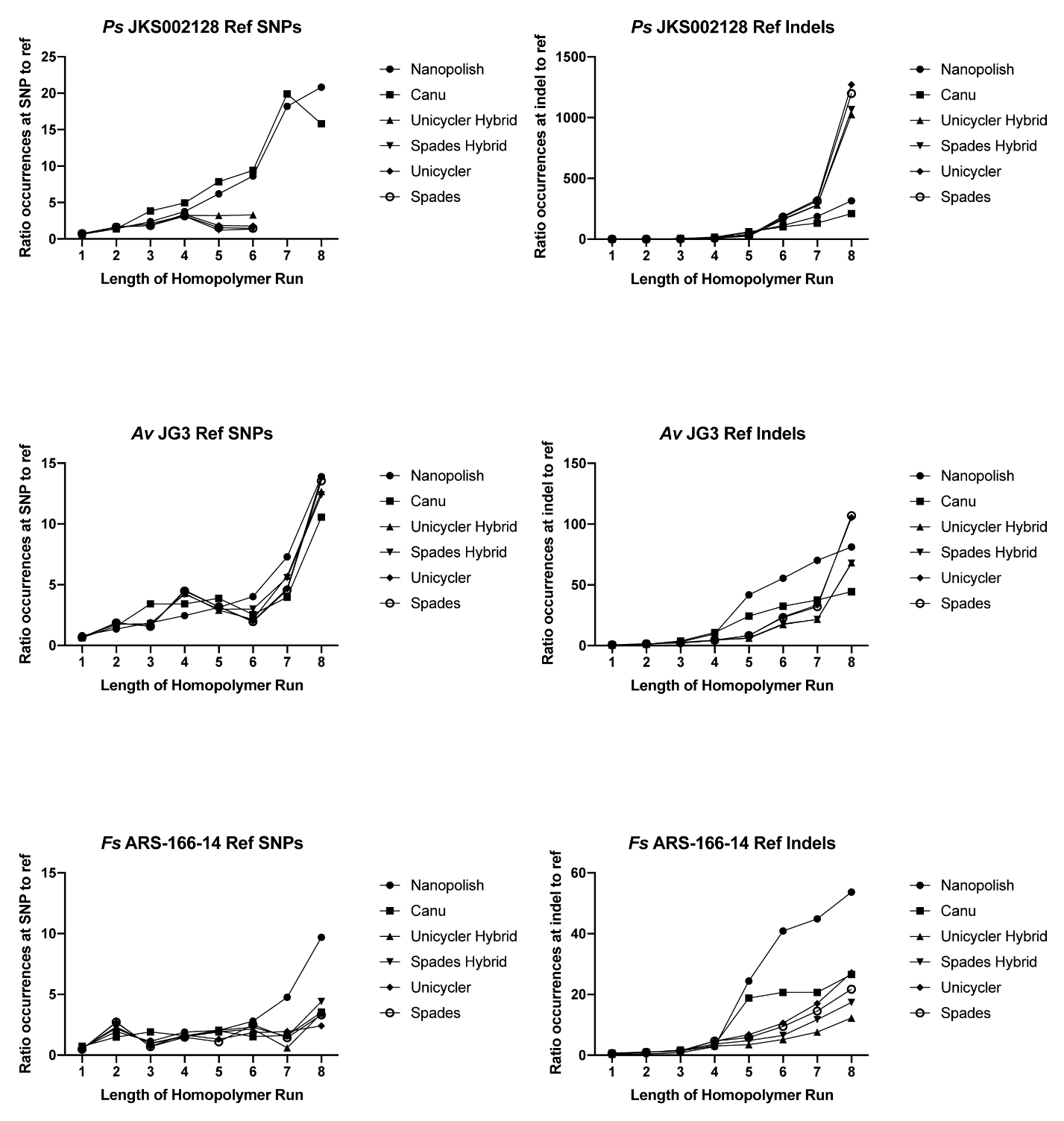


**Supplementary Figure S4:** Anvi’o Pangenome display for all strains. The Anvi’o (v.5.2) pangenome pipeline was used following the developer’s pipelines for importing Prokka gene annotations and for performing HMM analyses. Assembly methods are abbreviated as follows: S (SPAdes), U (Unicycler), SH (SPAdes-hybrid), UH (Unicycler-hybrid), P (Canu+Pilon), N (Canu+Nanopolish), and C (Canu). **A.** *Pseudonocardia* strains. **B.** *Aeromonas* strains. **C.** *Flavobacterium* strains.


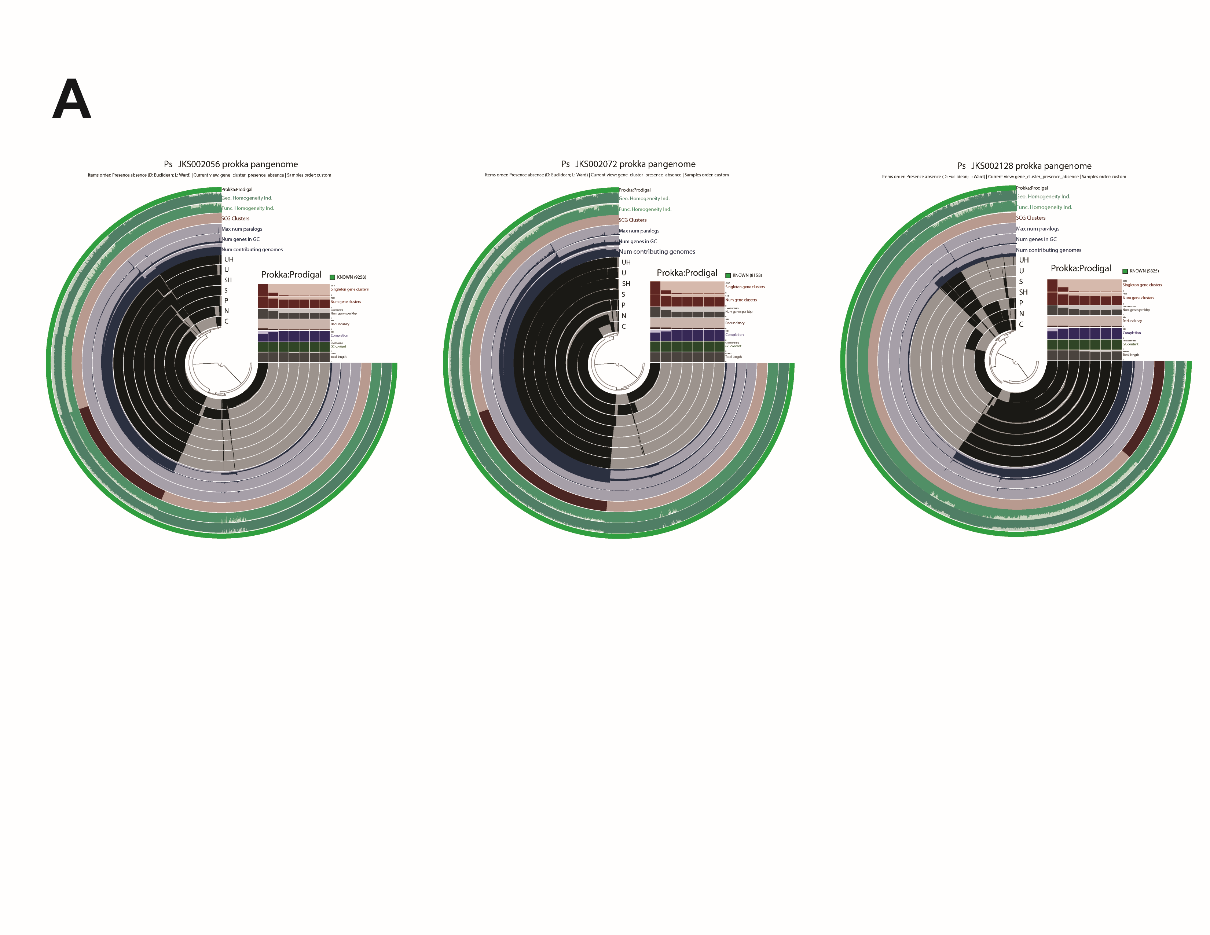


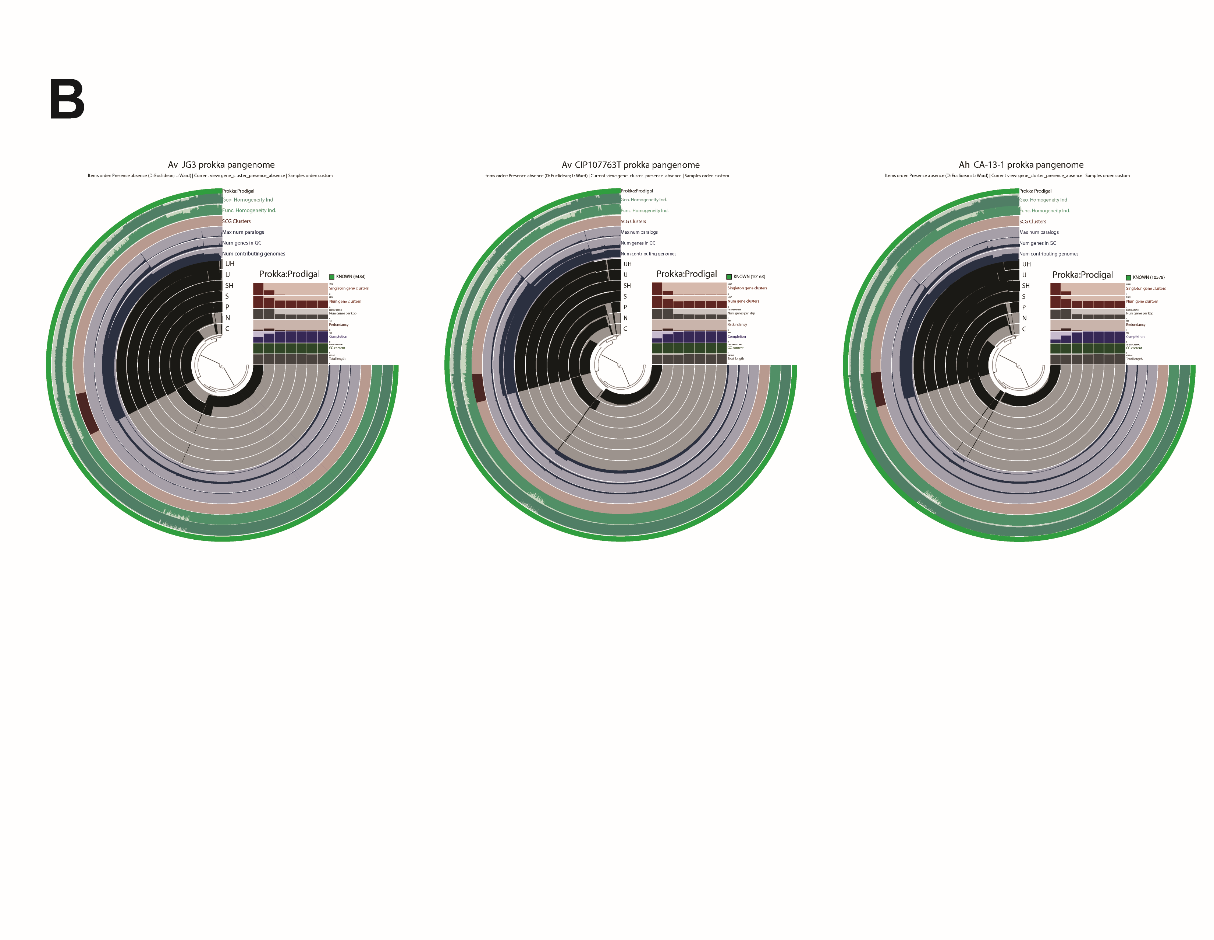


**
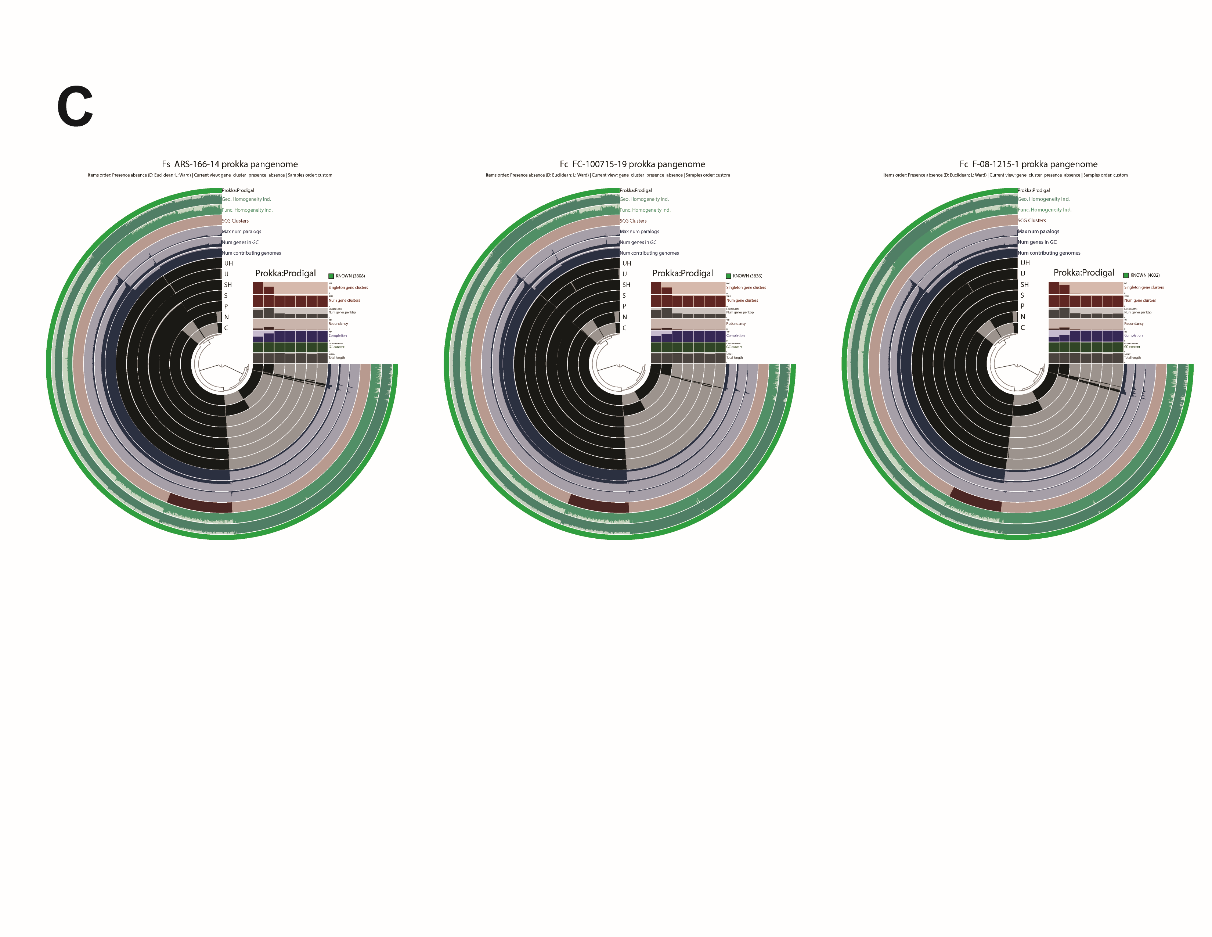
**

**Supplementary Figure S5:** Alignments of Biosynthetic Gene Cluster family 6 (see Figure 6A). Some Canu-based BGCs were shorter than the less error-prone BGCs annotated on the Illumina-based genomes.

**
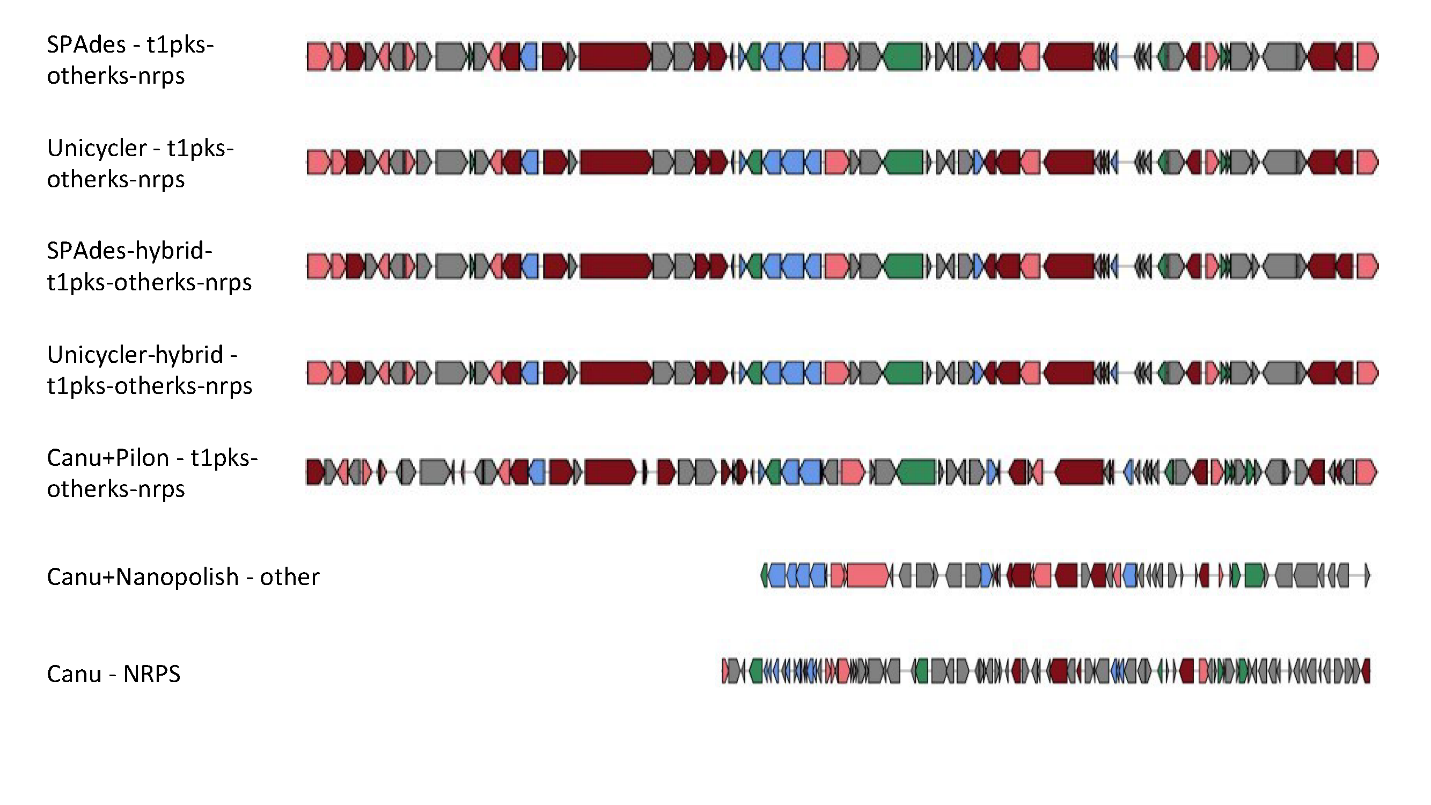
**
